# Sustainable thermal paper formulation using lignocellulosic biomass fractions

**DOI:** 10.1126/sciadv.adw9912

**Published:** 2026-01-02

**Authors:** Tom Nelis, Manon Rolland, Claire L. Bourmaud, Etiënne L. M. Vermeirssen, Ghezae Tekleab, Harm-Anton Klok, Jeremy S. Luterbacher

**Affiliations:** ^1^ Laboratory of Sustainable and Catalytic Processing, Institute of Chemical Sciences and Engineering, École Polytechnique Fédérale de Lausanne (EPFL), CH-1015 Lausanne, Switzerland.; ^2^ École Polytechnique Fédérale de Lausanne (EPFL), Institut des Matériaux and Institut des Sciences et Ingénierie Chimiques, Laboratoire des Polymères, Bâtiment MXD, Station 12, CH-1015 Lausanne, Switzerland.; ^3^ Swiss Centre for Applied Ecotoxicology, Überlandstrasse, 133, 8600 Dübendorf, Switzerland.

## Abstract

Thermal paper presents widely recognized health hazards due to its formulations containing bisphenol A (BPA) and bisphenol S (BPS) as color developers with limited research on safer alternatives. Here, we introduce sustainable thermal paper formulations built with functionalized lignin polymers and lignin-derived esters, combined with a sensitizer derived from xylan. Light-colored lignin polymer was obtained via sequential aldehyde-assisted fractionation, which reduced chromophore concentration through multiple extraction cycles. Good performance was achieved with polymeric lignin (color density at 120°C ≈ 0.8 to 1.1) when combined with xylan-derived diformylxylose (DFX), each of which is produced directly by simple biomass fractionation. Coatings remained stable for over a year under ambient conditions. Last, lignin-based developers showed estrogenic activity that was two to three orders of magnitude lower than BPA and one to two orders of magnitude lower than BPS, while the DFX sensitizer showed no signs of estrogenic activity or toxicity to bacteria or algae.

## Teaser

Safer and more sustainable thermal paper formulations made from nonedible biomass are introduced.

## INTRODUCTION

**Fig. 1.**
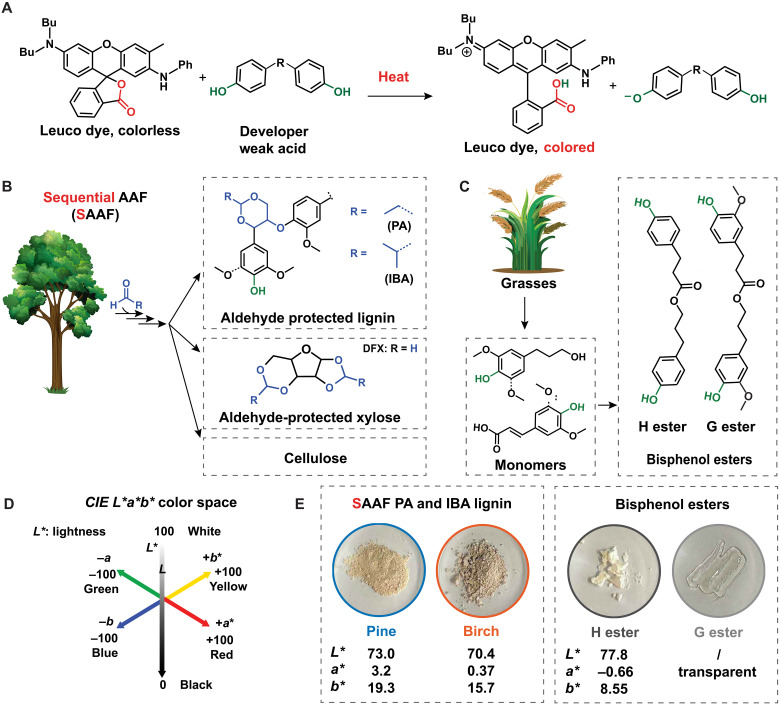
Sustainable thermal paper ingredients platform. (**A**) Color-forming reaction occurring in thermal paper: reaction scheme of traditional bisphenol color developer with the leuco dye (e.g., ODB-2). (**B**) Sequential AAF (SAAF) (*46*, *47*) product platform and (**C**) lignin-derived esters. (**D**) Three-dimensional perception uniform color space representation of CIE L* (lightness) a* (green-red axis) b* (blue-yellow axis). (**E**) Photographs of the lignin-based color developers used in this work and their respective CIE L*a*b* values.

Thermal printing is widely used because of its simplicity and rapid processing, which means that we find the associated thermal paper in products such as point-of-sale receipts, package labels, travel passes, fax paper, medical records, etc. (*1*–*3*). Thermal paper’s global market was valued at 4 billion dollars in 2022 and is expected to reach about 6 billion by 2030 (*4*). Formulations for this paper comprise a thermosensitive layer containing a thermochromic fluoran-type dye (leuco dye) and a color developer. When exposed to heat, the thermal layer melts, bringing the dye and developer into contact. The lactone ring of the dye opens through protonation by the weakly acidic developer, evolving into a colored cationic fluoran structure with an extended conjugated double-bond system (Fig. 1A) (*5*). Traditionally, these formulations also contain a sensitizer, present at levels ranging from 75 to 200% compared to the dye content, which plays a crucial role in fine-tuning the temperature at which the color change occurs (*6*, *7*). Specifically, sensitizers are thought to act as solvents, in which the developer and dye dissolve below their melting points, facilitating their contact and subsequent proton transfer, and hence color development. Because of its cost-effectiveness and efficacy, bisphenol A (BPA) has been extensively used as a color developer, with concentrations reaching up to 42.6 mg of BPA per gram of thermal paper, equivalent to 35 mg of BPA per normal size cash receipt (i.e., with dimensions of 7.5 cm by 20 cm) (*8*, *9*). There is compelling evidence for BPA’s endocrine-disrupting properties (which are associated with reproductive issues, cancer, obesity, etc.) and other toxicities (*10*–*14*). As a result, since 2020, the European Union has imposed restrictions to BPA content in thermal paper, limiting it to less than 0.02 wt %, followed by a ban of BPA in food contact materials in 2024 (*15*, *16*). These regulations have led to the substitution of BPA by other (bis)phenols, such as bisphenol S (BPS) and 4-hydroxy-4′-isopropoxydiphenylsulfone, which were recently revealed to exhibit toxicological properties similar to BPA (*17*–*20*). Because of this toxicity, BPS and other bisphenol alternatives have even been referred to as “regrettable alternatives” (*21*). Despite the identified risks and recent regulations to limit the use of BPA and/or BPS in thermal paper, the transition away from these developers is occurring at a slow pace and varies substantially by country (table S1). While, in some regions, the BPA ban seems to be very effective, the global transition away from BPA is rather slow and inconsistent (*8*, *22*–*26*).

Human exposure to thermal paper developers can occur through food, (trans)dermal absorption, hand-to-mouth transfer, etc. Dermal absorption can also be increased up to a 100-fold through the simultaneous use of penetration enhancing chemicals that include products such as hand sanitizers or skin care products (*27*, *28*). Recent studies have shown that BPS and other developers migrate from thermal labels into plastic-wrapped food at levels exceeding the European Union specific migration limit (50 ng g^−1^) (*29*). The use of BPA in thermal paper has also led to other sources of contamination such as during paper recycling where only some of the BPA is removed, while the remaining fraction pollutes the resulting recycled paper (*30*). As a result, nonnegligible quantities of BPA were detected in products such as food contact papers, newspapers, napkins, and toilet paper (*31*). In this context, there is an increasing interest in safer, well-assessed, biodegradable, and affordable chemicals for thermal developer applications.

Efforts to reduce toxicity have included approaches by An and co-workers (*32*–*34*) to increase molecular weight of developers by synthesizing two types of polymeric phenolics: a BPA-based polymer with formaldehyde bridges and a phloroglucinol-based polymer with acyl chloride linkers. However, these fossil-based polymers were not subjected to toxicological tests. Part of the motivation of these substrates is because skin absorbability reduces significantly with increasing developer molecular size or volume. To allow for skin absorption, the molecular weight of a molecule must be below 500 Da (*35*, *36*).

This work led us to consider lignin as a potential substitute for phenolic developer. Lignin, a major component of lignocellulosic biomass (15 to 30 wt %), is a naturally polyphenolic material [molecular weight > 500 Da with a phenolic p*K*_a_ of ~9 to 10 (where *K*_a_ is the acid dissociation constant), similar to BPA/BPS] (*37*), and its natural structure is likely to be significantly less toxic than BPA and fossil alternatives. On the basis of structure-toxicity relationships established in vitro, *ortho*-substituents (*o*-methoxy moieties) and polar substituents on the bridge connecting both phenols would lower estrogenic activity (*38*). Being composed out of *p*-coumaryl, coniferyl, and sinapyl monomers, lignin contains plenty of *o*-methoxy moieties and polar substituents (*39*). In addition, lignin is widely available at relatively low cost (*40*–*43*). However, commercial or “technical” lignin, which is a by-product of the pulp industry, suffers from high coloration due to its condensed nature (*44*, *45*). This particular property poses a challenge for its direct use in printing applications, typically requiring light coloration for good contrast.

In past work, we have developed the aldehyde-assisted fractionation (AAF) process that uses aldehyde to functionalize lignin β-O-4 linkages and prevents condensation during an organosolv pretreatment. As a result, the lignin is closer to its native structure, less cross-linked, and generally less colored than technical lignins (*46*, *47*). Here, we propose a modified AAF procedure involving multiple extraction cycles to produce even more lightly colored lignin (Fig. 1B). The resulting polymeric aldehyde-functionalized lignin was studied for direct use as a developer. We also evaluated lignin-derived esters to show the potential of small molecules derived from lignin (Fig. 1C). We formulated thermoresponsive coatings using a protocol found in the patent literature, which incorporated sensitizers (*48*, *49*). Conventional sensitizers are fossil based, typically include an aromatic ring, and have the potential to accumulate in aquatic organisms, resulting in ecosystem disruption (*6*, *7*). We explored the use of acetal-stabilized sugars, simultaneously extracted from the hemicellulose fraction (20 to 40 wt %) during the AAF process (Fig. 1B), which have been shown to be useful as solvents, surfactants, or platform chemicals (*50*–*53*). Overall, we present more sustainable and safer thermal paper formulations that use two major biomass fractions from the same process.

## RESULTS

### Lignin-based color developer preparation

Commercial lignin (e.g., Kraft lignin; fig. S1A) is dark colored. To quantify this coloration, we used the CIE L*a*b* color space (as defined by the Commission Internationale d’Éclairage) (Fig. 1D), which breaks down coloration into three dimensions: the a* value representing the red-green spectrum, the b* value representing yellow-blue spectrum, and the L* value describing perceptual lightness (see text S1.6 for further details). Kraft lignin has a lightness value of L* = 49.3 [L* about halfway between 0 (black) and 100 (white); table S2]. During classical lignin extraction such as the Kraft process, condensation reactions induce various chromophores (*44*, *54*), leading to very poor contrast when used as a thermal coating (vide infra). The AAF process is known to limit these condensation reactions (*46*, *47*). Softwood lignins extracted using the AAF process exhibited moderate coloration (L* ≈ 61 to 63 and a* ≈ 0.6 to 3.1), while hardwood AAF lignins were characterized by a purple color, indicated by higher-positive a* values, indicating redness (L* ≈ 59 to 60 and a* ≈ 14.3; Fig. 1D, fig. S1B, and table S2). To produce even more lightly colored lignin, we designed a sequential AAF (SAAF) extraction method, where lignin is extracted through multiple 1-hour AAF cycles, instead of one single 3-hour extraction (process overview is schematized in fig. S2). After three extraction cycles of 1 hour, both softwood (pine) and hardwood (birch) lignin, protected by propionaldehyde (PA) and isobutyraldehyde (IBA), respectively, presented a light-beige color with reduced redness (L* = 68 to 73 and a* ≈ 0.3 to 3; Fig. 1E, fig. S1C, and table S2).

Many functional groups within the lignin act as chromophores, for instance, unsaturated ketones and carboxylic acids, often found as terminal groups of the lignin backbone (*55*). The presence of these chromophores was investigated using Fourier transform infrared spectroscopy following past work (*56*–*58*). With increasing number of extraction cycles (1 to 3 cycles), we observed a linear decrease in the intensity of peaks associated with α,β-unsaturated ketones (1668 to 1685 cm^−1^) (fig. S3A and table S3). This decrease in chromophore results into lightening of the lignin color from dark purple to clear pink and lastly to light beige (fig. S3B).

The increase in extraction cycles correlates with an increase in molecular weight of the extracted lignin polymers, as observed by size exclusion chromatography and was associated with a lower phenolic OH content, since these functional groups are predominantly located on terminal units (table S3). This gradual increase in molecular weight has been previously observed in organosolv processes, where shorter and more-soluble lignin fragments were thought to be extracted first, while longer and more tightly bound polymers required extended reaction times (*59*–*61*). A higher molecular weight likely results in a decreased number of terminal chromophores and overall lignin brightness. When in solution, the distinct color variations of the SAAF lignin powders remains evident, as characterized with ultraviolet-visible spectroscopy (fig. S4). These high–molecular weight lignins not only exhibit a bright color (L* > 68; Fig. 1E, fig. S1C, and table S2), which is essential for achieving good contrast, but also minimize the possibility for dermal absorption (*M*_n_ = 1422 to 3136 Da; fig. S5 and table S4), making them ideal candidates for thermal paper formulations. Regarding economics, while a specific technoeconomic analysis for SAAF lignin has not been performed yet, early-stage technoeconomic assessment of the AAF biorefinery has estimated that the AAF lignin by-product can be sold at values between $0.6 and $0.94 kg^−1^ (*62*). The sequential extraction cycles would inevitably increase the production costs, but the margin with the current market prices for BPA and BPS ($1.5 to $2 kg^−1^) could realistically absorb the additional expenses (*63*).

For comparison, we also wanted to synthesize close analogs to bisphenol from lignin-derived monomers (*38*). Dihydroferulic acid and dihydro-*p*-coumaric acid, obtained from, e.g., grasses (*64*) and endocarps (*65*), were esterified with dihydroconiferyl alcohol and 3-(4-hydroxyphenyl)-1-propanol to reach the “G” (for guaiacol) and “H” (for hydroxy) ester, respectively (Fig. 1C and text S2.4). The G ester is one of the several bisphenolic dimers that could be extracted from certain plants (*66*). In addition, the H and G esters help test the hypothesis that the presence of a natural *o*-methoxy moiety, along with polar substituents on the connecting bridge (i.e., the ester moiety), may reduce estrogenic activity (vide infra) (*38*). These molecules achieve comparable and competitive performance in terms of contrast (white/transparent compounds; Fig. 1E), likely being able to replace BPA without further modifications. Functionalized lignin polymers can also likely replace BPA in thermal paper but will require careful product formulation.

### Color activity screening and thermal stability

Before performing a coating on paper, which is the most relevant to industrial applicability, we applied a rapid test to screen for the color-forming ability of SAAF and other lignin polymers (*33*, *34*). This simple test was indicative of the proton transfer efficiency of the phenolic developers by combining the two crucial components, i.e., developer and dye, within an octadecanol binder, while avoiding the complexity of a full paper coating. For this, we maintained a constant molar “phenol:dye” ratio based on phosphorus-31 nuclear magnetic resonance quantification (table S5 and color activity test in Materials and Methods for the detailed protocol) (*67*). In this experiment, color formation was quantified using a colorimeter, measuring color density (C.D.) via the *Y* parameter. The *Y* luminance parameter is analogous to L* (and they can be calculated from one another) but is defined from a different color space, which is older than the L*a*b space but still used by industry (see details in text S1.6). The C.D., ranging from 0 to 2.5 using the colorimeter’s white/black calibrant, represents the visual intensity and richness of a color, which, in turn, can be used to characterize contrast change on paper during the heating/printing process (see Materials and Methods). While this test has no specific C.D. that would correspond to an industrial threshold, it was designed to differentiate relative performance and to identify promising candidates for further testing in real coatings.

**Fig. 2.**
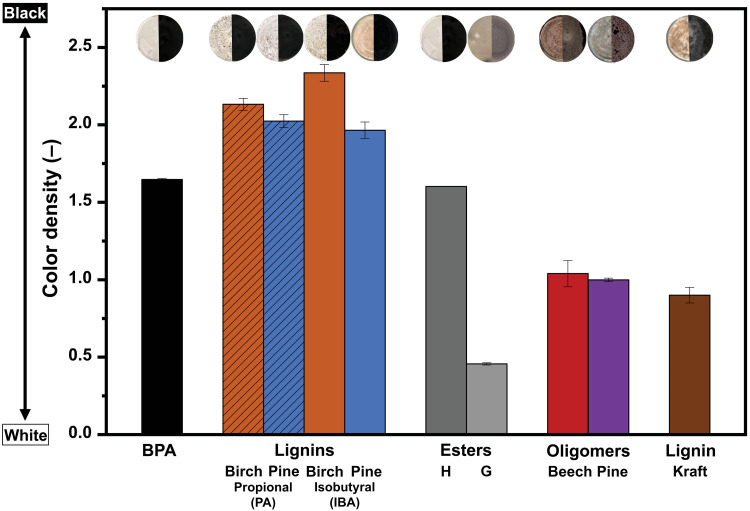
Color activity test. Color development test performed at 100°C, in octadecanol, while maintaining a constant molar phenolic ratio (developer phenolic molar eq.:dye ODB-2 = 1:1). We include the corresponding photography before and after color development.

The BPA reference switched from white to black after heating, reaching a final C.D. of 1.65. All SAAF lignins demonstrated excellent color activity, with C.D.s exceeding those developed by BPA and control Kraft lignin samples [C.D. (SAAF lignins) = 2 to 2.13 > C.D. (BPA) = 1.65 > C.D. (Kraft lignin) = 0.90; table S6], confirming successful proton transfer from polymeric lignin to the dye. The images before and after heating (Fig. 2) suggested that initial coloring of lignin samples, especially for Kraft lignin, would make contrast a larger challenge than for typical BPA formulations.

The bio-based H ester showed almost identical results in terms of C.D. and visual contrast to BPA (Fig. 2 and table S6). In contrast, the G ester sample presented a much lower reactivity with a final C.D. of 0.45 (control octadecanol and dye C.D. = 0.41; Fig. 2 and table S6). The lower reactivity of the G ester can be explained by the presence of the methoxy substituent *ortho* to the phenol. While methoxy groups are inductively electron withdrawing, the phenolic acidity is decreased by the predominant resonance-donating effect. Unexpectedly, the clear effect of methoxy substitution in these small molecules did not translate to the polymeric lignin reactivity, which contains mostly *ortho*-methoxy substituents near its phenolic groups. Other factors such as variations on the SAAF lignin or the nature of the protecting aldehyde (PA or IBA) or wood nature (birch or pine) seemed to have minimal influence on color development in this test.

For comparison, we also tested so-called “lignin oligomers” that are obtained after depolymerization of isolated lignin in lignin processes, which have relatively lower molecular weight (*M*_n_ = 543 to 911 Da; fig. S5 and table S4), therefore presenting more phenolic moieties (end-group) per gram of material (table S5) (*68*). These oligomers are also currently underused in lignin-first strategies (*69*). However, oligomers appeared to have poor reactivity, similar to Kraft lignin, with C.D.s slightly above 1, suggesting a weaker proton-donating ability (Fig. 2). This weak reactivity led us to conclude that the density of phenolic groups was likely not a determining factor in color development performance. An alternative hypothesis to explain the high SAAF lignin reactivity was their higher carboxylic content (acidic protons). In comparison, oligomers tend to retain lower concentrations of carboxylic acid following hydrogenolysis lignin depolymerization (table S5). To investigate this effect, we selected model compounds that contained either both a phenolic and carboxylic (fig. S7A), solely carboxylic (fig. S7B), solely aliphatic alcohol (fig. S7C), or phenolic and aliphatic alcohol groups (fig. S7D) and studied their color development while maintaining the molar “developer:dye” ratio constant. The presence of both phenolic and carboxylic group resulted in the most significant color change (C.D. = 1.68), followed by the compound containing phenolic and aliphatic alcohol group (C.D. = 1.48). Conversely, the compounds containing only a carboxylic group (C.D. = 0.73) or only an aliphatic alcohol showed little activity (C.D. = 0.50). Although the p*K*_a_ of the carboxylic acid group (p*K*_a_ ≈ 4.5) is much lower than the phenolic group (p*K*_a_ ≈ 10), these results suggest that the presence of a carboxylic acid does not actively participate in proton transfer and lactone opening. To confirm this observation, we prepared AAF lignin with the difunctional aldehyde glyoxylic acid (GA) to successfully introduce more carboxylic acid functionalities onto the lignin backbone (more than doubling the carboxylic acid content compared when PA is used; table S5 and text S2.1). Consistent with what was observed with model compounds, this lignin performed poorly as compared to the SAAF lignin, resulting in weak color development (C.D. of GA AAF lignin = 0.7 < C.D. of SAAF lignin ≈ 2; table S6).

We observed the same reactivity trend when a 2:1 mass ratio was used for all developers, although, on a mass basis, lignins contribute significantly less phenolic groups than bisphenols. BPA, the H ester, and SAAF lignins consistently showed high reactivity, while the G ester, Kraft lignin, and oligomer samples remained less reactive (fig. S9 and table S7). We also observed that the less effective lignins, such as the oligomers, Kraft lignin, and AAF GA lignin samples, were fairly heterogeneous, showing significant phase separation domains within the octadecanol matrix (Fig. 2 and fig. S8). These observations suggest that the reaction was not merely controlled by the quantity of available proton donors but more so by the extent of interfacial contact between dye and developer. Thus, another important parameter to consider is the surface area of contact between the dye and the developer. This is well illustrated in commercial formulations, where a developer:dye mass ratio of 2:1 is often used to ensure an excess of developer and maximize contact (*70*). From all these data, we concluded that the primary factor influencing the color developing activity of the developer was its compatibility with both the amphiphilic octadecanol and the dye (a phenomenon that we further discuss for the paper formulations—vide infra). This behavior is reminiscent of mechanisms observed in polymer photoresist systems, where reactivity is influenced not only by functional group density but also by dispersion and diffusion (*71*). The carboxylic acid group content likely plays a role in tuning this compatibility, enabling better dispersion and controlling phase separation. For these reasons, SAAF lignins with their preserved functionality and decent phenolic content are promising developer candidates.

Thermogravimetric analysis under air was used to evaluate the thermal stability of each compound. All lignin-derived developers were stable up to 170°C (*T*_d,95%_) (fig. S10A), making them suitable for thermal printing that generally runs up to 140°C, during which any release of volatile organic material must be avoided (*34*). Furthermore, holding developers at 140°C for 10 min only resulted in negligible mass change (<2 wt %) (fig. S10B). Differential scanning calorimetry analysis confirmed a melting point of BPA near 155°C and showed a melting point for the H ester near 90°C. For the lignin color developers, no melting was observed in the range of 0 to 170°C (fig. S11).

### Thermal paper formulation and performance

**Fig. 3.**
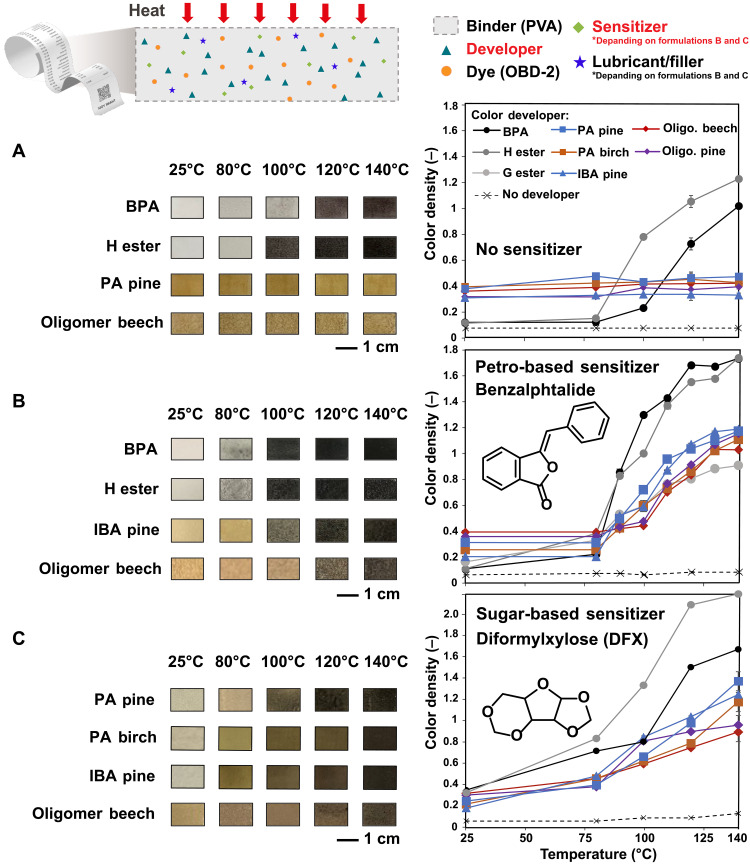
Thermal paper formulations. Static sensitivity curves of thermal papers coated on PlanoJet paper (200 g m^−2^) (**A**) without sensitizer (text S2.7 and formulation S1), (**B**) with benzalphtalide (text S2.7 and formulation S2), and (**C**) with DFX as the sensitizer (56 dry wt %; text S2.7 and formulation S4). The pictures show the developed thermal papers for various heating temperatures (see detailed imaging protocol in text S2.7). Dashed lines represent control experiments with identical formulations but without any developer.

Beyond these initial tests, we evaluated the performance of lignin-based developers in real paper-based formulations. Commercial thermal paper has a thermal layer in which active ingredients are embedded in a matrix with a polyvinyl alcohol (PVA) binder, along with additional additives to enhance smoothness and paper reactivity. To quantitatively evaluate thermal paper performance, we measured the initial temperature for color development, as well as the temperature for reaching the maximum C.D. This behavior is collectively known as static sensitivity, in contrast to dynamic sensitivity that describes how quickly the color develops under thermal conditions. Typically, at 120°C, this maximum should reach above C.D. = 1.1 (*72*). PVA solutions containing the developer and the 2-anilino-6-dibutylamino-3-methylfluoran (ODB-2) dye were coated on white paper (2:1 developer:dye mass ratio, average coating thickness of 75 ± 5 μm; fig. S12). The C.D. was then monitored while exposing the paper to increasing temperatures (from room temperature to 140°C, with 20°C increments) using a temperature-controlled heat gun. The bio-based H ester outperformed commercial BPA as a developer with a C.D. (at 120°C) of 1.1 compared to 0.7, respectively (Fig. 3A). Unexpectedly, despite their high activity in the color activity test (vide supra; Fig. 2), SAAF lignin candidates showed no significant color change (Fig. 3A). We hypothesized that the amphiphilic chemical structure of lignin polymers, via strong intermolecular hydrogen bonds, may lead to preferential interaction with the PVA formulation matrix (*73*, *74*) rather than the dye (*75*) (i.e., a “shielding effect”). The experiment was thus repeated by adding a sensitizer to the formulation, melting upon heating to enhance dye-developer interaction at its melting temperature. We selected two commercial sensitizers with a melting point around 100°C: benzalphtalide and diphenylsulfone. Both sensitizers comprise an aromatic structure and are expected to interact well with phenolic developers notably through π-π electron stacking interactions, without triggering notable color development. Lack of any color development due to the sensitizer alone was confirmed by control experiments, showing no significant color formation in the absence of the developer upon heating (Fig. 3 and fig. S16, black dashed line). To improve the processability, we also added traditional texturing additives calcium carbonate (CaCO_3_) and zinc stearate to the formulation (20 to 30 wt % and 4 to 7 wt %, respectively), which did not affect dye lactone ring opening (fig. S13). Static sensitivity tests showed significant color development with all lignin materials, confirming the necessity to enhance the interaction between ODB-2 and lignin with a sensitizer (Fig. 3B for benzalphtalide and fig. S16 for diphenylsulfone). Furthermore, the order of ingredient addition to the formulation was also found to be crucial: When premixing PVA and lignin materials together, before further addition of other ingredients including the sensitizer, milder color change occurred during the static sensitivity test, further suggesting a shielding effect of the PVA matrix on the lignin (fig. S17). This sequential mixing is often required in thermal paper formulation for enhanced color development (*48*, *49*).

Unlike the formulations without sensitizer, C.D. trends for the lignin coatings containing a sensitizer were consistent with the equimassic results from the color activity test conducted in octadecanol (fig. S9): SAAF lignins showed slightly lower reactivity than BPA and the H ester, but a higher reactivity compared to oligomers, when comparing C.D.s at the same temperatures (Fig. 3B). Varying the dye-to-developer ratio from a ratio of 1:2 to 2:1 revealed no significant change in color development intensity across this range (fig. S18). This result further suggested that the limiting factor in the color development reaction was not the stoichiometry of proton donors (at least at these ratios) but rather the diffusion and compatibility between the dye and the developer within the matrix, meaning that, as our earlier results also suggest (see Fig. 2 and figs. S8 and S9), intermolecular compatibility effects could largely compensate for differences in acidic proton concentration.

**Fig. 4.**
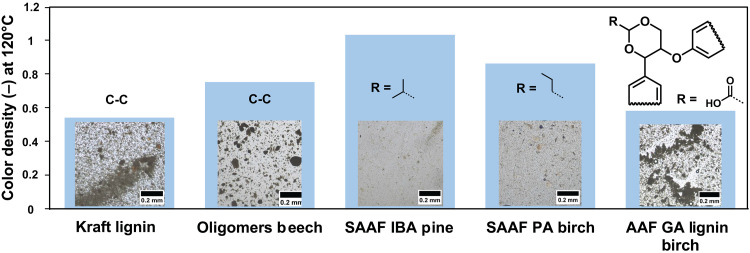
Lignin developer–PVA compatibility. C.D. at 120°C with benzalphtalide as sensitizer and microscopy image of various lignin-PVA blends showing the impact of the blending compatibility of each developer within the PVA matrix (lignin, 19 dry wt %).

Because of the importance of compatibility, the homogeneity of the lignin-based thermal paper formulations was studied with optical microscopy (Fig. 4). AAF IBA and PA birch lignins were well dispersed within the PVA matrix (average size of the phase-separated domains ≈ 0.006 mm ± 0.003 and 0.007 mm ± 0.003), whereas Kraft lignin, GA AAF lignin, and oligomers exhibited phase separation (average size ≈ 0.048 mm ± 0.046, 0.132 mm ± 0.05, and 0.049 mm ± 0.041; table S8). These results demonstrate that functionalized lignins with preserved natural functionality could be formulated into a high-performance bio-based color developer primarily due to their light coloration and excellent compatibility in thermal layer formulations.

### A sugar-based sensitizer

Because commercial sensitizers, which represent a major ingredient of thermal paper formulations, can be toxic and persist in the environment (*6*, *7*), we investigated the use of acetal-stabilized sugars as potential bio-based, low-toxicity, and biodegradable sensitizers. Specifically, we tested diformylxylose (DFX) extracted from the hemicellulose fraction during AAF (Fig. 1B). DFX is a white solid powder that melts at 48°C and a polar aprotic solvent with good compatibility with lignin (*76*). In prior work, we demonstrated that DFX production can be cost-efficient (especially compared to alternate sensitizers) when produced from d-xylose and corn cobs (with an estimated minimum selling price of $1.5 kg^−1^) (*62*). We prepared three formulations with increasing DFX loading (21 to 35 to 56 wt %) using PA lignin (pine and birch) as a developer. We observed a clear trend with increased C.D. for higher DFX loadings, reaching a plateau for the formulation with 56 wt % of DFX [C.D. (PA pine–21:35:56 wt % of DFX, 120°C) ≈ 0.65:0.9/1] (fig. S19). Both softwood (pine) and hardwood (birch) PA lignin formulations showed good performance compared to the formulation using a commercial sensitizer, while BPA and H ester exhibited undesired background coloration even at room temperature (fig. S14C and text S2.7). As the optimal performance was obtained for 56 wt % of DFX incorporated in the formulation, coatings were prepared for various developers including BPA and lignins with this formulation (Fig. 3C). The resulting color of the lignin-based formulations at room temperature was systematically lighter compared to those with benzalphtalide due to the developer concentrations being lowered by the increase in sensitizer content (Fig. 3C). The performance of the coatings with DFX, while slightly inferior to that of the commercial sensitizer, still demonstrated C.D. superior to 1 for all fractionated lignin formulations at 140°C, making the thermal paper formulation promising for commercialization [commercial C.D. (120°C) threshold = 1.1] (*32*–*34*). Control formulations containing DFX but no developer showed only minimal background coloration [Fig. 3C; C.D. (140°C) = 0.2], confirming that the developer is the principal participant in the color development reaction even for this previously unknown type of sensitizer.

### Stability testing of the coatings

Upon light exposure, there is a risk for thermal papers to show background discoloration and/or image fading, and thus commercial thermal paper manufacturers usually advise conserving paper rolls in a dark place (*77*). To check that our formulations were reasonably stable, samples coated with formulations containing either benzalphthalide or DFX as the sensitizer were exposed to ambient conditions, near a window for 6 months (figs. S20 and S21). Both cases exhibited good coating stability with only negligible change in C.D. for all formulations (C.D. change below 0.15 after 6 months).

### Thermal paper prototype

**Fig. 5.**
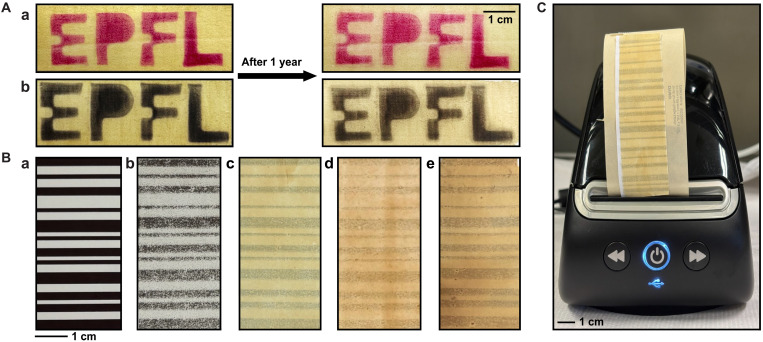
Lignin-based prototypes. (**A**) EPFL logo printed with thermal paper formulations containing IBA pine lignin developer and either (i) a red (Pergascript Red 16) or (ii) black (ODB-2) fluoran dye. The stability was observed by aging the paper for a year by a window. (**B**) Thermal printing in a commercial printer. Output comparison between (iii) commercial, (iv) in-house BPA control, (v) IBA pine, (vi) PA pine, and (vii) PA birch formulations, on paper (80 g m^−2^). (**C**) All samples were printed under identical conditions using a commercial thermal printer (DYMO LabelWriter 550).

The IBA pine lignin developer was selected to create a more realistic thermal paper prototype. Coatings were formulated using DFX as a sensitizer and the École Polytechnique Fédérale de Lausanne (EPFL) university logo was successfully printed using a poly(methyl methacrylate) mold and a thermal gun heater (Fig. 5A). Good contrast was observed, and the logo remained clearly visible after a year of storage, next to a window. The fading of the logos can be attributed to, e.g., the exposure of direct sunlight, which is known to limit the durability of thermal printouts (*78*). Last, we used a real printer to print bar codes and performed a side-by-side comparison of a commercial thermal paper (iii), an in-house BPA-based control formulation (iv), and our lignin-based formulations (v to vii) (Fig. 5, B and C). In all cases, we see a bar code appear, but the image contrast of all in-house formulated papers was not as good as the commercial paper. The similarity in image resolution between the in-house BPA and lignin formulations suggests that the limitation is primarily due to the paper coating and mixing/dispersion process rather than the developer chemistry itself.

### Toxicological assessment

The selected lignin fractions exhibited relatively high average molecular weights and contained only a limited fraction (0.04 to 2.3 wt %) of species below 500 Da (table S4 and figs. S22 and S23), a threshold typically associated with skin permeability. Nevertheless, a toxicity assessment was essential to ensure that the new formulations align with safety standards. We performed toxicity assessment on the individual formulation components (lignin-based developers and the DFX sensitizer) according to the known issues of both current developers (endocrine disruption) and sensitizers (environmental persistence and toxicity). Although this approach allowed us to better assess any toxicity contributions from individual components, future work should include testing the entire formulation and the leachate of our formulation versus off-the-shelf receipt thermal paper.

**Fig. 6.**
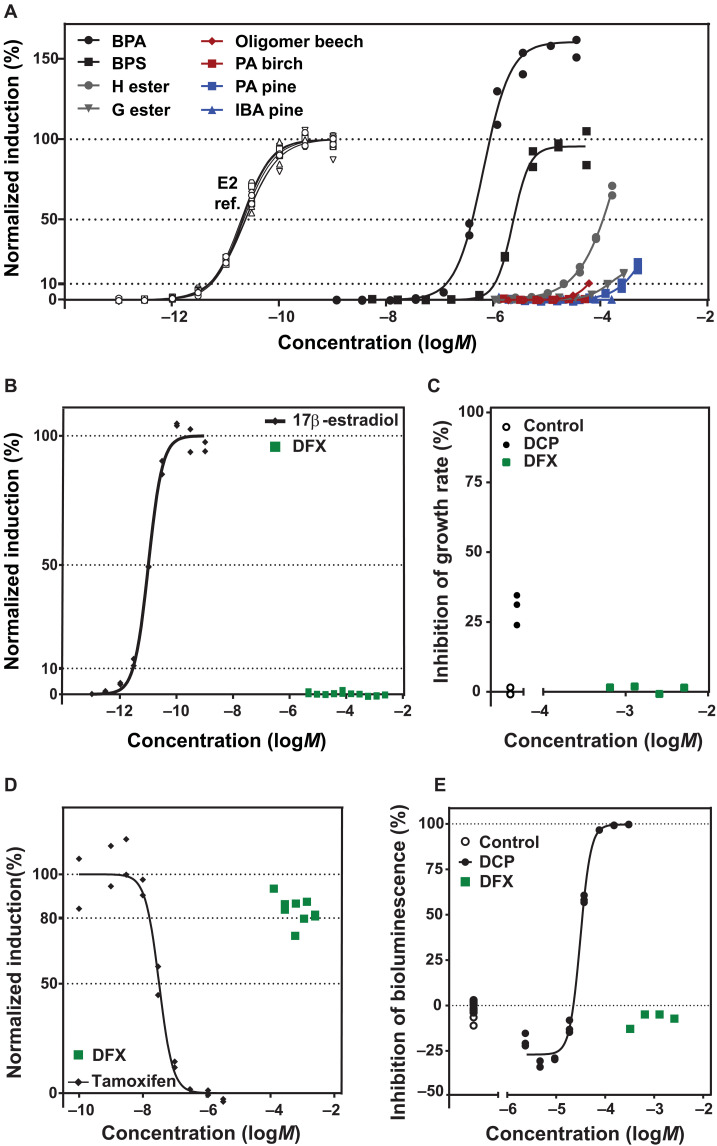
Toxicological assessment. (**A**) ERα-CALUX concentration-effect relationship (CER) of lignin-based developers compared to commercial BPA, BPS, and E2 reference (open symbols). Data were normalized to the effect of controls (0%) and the maximum modeled effect of E2 (100%). Curves on the left with open symbols are E2 reference data from the different plates on which the different compounds were tested. As seen elsewhere (*87*), BPA induces effects above that of the maximal induction caused by the positive control E2. This superinduction may not be biologically relevant but rather a result of stabilization of the luciferase reporter enzyme as has been described for genistein (*88*). (**B**) ERα-CALUX CER of DFX sensitizer compared to E2 reference. (**C**) Toxicity to freshwater algae as a positive control. (**D**) Normalized antagonistic ERα-CALUX estrogenic activity data of tamoxifen (reference) and DFX. (**E**) Bioluminiscence in bacteria using *A. fischeri* with DCP.

The potential endocrine activity of the best-performing lignin-derived color developers was compared to the natural hormone and positive control 17β-estradiol (E2) as well as commercial BPA and BPS in estrogen receptor α (ERα)– chemically activated luciferase gene expression (CALUX) and lyticase yeast estrogen screen (LYES) assays (text S2.10). In all cases, the lignin derivatives only showed agonistic estrogenic activity when at concentrations that were two to three orders of magnitude higher than BPS, which itself only induces a response at concentrations 10 times higher than BPA (Fig. 6A and figs. S24 to S28). Expectedly, lignin polymers induced a response only when at concentrations that were 10 times higher than the H ester. In addition, the normalized induction is significantly lower: 50% effective concentration (EC_50_) and 10% effect relative to positive control (PC10) for all phenolic compounds are listed in table S9 for ERα-CALUX and table S10 for LYES. We also used the ERα-CALUX assay to test antagonistic estrogenic activity using tamoxifen as the positive control. We did not observe antagonistic activity for any lignin developers, confirming the safe character of these bio-based developers in thermal papers (figs. S29 to S31). In addition, the same tests were also performed for the DFX sugar sensitizer and no agonistic (Fig. 6B and fig. S32) nor antagonistic (Fig. 6D) estrogenic activity was detected. For DFX, EC_50_ and PC10 are presented in tables S11 and S12.

To test for DFX’s more general toxicity profile, we used an algal growth assay and bioluminescent bacteria assay to assess possible toxicity to primary producers and decomposers. In the former, the positive control 3,5-dichlorophenol (DCP) inhibited growth by 30%, which is within the validity range of the Deutsche Institut für Normung (DIN) standard of 20 to 80%, while DFX did not inhibit algal growth at the tested concentrations (Fig. 6C). In the latter, the positive control DCP induced full inhibition of bioluminescence, and neither the blanks nor DFX caused inhibition of bioluminescence, at the tested concentrations (Fig. 6E). Together with its inherently biodegradable character (*62*), these initial toxicity data show strongly suggest that DFX is a benign replacement for traditional fossil-based and aromatic sensitizers.

## DISCUSSION

The combination of the formulation and toxicity results suggests a double benefit of functionalizing largely preserved natural structures to produce thermal paper formulations. First, the functionalization occurs during lignocellulosic biomass fractionation, leading to the simple and direct production of functionalized, yet structurally preserved, lignin and xylose. We introduced SAAF to produce lighter colored lignin in comparison to AAF. The economic implications of this sequential process should be further investigated through a technoeconomic analysis and life cycle assessment. Second, the preservation of these natural structures appears to largely limit toxicity. Sels and co-workers (*38*) had already demonstrated that the simple presence of methoxy substituents (which are ubiquitous in natural aromatics) markedly reduces the endocrine disruption of bisphenols. We now show that keeping the natural polymeric structure of lignin in the formulation further reduces this toxicity, as well as making dermal penetration much more challenging. Even if some toxicity were to occur that we have not tested for, skin permeability is likely to be very low, as only 0.04 to 2.3% of the lignin was determined to be below 500 Da. We also further demonstrate that having a sugar core in a polar aprotic molecule that facilitates good lignin developer-dye interactions substantially lowers toxicity, reinforcing our prior conclusions that had shown a lack of mutagenic potential for this molecule (*50*).

Overall, the proposed formulations not only provide a viable and promising pathway toward safer and bio-based thermal papers but also showcase the potential of functionalized and preserved natural structures for formulating cheap and safe renewable products for everyday use. While this study focused on static sensitivity, future work should also explore dynamic sensitivity to capture the rate of color formation, as this will have important implications for real-world printing performance.

## MATERIALS AND METHODS

### Materials

All chemicals were obtained from conventional vendors and were used as received from the supplier without additional purification. 1,4-Dioxane (≥99.5% for synthesis), paraformaldehyde (extra pure granules), sodium bicarbonate (NaHCO_3_), and 2-methyl tetrahydrofuran [>99%, SOLVAGREEN, stabilized with 250 parts per million of butylated hydroxytoluene (BHT)] were purchased from Carl Roth AG. 2-Chloro-4,4,5,5-tetramethyl-1,3-2-dioxaphospholane (95%), benzalphtalide (≥98%), BPA (99%), d-xylose (≥99%), IBA (≥99%), PVA (Mowiol 4-88; molecular weight ≈ 38,000 Da), potassium bromide (KBr; ≥99%), PA (97%), pyridine-d_5_ (≥99%), and zinc stearate were purchased from Sigma-Aldrich. ODB-2 and dihydroconiferyl alcohol (>98%) were provided by TCI Chemicals. 3-(4-Hydroxyphenyl)-1-propanol (97%), 3-(4-methoxyphenyl)-1-propanol (95%), 3-(4-methoxyphenyl)propionic acid (98%), CaCO_3_, chromium(III) acetylacetonate (97%), dihydro-*p*-coumaric acid (98%), dihydroferulic acid (98%), and GA monohydrate (97%) were obtained from Chemie-Brunschwig. Chloroform-d_1_ (99.8 atom % D) was purchased from Cambridge Isotope Laboratories. Diethyl ether (stabilized with BHT) was purchased from Carlo Erba Reagents. Ethanol, hydrochloric acid (37%, w/w, analytical reagent grade), and sulfuric acid (H_2_SO_4_; 95 to 97%, w/w) were obtained from Thermo Fisher Scientific. Ethyl acetate, hexanes, and methanol (≥99%) were purchased from Thommen-Furler AG. BPS (99%) and *N*-hydroxy-5-norbornene-2,3-dicarboximide were purchased from Alfa Aesar. Octadecanol (97%), diphenylsulfone (97%), and ruthenium on carbon (5%) were obtained from Strem Chemicals. *para*-Toluene sulfonic acid (99%) was purchased from Acros Organics. Pergafast Red 16 (95%) and toluene (99.8%; anhydrous, packaged under argon in resealable ChemSeal bottle) were purchased from abcr GmbH. Sodium hydroxide (NaOH; pellets) was purchased from Reactolab SA.

For the toxicological assessment, the origin of the biological materials are specified below. All chemicals (solvents and tested references) were obtained from conventional vendors and were used as received from the supplier without additional purification. DCP (97%), BPA (99%), dimethyl sulfoxide (DMSO; Hybri-Max, BioReagent; ≥99.7%), ethanol (≥99.9%; absolute for analysis EMPURE), and tamoxifen (≥99%) were purchased from Sigma-Aldrich. BPS (99%) was purchased from Alfa Aesar. E2 (100 μg ml^−1^ in acetonitrile) was purchased from LGC Standards. In ERα-CALUX, cells were obtained from Bio Detection Systems (Amsterdam, The Netherlands) and run under license. In LYES, cells were originally obtained from Brunel University (Uxbridge, Great Britain). In bioluminescence, freeze-dried bacteria were obtained from Dr. Lange (Düsseldorf, Germany). In algal growth, algae were obtained from the Culture Collection of Algae at the Georg-August-University Göttingen (Germany).The paper substrate for the coatings was commercially available printing paper–type PlanoJet (200 g m^−2^).

### Wood

Birch wood was procured from M. Studer of the Bern University of Applied Sciences. The birch tree (*Betula pendula*, ~40 years old) was harvested in May of 2018 in Solothurn, Switzerland. The tree was debarked, and the trunk was converted into wood chips and then air dried at 40°C for 24 hours. These wood chips were then collected and transported to EPFL where they were sieved and sorted to remove residual bark and leaves. The wood chips were then milled using a 6-mm screen and then machine sieved with a 0.45-mm mesh to remove fines.

Beech wood was procured from M. Studer of the Bern University of Applied Sciences. The same treatment as described above was performed. Pine wood was provided by P. Arnold (University of Lausanne, Switzerland). The same treatment as described above was performed.

### Color activity test

Octadecanol (100 mg) was introduced in a 5-ml glass vial (surface, 2.54 cm^2^), followed by the addition of developer [21 μmol = 1 phenol molar equivalent (eq.)] and leuco dye OBD-2 (10 mg, 21 μmol, 1 molar eq.). The phenolic molar ratio was kept constant for different developers. Without prior grinding, the powders were manually mixed for 30 s, and the mixtures were heated to 100°C, before being rapidly cooled down to room temperature. Last, the samples’ colorimetric properties were first measured using either the CIE L*a*b* or the CIE *Yxy* color spaces, using a colorimeter. CIE L*a*b* is a color space designed to match how humans see color differences, with separate components for lightness (L*) and color (a* for green-red and b* for blue-yellow). CIE *Yxy*, on the other hand, focuses on luminance (*Y*) and chromaticity (*x* and *y*), making it more suitable for color reproduction and lighting applications but less accurate for perceptual comparisons. The *Y* parameter relates to absolute luminance based on the physical measurements of light intensity and ranges from 0 (black) to 100 (white) and is directly related (although not directly proportional) to L* (text S1.6). The C.D. is calculated from the luminance *Y* parameter and represents the visual intensity and richness of a color, which, in turn, can be used to characterize contrast change on paper during the heating/printing process [C.D. = −log(*Y*/100)]. We calibrated this C.D. measurement with a near-black calibrant (C.D. = 2.5) and a near-white calibrant (C.D. = 0.09). When describing coloration, we converted the CIE *Yxy* parameters to the L*a*b* color space, the parameters of which are easier to interpret and thus are more commonly used to describe color. Specifically, the a* parameters represent the green (−100) to red (+100) color tone, and the b* parameters represent blue (−100) to yellow (+100) color tone (Fig. 1D and text S1.6). We also performed this test while keeping a constant mass ratio of developer to dye of 2:1 for different developers; the corresponding protocol can be found in text S2.6.

### Thermal paper formulation

All solid ingredients (developer, dye, CaCO_3_, zinc stearate, and sensitizer) were individually ground with a mortar for 5 min to decrease particle size to ~1000 nm (table S13) (*70*, *79*, *80*). Then, an aqueous solution of PVA with a solid content of 30 wt % was prepared by dissolving Mowiol 4-88 (molecular weight ≈ 38,000 Da, 300 mg) in distilled water (700 mg) overnight. In parallel, an aqueous CaCO_3_ solution was prepared by manually mixing CaCO_3_ (80 mg), PVA (30 wt %; 75 mg), and distilled water (150 mg) for 20 min until a homogeneous white solution was formed. Following these steps, the developer (50 mg) was mixed with the aqueous 30 wt % of PVA solution (80 mg), followed by addition of distilled water (152 mg), CaCO_3_ solution (300 mg), zinc stearate (20 mg), and benzalphtalide sensitizer (50 mg). This formulation was then mixed for another 5 min. The dye was incorporated (22 mg; which corresponded to a dye:developer mass ratio of 1:2), reaching a total solid content at 40 wt %. The final solution was applied to white paper (200 g m^−2^) using a manual U-coater (fig. S12), achieving a coating thickness within the range of 75 ± 5 μm and a coating of 75 ± 5 g m^−2^. A summary of all formulations and achieved C.D.s can be found in text S2.7 and tables S14 to S17. Furthermore, this commercial formulation was also performed with another petroleum-based sensitizer, diphenylsulfone, and the sugar-based sensitizer DFX (text S2.7). To assess the formulations’ static sensitivity, the coated papers were then heated using a temperature-controlled heat gun, positioned at a fixed height (1.5 cm). The heating was applied for 10 s once the heat gun had reached the target temperature, ensuring consistent thermal exposure across samples (text S2.7).

### Toxicological assessment

#### Estrogenic activity

Agonistic estrogenic activity of lignin-derived color developers and DFX was determined using two estrogen receptor reporter transactivation assays, both with the human estrogen receptor:LYES following International Organization for Standardization (ISO) 19040-1 (*81*) and a human cell line–based assay (ERα-CALUX) following ISO 19040-3 [for details, see Simon *et al.* (*82*)]. In both assays, E2 serves as the positive control. E2 and lignin-derived color developers were dissolved in DMSO and tested, in duplicate, in dilution series with a final DMSO concentration of 0.8% in all wells. A solution of 0.8% DMSO alone served as negative control. Tested concentration levels with observed cytotoxicity were excluded from the analysis and are reported in tables S9 to S12. Remaining data were fitted using Prism (8.0.1) to establish concentration-effect relationships (CERs) and determine EC_50_ as well as PC10 and PC50 values (see Organisation for Economic Cooperation and Development 455 and ISO 23196) (*83*, *84*). For antagonistic activity, we screened only using the ERα-CALUX. Except for negative control wells, E2 (1.49 × 10^−11^ M) was added to all wells of a plate to reach an estrogen receptor activation in the range of 50 to 70% of the plateau response of a full E2 CER. As a positive control for antagonistic effects, we used tamoxifen in DMSO tested in a dilution series, whereas DMSO in assay medium served as a negative control. Compounds in DMSO were also tested, in duplicate, in a dilution series. The final in-test DMSO concentration was again 0.8%.

#### Toxicity to bioluminescent bacteria

Given the known toxicity of past sensitizers, we investigated DFX’s toxicity to bacteria using the bioluminescent species *Aliivibrio fischeri* as described elsewhere (*85*). DCP was used as positive control and was tested in triplicate in a twofold dilution series over eight dilutions. DFX was dissolved in nanopure water and tested as unicate in a twofold dilution series over four dilutions. Nanopure water served as a negative control.

#### Toxicity to freshwater algae

We used DIN 38412-59 (*86*) to investigate DFX’s toxicity to *Raphidocelis subcaptitata*, a unicellular alga. The assay runs on 24-well plates using a volume of 2 ml. Assay medium controls provide the level of 100% growth (0% inhibition), DCP (2.1 mg liter^−1^) serves as positive control with a validity criteria inhibition range of 20 to 80%. DFX was dissolved directly into the assay medium and tested at four concentrations using a twofold dilution series starting at a maximum concentration of 0.9 g liter^−1^ or 5.2 × 10^−3^ M. All controls and samples were tested in triplicate. Algal growth was monitored every 24 hours over 3 days.

## Supplementary Materials

### This PDF file includes:

Supplementary Text S1 and S2 Figs. S1 to S33 Tables S1 to S20 List of abbreviations References
