# Supplementary material for: Sustainable thermal paper formulation using lignocellulosic biomass fractions
Source: Sci Adv. 2026 Jan 2;12(1):eadw9912. doi: 10.1126/sciadv.adw9912 (PMC12758521; doi:10.1126/sciadv.adw9912)
Supplement: Supplementary file 1 — Supplementary Text S1 and S2 Figs. S1 to S33 Tables S1 to S20 List of abbreviations References [file sciadv.adw9912_sm.pdf]

Science Advances
AAAS

Supplementary Materials for

Sustainable thermal paper formulation using lignocellulosic biomass fractions

Tom Nelis *et al.*

Corresponding author: Manon Rolland, manon.rolland@epfl.ch; Harm-Anton Klok, harm-anton.klok@epfl.ch; Jeremy S. Luterbacher, jeremy.luterbacher@epfl.ch

Sci. Adv. **12**, eadw9912 (2026)
DOI: 10.1126/sciadv.adw9912

This PDF file includes:

Supplementary Text S1 and S2
Figs. S1 to S33
Tables S1 to S20
List of abbreviations
References

Supplementary Text

S1 Supplementary Instruments and analytical methods

S1.1. Nuclear Magnetic Resonance (NMR).

^{1}H nuclear magnetic resonance (^{1}H-NMR) spectra were acquired using a Bruker Avance III 400 MHz spectrometer (T = 9.40 T) equipped with a $BBFO_z$ 5 mm probe using the standard pulse sequences "zg" from Bruker.

^{1}H-^{13}C Heteronuclear single quantum coherence spectroscopy (^{1}H-^{13}C HSQC) spectra were measured using a Bruker Avance 500 MHz spectrometer (11.75 T) with a 5 mm proton-optimized triple resonance NMR 'inverse' TCI cryoprobe to reduce electronic noise and enhance acquisition sensitivity.

Quantitative ^{31}P NMR (^{31}P NMR) spectroscopy was performed following a procedure published by Meng *et* al. (*67*) to measure the hydroxyl groups contents of extracted lignin samples. Briefly, lignin materials were dried overnight at 45 °C in a vacuum oven. Approximately, 30 mg of lignin were inserted in a glass vial equipped with a magnetic stir bar, closed with a septum cap and purged in a nitrogen atmosphere. We then added 0.1 mL of a solution of deuterated pyridine and $CDCl_3$ (1.6 : 1, *v*/*v*) containing a relaxation agent, chromium(III) acetylacetonate solution (≈ 5.0 $mg.mL^{-1}$) and N-hydroxy-5-norbornene-2,3-dicarboximide (NHND) as an internal standard (≈ 18.0 $mg.mL^{-1}$) with a gas-tight syringe. After complete solubilization of the material within 0.5 mL of a solution of deuterated pyridine and $CDCl_3$ (1.6 : 1, *v*/*v*), 0.1 mL of the phosphorylating agent, 2-chloro-4,4,5,5-tetramethyl-1,3-2-dioxaphospholane (TMDP), stored under inert atmosphere, was added dropwise to the solution. The mixture was stirred for an additional hour to achieve complete derivatization and transferred to an NMR tube purged under nitrogen and equipped with a rubber septum. Due to limited stability of the derivatization, samples were analyzed within 3 h of their preparation, on a Bruker Avance 600 MHz spectrometer equipped with a 5 mm BBO cryoprobe. The experimental parameters used for the spectra acquisition were: pulse program=inverse gated decoupling pulse (zgig), SW=100 ppm, O1P=140 ppm, AQ=0.8 s, D1=10 s, NS=128.

S1.2. Thermal Analysis.

Thermogravimetrical Analysis (TGA)

5-10 mg of dried product was analyzed using the following program: Initial temperature: 30.00 °C, Switch the Gas to Air at 20.0 $ml.min^{-1}$.

1) Hold for 5.0 min at 30.00 °C
2) Heat from 30.00 °C to 600.00 °C at 10.00 $°C.min^{-1}$
3) Hold for 1.0 min at 600.00 °C

Degradation onset temperature indicates the temperature at which a 5% mass loss was observed and will be noted $T_{d,95\%}$.

S1.2. Thermal Analysis (continued).
Differential Scanning Calorimetry (DSC)
DSC for all powdered color developer samples were measured under a nitrogen atmosphere. Between 5-10 mg of sample were used for each analysis and the following DSC program was used:

1. Ramp 5.00 °C/min to 170.00 °C (Scan 1)
2. Ramp 10.00 °C/min to 0.00 °C
3. Ramp 5.00 °C/min to 170.00 °C (Scan 2)

For the small molecules (BPA and H ester), the first scan was used whereas for oligomeric and polymeric lignin the second scan was used after the first scan was used to eliminate any thermal history (fig. S11).

S1.3. Dynamic light scattering (DLS).
DLS measurements were performed using Malvern Zetasizer. The sample refractive index (RI) was set to 1.59. The dispersant viscosity was set to 0.89 $Ns.m^{-2}$. To determine particle size, all measurements were diluted 10 times.

S1.4. Fourier Transform Infrared spectroscopy (FT-IR).
FT-IR spectra were acquired on a Perkin ElmerSpectrum Frontier 3 FT-IR Spectrometer instrument, with a liquid nitrogen-cooled mercury cadmium telluride detector, over a range of 4000 – 400 cm^{-1}, using on average 64 scans. Prior to acquisition, lignin materials were dried on a Schlenk line over the weekend. 20 mg of lignin material was mixed with 100 mg of KBr and ground for 5-10 min by hand, until a fine and homogeneous powder was obtained. For the blank experiment, only KBr was used.

S1.5. Size Exclusion Chromatography (SEC).
Polymer molecular weight distributions were analysed qualitatively using an Agilent 1260 Infinity (HPLC) coupled with 390-MDS detectors and equipped with two columns: a PL gel 5 µm Guard, 7.5 x 50 mm and a SDV 100 Å, 8 x 300 mm, 3 µm. The calibration was performed with polystyrene standards, between 162 and 17780 Da. Samples were prepared in THF with an approximate concentration of 3 - 5 $mg.mL^{-1}$. Runs were performed at 25 °C with a flow of 0.6 $mL.min^{-1}$. The RI detector sensibility to molecular weight was assessed by injecting three known masses of polystyrene standards and comparing their respective area per mole (values tabulated below). This analysis revealed that a linear correlation can slightly underestimate (< 25 %) the fraction of low molecular weight species.

Gel Permeation Chromatography (GPC) peak areas obtained from injections of polystyrene standards of varying molecular weights (Mp), showing corresponding mass injected (mg), amount in mmoles, calculated surface area (mm^2), and the ratio of area to mass (A/m) (m^2/mg).

Mp	mg	mmole	Area (mm^2)	Ratio A/m
**162**	2.8	17.3	44.1	1.57 E-05
**1790**	2.9	1.62	57.2	1.97 E-05
**7190**	2.8	0.389	58.9	2.10 E-05

S1.6. Colorimeter.
The color changes were monitored using a colorimeter (CS-10 Colorimeter, CHNSpec, China).

The two most widely used methods for expressing colors numerically were introduced by the *Commission Internationale de l'Éclairage* (CIE): The *Yxy* color space, followed later by the $L^*a^*b^*$ color space (fig. 1D). The latter was introduced to provide more perceptually uniform color differences, addressing a major limitation of the original *Yxy* system – namely that equal distances in the (x,y) chromatography diagram did not correspond to equal perceived color differences.(*89,90*)

For the CIE $L^*a^*b^*$ space:
- The L^* value represents perceptual lightness, which is how humans perceive lightness differences, with 0 being the darkest black and 100 being the brightest white.
- The a^* axis represents the red-green color spectrum, with negative values towards green shades (absolute green a* = -100) and positive values towards red ones (absolute red a* = +100).
- The b^* axis represents the yellow-blue color spectrum, with negative values towards blue shades (absolute blue b* = -100) and positive values towards yellow ones (absolute yellow b* = +100).

For the CIE *Yxy* space:
- The *Y* parameter relates to absolute luminance based on the physical measurements of light intensity and ranges from 0 (black) to 100 (white).
- The *x* and *y* are the chromaticity coordinates that define the color hue and saturation. They both range from 0 to 1, and together describe the color in the chromaticity diagram. x ranges from 0 to 1 (this axis corresponds to the red-green component) and y ranges from 0 to 1 (this axis corresponds to the yellow-blue component).

The CIE $L^*a^*b^*$ space is a uniform color space designed to match how humans see color differences, with separate components for lightness (L^*) and color (a^*, b^*, fig 1D). CIE *Yxy*, on the other hand, focuses on luminance (*Y*) and chromaticity (*x, y*), making it more suitable for color reproduction and lighting applications but less accurate for perceptual comparisons.(*89,90*)

The colorimetric properties of our samples were measured using both CIE $L^*a^*b^*$ and *Yxy* color spaces (tables S2, S6, S7, S14-S17). To quantify color development in the color activity tests and real formulations, we calculated color density (C.D.) from the luminance parameter *Y* of the traditional CIE *Yxy* space, which is still used by the industry for characterization of color development performance:

$$Color\ Density\ (C.D) = -\log\left(\frac{Y}{100}\right)$$

Y (from the *Yxy* space) is directly related (though not directly proportional) to L^*(from the $L^*a^*b^*$ space) and they can be calculated from one another via the following formula:

$$L^* = 116\left(\frac{Y}{Y_n}\right)^{1/3} - 16$$

With *Y* being the luminance of the specimen, and Y_n the luminance of the reference (perfect reflecting diffuser, usually measured with a white calibrant).(*89,90*) This relation leads to both L^*

and *Y* being close to zero and close to 100 for a near black and near white value, respectively. However, it is non linear and so they will deviate from each other between these two values, with *L** values around *50* leading to *Y* values slightly below 20, etc.

S2. Supplementary Experimental methods

S2.1. Aldehyde Assisted Fractionation (AAF).

Lignin was extracted following the Aldehyde Assisted Fractionation (AAF) technology developed by our group.(*46,47*) Using aldehydes during a modified organosolv pretreatement, we can stabilize the β-O-4 linkages via the formation of acetal structure on the diol, yielding native-like and uncondensed lignins. Approximately 9 g of birch or pine wood chips (250-650 μm), 6.6 equivalent of aldehyde (9.6 ml of propionaldehyde (PA), 9.0 mL of isobutyraldehyde (IBA) or 4.9 g of glyoxylic acid monohydrate (GA)), 1 equivalent of HCl 37 wt.% in water (1.7 mL), and 50 mL of dioxane were introduced in a 250 mL round-bottomed flask along with a 30 mm long magnetic stirrer. After fitting a condenser equipped with a bubbler onto the flask, the solution was heated at 85 °C in an oil bath. After 3 h of reaction, the mixture was cooled down to room temperature and then filtered with filter paper to remove the cellulose-rich solids. Three dioxane-washes successfully removed all soluble compounds. The filtrate, containing the lignin, hemicellulose, and excess aldehyde, was then concentrated on a rotary evaporator at 55 °C at reduced pressure (70 mbar). The concentrated liquor obtained was then diluted with 20 mL of ethyl acetate and added dropwise to 500mL of hexanes (or water with GA protected lignin) while vigorously stirring (650 rpm) to precipitate the lignin as a fine powder. Finally, the solution was filtered on a 0.8 μm Nylon filter to extract the aldehyde-protected lignin. The recovered powder was then mixed with 50 mL of diethyl ether and sonicated for 20min, before being centrifuged for 10 min at 3,480 rpm. Washed lignin was then dried overnight in a vacuum oven at 45 °C. Birch (hardwood) lignins were recovered as purple materials, while Pine (softwood) lignins were recovered as a light grey powder.

S2.2. Sequential Aldehyde Assisted Fractionation (SAAF).

To meet thermal paper commercialization requirements, color density (C.D.) contrast has to be maximized. For this purpose, it was necessary to extract light-colored lignin from birchwood. The AAF process was therefore applied sequentially on a wood sample. The first extraction was run for 30 min, after which a first batch of dark-purple lignin was extracted. The filtered cellulose-rich solids were then collected and re-extracted using reduced volumes of fresh dioxane, aldehyde and acid based on the lignin mass primarily extracted. After four extraction batches (namely after 30min, 1h, 2h and 3h total extraction time), an important decrease of lignin color was observed in the resulting solids, evolving from dark purple to light grey (fig. S3B, example given for Birch wood and PA aldehyde). Extraction yields per cycle are reported in Tables S18-S20.

No structural differences were observed between those batches when studied with ^{1}H-^{13}C HSQC. However, we noticed the extraction of longer lignin polymers with increasing extraction time, revealed by size exclusion chromatography (SEC) (table S3). This correlated with the measurement of the phenolic content via ^{31}P NMR (table S5). Indeed, free-phenolic groups are mostly found as chain-end within largely linear lignin oligomers. Shorter oligomers therefore present higher phenolic content per gram of materials. Higher carbonyl content was also measured via FTIR on those early-extracted materials (table S3). Within the lignin backbone, conjugated carbonyl groups and phenolics are known to absorb visible light, therefore inducing lignin dark color.(*55,91*) Presenting higher content of both functional groups, an early extracted lignin batch is therefore highly colored.

S2.3. Lignin oligomers extraction.

Oligomers Beech. Lignin was extracted from 25 kg of beech wood using glyoxylic acid in 2-methyl-THF. 1 kg of extracted lignin was then depolymerized in a 10 Liter-PARR reactor with ethanol solvent under hydrogen pressure using Ru/C as a catalyst. After depolymerization, the resulting oil was concentrated under reduced pressure to remove ethanol (50 °C, 20 mbar). A distillation column was then used up to 210 °C and 0.2 mbar to distil the monomers and recover the oligomers remaining in the flask.

Oligomers Pine. Lignin was extracted from 800 g of black pine wood using IBA in 2-methyl-THF. The extracted lignin was then depolymerized in a 1 L-PARR reactor with ethanol solvent under hydrogen pressure using Ni/C as a catalyst. The resulting oil was concentrated under reduced pressure to remove ethanol (50 °C, 20 mbar). The concentrated lignin oil was stirred with hexanes at reflux to extract the monomers. The oligomer-rich residue was then dissolved in methanol and added dropwise into water in order to precipitate the oligomers, that were recovered as a powder by filtration and subsequent drying (0.2 mbar, 45 °C).

S2.4. Preparation of lignin-derived esters.

H ester. Dihydro-*p*-coumaric acid (1 g, 6.02 mmol, 1.00 eq.) was combined with 3-(4-hydroxyphenyl)-1-propanol (0.92 g, 6.02 mmol, 1.00 eq.), *p*-toluenesulfonic acid monohydrate (57 mg, 0.3 mmol, 0.05 eq.) and toluene (60 mL) in a 3-neck 100 mL round-bottom flask of a Dean-Stark set-up. The mixture was heated to 120 °C using an oil bath under N_2-flow and with magnetic stirring (400 rpm using a PTFE coated stir-bar) for 6 h. The reaction progress was followed by Thin Layer Chromatography (50 : 50 hexane : ethyl acetate). After full conversion, the reaction was stopped and the solvent was removed under reduced pressure. The crude was then diluted with dichloromethane, washed 1x with 0.1 M $NaHCO_3$ solution, 1x demineralized water and 1x brine. The organic phase was then concentrated under reduced pressure to yield the H ester product as an off-white solid (fig. 1D) (96 % isolated yield). The purity (> 98 wt.%) of the final product was confirmed by quantitative ^{1}H NMR with 1,2,4,5-terachloro-3-nitrobenzene as the internal standard.

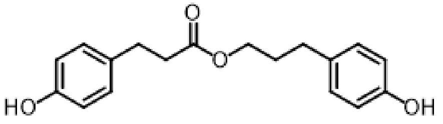

3-(4-hydroxyphenyl)propyl 3-(4-hydroxyphenyl)propanoate
H Ester

^{1}H NMR (400 MHz, DMSO-*d*$_6$)
δ 1.82 (2H, quint), 2.51 (2H, t), 2.59 (2H, t), 2.78 (2H, t), 4.00 (2H, t), 6.71(4H, d), 6.99 (2H, d), 7.05 (2H, d), 9.18 (1H, s), 9.21 (1H, s).

^{13}C NMR (101 MHz, DMSO-*d*$_6$)
δ 172.83 (1C, s), 156.06 (1C, s), 155.86 (1C, s), 129.55 (4C, d), 123.56 (1C, s), 115,51 (4C, d), 63.60 (1C, 1s), 55.94 (1C, s), 35.97 (1C, s), 30.98 (1C, s), 30.49 (1C,s), 30.04 (1C, s).

HRMS (ESI/QTOF)
m/z: $[M + Na]^+$ Calculated for $C_{18}H_{20}NaO_4$+ 323.1254; Found 323.1261.

S2.4. Preparation of lignin-derived esters (continued).

G ester. Dihydroferulic acid (1 g, 5.10 mmol, 1.00 eq.) was combined with dihydroconiferyl alcohol (0.93 g, 5.10 mmol, 1.00 eq.), *p*-toluenesulfonic acid monohydrate (48 mg, 0.25 mmol, 0.05 eq.) and toluene (60 mL) in a 3-neck 100 mL round-bottom flask of a Dean-Stark set-up. The mixture was heated to 120 °C using an oil bath under N_2-flow and with magnetic stirring (400 rpm using a PTFE coated stir-bar) for 6 h. The reaction progress was followed by Thin Layer Chromatography (50 : 50 hexane : ethyl acetate). After full conversion, the reaction was stopped and the solvent was removed under reduced pressure. The crude was then diluted with dichloromethane, washed 1x with 0.1 M $NaHCO_3$ solution, 1x demineralized water and 1x brine. The organic phase was then concentrated under reduced pressure to yield the G ester product as a viscous yellowish oil (90 % isolated yield). The purity of the solid was determined by ^{1}H-NMR. To remove trace impurities, the oil was purified by Silica-gel Flash Column Chromatography, yielding the G ester as a transparent viscous oil (fig. 1D). The purity (> 98 wt.%) of the final product was confirmed by quantitative ^{1}H NMR with 1,2,4,5-terachloro-3-nitrobenzene as the internal standard.

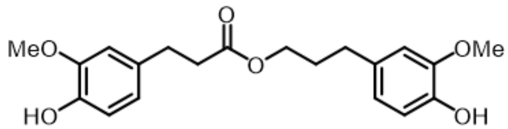

3-(4-hydroxy-3-methoxyphenyl)propyl 3-(4-hydroxy-3-methoxyphenyl)propanoate

G Ester

^{1}H NMR (400 MHz, DMSO-*d*$_6$)

δ 1.82 (2H, quint), 2.50(2H, t), 2.59 (2H, t), 2.77 (2H, t), 3.75 (6H, s), 3.99 (2H, t), 6.56 (1H, d), 6.61 (1H, d), 6.69 (2H, d), 6.74 (1H, s), 6.79 (1H, s)8.69 (1H, s), 8.71 (1H, s).

^{13}C NMR (101 MHz, DMSO-*d*$_6$)

δ 172.83 (1C, s), 147.89 (1C, s), 147.87 (1C, s), 145.26 (1C, s), 145.05 (1C, s), 132.34 (1C, s), 131.75 (1C, 1s), 120.81 (1C,s) , 120.73 (1C,s), 115,78 (1C, s), 115.75 (1C, s), 112.87 (2C, s), 63.67 (1C, 1s), 55.94 (2C, s), 35.97 (1C, s), 31.45 (1C, s), 30.49 (1C,s), 30.46 (1C, s).

HRMS (ESI/QTOF)

m/z: $[M + Na]^+$ Calculated for $C_{20}H_{24}NaO_6$+ 383.1465; Found 383.1468.

S2.5. Preparation of Diformylxylose (DFX)

DFX. The protocol was adapted from Komarova *et al.* (*50*). D-Xylose (30 g, 200 mmol, 1.00 eq.) was combined with paraformaldehyde (24 g, 800 mmol, 4 eq. of formaldehyde) and 2-Methyltetrahydrofuran (300 mL) in a 1000 mL round bottom flask. Next, sulfuric acid (31.39 g, 320 mmol, 1.60 eq.) was dissolved in 2-Methyltetrahydrofuran (100 mL) and the mixture was added dropwise to the xylose-solution while stirring vigorously. The mixture was then heated to 80 °C for 30 min while stirring. After reaction, the solution was cooled to room temperature, neutralized with NaOH, filtered, and concentrated *in vacuo* using a rotary evaporator with a bath temperature of 45 °C. The residue (yellow oil) was put in the fridge overnight and seeded with an earlier obtained DFX crystal to initiate crystallization. The obtained crystals were washed with cold ethanol and dried at 0.02 mbar to yield pure white DFX crystals (24.36 g, 70 % isolated yield). The purity (> 99 wt.%) of the final product was confirmed by quantitative ^{1}H NMR with 1,2,4,5-terachloro-3-nitrobenzene as the internal standard.

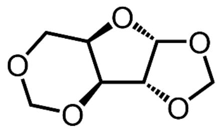

Diformyl xylose (DFX)

^{1}H NMR (400 MHz, DMSO-d_6)
δ 3.84 (1H, d), 3.92 (1H, d), 4.01 (1H, d), 4.29 (1H, d), 4.37 (1H, d), 4.62 (1H, d), 4.83 (1H, d), 4.97 (2H, d), 5.97 (1H, d).

^{13}C NMR (101 MHz, DMSO-d_6)
δ 104.74 (1C, s), 96.28 (1C, s), 91.04 (1C, s), 83.48 (1C, s), 77.33 (1C, s), 75.24 (1C, s), 65.54(1C, s).

S2.6. Formulations for color activity test (octadecanol) with mass ratio constant. Octadecanol (100 mg) was introduced in a 5 mL glass vial, followed by the addition of developer (20 mg) and leuco dye OBD-2 (10 mg). The powders were manually mixed for 30 s and C.D. was measured using a colorimeter. Then, the mixtures were heated to 100 °C and cooled down to room temperature. Finally, C.D. was remeasured to quantify the color change**.**

S2.7. Formulations for Thermal Paper Coatings.
Raw data regarding color density of all formulations can be found in Tables S14-17.

Thermal paper formulation 1. Without sensitizer. First, the solid ingredients (developer and dye) were individually ground in a separate mortar for 5 min to decrease particle size to approximately 1000 µm. Then, an aqueous solution of poly vinyl alcohol (PVA) with a solid content of 30 wt.% was prepared by dissolving Mowiol(R) 4-88 (300 mg) in distilled water (700 mg) overnight. Following these steps, the developer (60 mg) was mixed with the aqueous 30 wt.% PVA solution (60 mg), followed by the addition of water (200 mg). The formulation was, then, mixed for another 5 min. Finally, the dye was incorporated (30 mg, dye:developer mass ratio 1:2) and the total solid content was maintained to 40 %. The final solution was applied to white paper using a U-coater (fig. S12), achieving a coating thickness within the range of 75 +/- 5µm and a coating of 75 +/-5 gram per square meter (GSM). The formulation total mass, dry mass percentages, and wet mass percentages are summarized in Formulation S1.

Formulation S1. With total mass, dry mass percentage and wet mass percentage while using no sensitizer and varying the developer. Solid content = 43 %.

Component	Mass (mg)	Dry Mass (%)	Wet Mass (%)
**PVA (Mowiol(R) 4-88- M_n=38,000 Da)**	75	55	24
**Dye (ODB-2)**	20	25	6
**Developer**	40	30	13
**Water**	175	0	57
**Total Mass**	310	**100**	**100**
**Total Dry Mass**	135		

Thermal paper formulation 2. **With petroleum-based sensitizer benzalphtalide**. The procedure is described in the main text. Nevertheless, we have summarized the formulation total mass, dry mass percentages, and wet mass percentages in Formulation S2.

Formulation S2. With total mass, dry mass percentage and wet mass percentage while using benzalphtalide as sensitizer and varying the developer. Solid content = 40 %.

Component	Mass (mg)	Dry Mass (%)	Wet Mass (%)
**PVA (Mowiol(R) 4-88- M_n=38,000 Da)**	47	17	7
**Dye (ODB-2)**	22	8	3
**Developer**	50	19	7
**Water**	411	0	60
**$CaCO_3$**	80	30	12
**Zinc Stearate**	20	7	3
**Sensitizer (benzalphtalide)**	50	19	7
**Total Mass**	679	**100**	**100**
**Total Dry Mass**	269		

S2.7. Formulations for Thermal Paper Coatings (continued).
Raw data regarding color density of all formulations can be found in Tables S14-17.

Thermal paper formulation 3. **With petroleum-based sensitizer diphenylsulfone**. First, the solid ingredients (developer, dye, $CaCO_3$, zinc stearate and sensitizer) were individually ground with a mortar for 5 min to decrease particle size to approximately 1000 µm. Then, an aqueous solution of poly vinyl alcohol (PVA) with a solid content of 30 wt.% was prepared by dissolving Mowiol(R) 4-88 (300 mg) in distilled water (700 mg) overnight. In parallel, an aqueous $CaCO_3$ solution was prepared by manually mixing $CaCO_3$ (80 mg), PVA 30 wt.% (75 mg) and distilled water (150 mg) for 20 minutes until a homogeneous white solution was formed. Following these preparation steps, developer (60 mg) was mixed with the aqueous 30 wt.% PVA solution (60 mg), followed by the addition of water (200 mg), $CaCO_3$ solution (200 mg), zinc stearate (10 mg) and sensitizer diphenylsulfone (50 mg) The formulation was, then, mixed for another 5 min. Finally, the dye was incorporated (30 mg, dye:developer mass ratio 1:2) and the total solid content was maintained to 40 % as described in formulation S3. The final solution was applied to white paper using a U-coater (fig. S12), achieving a coating thickness within the range of 75 +/- 5µm and a coating of 75 +/-5 gram per square meter (GSM).

Formulation S3. With total mass, dry mass percentage and wet mass percentage while using diphenylsulfone as sensitizer and varying the developer. Solid content = 40 %.

	**Mass (mg)**	**Dry Mass (%)**	**Wet Mass (%)**
**PVA (Mowiol(R) 4-88- M_n=38,000 Da)**	33	15	6
**Dye (ODB-2)**	30	13	5
**Developer**	60	27	11
**Water**	332	0	60
**$CaCO_3$**	40	18	7
**Zinc Stearate**	10	4	2
**Sensitizer (Diphenyl sulfone)**	50	22	9
**Total Mass**	**556**	**100**	**100**
**Total Dry Mass**	**223**		

S2.7. Formulations for Thermal Paper Coatings (continued).
Raw data regarding color density of all formulations can be found in tables S14-17.

Thermal paper formulation 4. With DFX sensitizer (56, 35, 21 dry mass % - 30,15, 8 wet mass %). First, the solid ingredients (developer, dye, $CaCO_3$, zinc stearate and sensitizer) were individually ground with a mortar for 5 min to decrease particle size to approximately 1000 µm. Then, an aqueous solution of poly vinyl alcohol (PVA) with a solid content of 30 wt.% was prepared by dissolving Mowiol(R) 4-88 (300 mg) in distilled water (700 mg) overnight. In parallel, an aqueous $CaCO_3$ solution was prepared by manually mixing $CaCO_3$ (80 mg, 25 wt %), PVA 30 wt.% (75 mg) and distilled water (150 mg) for 20 minutes until a homogeneous white solution was formed. Following these preparation steps, developer (60 mg, 8 wt.%) was mixed with the aqueous 30 wt.% PVA solution (60 mg), followed by the addition of distilled water (200 mg), $CaCO_3$ solution (200 mg, 13 wt %), zinc stearate (10 mg, 1 wt %), and sensitizer DFX (240 mg, 56 wt.%). The formulation was then mixed for another 5 min. Finally, the dye was gently incorporated (30 mg, dye : developer mass ratio, 1 : 2) and the total solid content was maintained to 53 wt.%. The final solution was applied to white paper using a U-coater (fig. S12), achieving a coating thickness within the range of 75 +/- 5µm and a coating of 75 +/-5 gram per square meter (GSM). The same procedure was realized by changing DFX loading to 35 and 21 dry mass percent leading to formulation with 43 and 38 % of total solid content, respectively, as described in Formulation S5 and S6.

Formulation S4. With total mass, dry mass percentage and wet mass percentage while using 56 dry weight % DFX as sensitizer and varying the developer. Solid content = 53 %.

	**Mass (mg)**	**Dry Mass (%)**	**Wet Mass (%)**
**PVA (Mowiol(R) 4-88- M_n=38,000 Da)**	33	8	4
**Dye (ODB-2)**	30	7	4
**Developer**	60	14	7
**Water**	377	0	47
**$CaCO_3$**	53	12	7
**Zinc Stearate**	10	2	1
**Sensitizer (DFX)**	240	56	30
**Total mass**	**803**	**100**	**100**
**Total Dry mass**	**426**		

For conventional color developers, like BPA, or the H ester, DFX was found to cause unwanted background coloration at room temperature. This issue was effectively addressed by tuning the aldehyde side chain of the acetal-stabilized sugar sensitizer—specifically, by extending the carbon chain length of the acetal groups. This modification influences the polarity of the formulation which likely plays a role modulating the color development reaction. Among the tested formulations, dibutyl xylose (DBX) and dipropyl xylose (DPX) emerged as a particularly effective sensitizers, yielding well-defined thermal response curves for BPA and IBA lignin as shown on fig. S15.

S2.7. Formulations for Thermal Paper Coatings (continued).
Raw data regarding color density of all formulations can be found in Tables S14-17.

Thermal paper formulation 5. With total mass, dry mass percentage and wet mass percentage while using 35 dry mass % DFX as sensitizer and varying the developer. Solid content = 43 %.

	**Mass (mg)**	**Dry Mass (%)**	**Wet Mass (%)**
**PVA (Mowiol(R) 4-88- M_n=38,000 Da)**	33	12	5
**Dye (ODB-2)**	30	10	5
**Developer**	60	21	9
**Water**	377	0	57
**$CaCO_3$**	53	19	8
**Zinc Stearate**	10	3	2
**Sensitizer (DFX)**	100	35	15
**Total mass**	**663**	**100**	**100**
**Total Dry mass**	**286**		

Thermal paper formulation 6. With total mass, dry mass percentage and wet mass percentage while using 21 dry mass % DFX as sensitizer and varying the developer. Solid content = 38 %.

	**Mass (mg)**	**Dry Mass (%)**	**Wet Mass (%)**
**PVA (Mowiol(R) 4-88- M_n=38,000 Da)**	33	14	5
**Dye (ODB-2)**	30	13	5
**Developer**	60	25	10
**Water**	377	0	62
**$CaCO_3$**	53	22	9
**Zinc Stearate**	10	4	2
**Sensitizer (DFX)**	50	21	8
**Total mass**	**613**	**100**	**100**
**Total Dry mass**	**236**		

S2.7. Formulations for Thermal Paper Coatings (continued).

To assess the thermal response of the thermal paper samples, the surface temperature of the paper was monitored using a Fluke Ti480 thermal imager. A temperature-controlled Makita HG6531CK heat gun was used for heating. Before each experiment, the heat-gun was first allowed to reach the desired setpoint temperature. The gun was then positioned at a fixed height of 1.5 cm above the paper surface, a distance maintained consistently across all tests (fig. S33A).

Heating was then applied for 10 seconds while the surface temperature of the paper was recorded. Thermal imaging confirmed that within this 10-second heating period, the paper surface reached the target temperature set on the heat gun. While some fluctuations were observed in the thermal readings, the average surface temperature closely matched the heat gun's setpoint, with a deviation of less than 2% (in °C) (fig. S33B)."

The pictures of the developed thermal papers for various heating temperatures were captured using an iPhone under ambient lighting conditions without any post-processing to reflect the actual visual appearance of the coatings.

S2.8. Thermal coating stability.

Coatings were applied following the above-described formulation procedures with benzalphtalide (S2.7. Formulation S2) or DFX (S2.7. Formulation S4) as sensitizers. Then the resulting papers were put next to a window for six months of natural light exposure (fig. S21). C.D values were periodically measured using a colorimeter. In addition, a picture of the EPFL logo was taken after one year under similar light conditions to visually evaluate the quality of the printing over time.

S 2.9. Microscopy analysis.

Formulation dispersity for the color activity (octadecanol) test and the coated paper were assessed using an optical microscope (Nikon Eclipse TS100).

S2.10. Brief methods for the assessment of toxicity.

Estrogenic effects using ERα-Chemical Activated LUciferase gene eXpression (CALUX). ERα-CALUX was performed on 96-well plates along ISO 19040-3 (2018).(*82,92*) Compounds were dissolved in DMSO and tested, in duplicate, in two-fold dilution over ten dilution steps. A dilution series of 17β-estradiol (E2) served as a reference compound (positive control). Possible cytotoxicity to the mammalian cells was verified under the microscope. All compounds showed toxicity at higher concentrations except for DFX. DFX was not toxic at the highest tested concentration of 2.4 10^{-4} M (figs. S24, S25, 6A, 6B and tables S9 and S12).

Incomplete concentration-effect relationships (CER) or CER with superinduction poses challenges for a robust evaluation of EC50 values. For this reason, and also to be in line with OECD guidance on detecting estrogen receptor agonists and antagonists (OECD 455, 2015)(*83*) and working along ISO guidelines (ISO 23196, 2022)(*84*), we reported PC10 and PC50 values for the tested compounds (tables S9-S12). OECD 455 also suggest testing compounds for antagonistic effects (*vide infra*).

Visual inspection of solubility of compounds tested in ERα-CALUX. Following addition of some of the compounds dissolved in DMSO (10 or 12.5 µL) to ERα-CALUX assay medium (1.2 or 1.5 mL) on 24-well plates, precipitation was observed for the three or four highest tested concentrations of: PA Birch, IBA Pine, Oligomers Beech and PA Pine (not for the other tested compounds). Following mixing of all wells, suspensions were obtained (figs. S27 and S28). These suspensions were pipetted to ERα-CALUX assay plates for testing.

Anti-estrogenic effects using ERα-CALUX. To explore possible anti-estrogenic effects and following OECD TG 455 (2015)(*83*), we adapted ISO 19040-3 (2018)(*92*) in two ways: 1) all wells on a 96-well plate received a concentration of 1.49 10^{-11} M E2 to target an assay induction slightly above 50 % effect of a full E2 concentration effect relationship; 2) a dilution series of the drug tamoxifen (3.3 10^{-6} to 1.0 10^{-11} M), in duplicate, was used as a positive control to evaluate the effectiveness of blocking the E2 mediated assay induction; 3) on each plate, two wells served as a spike control (on three plates 18 or 20 additional wells served as E2 spike control to evaluate E2 spike variability). Raw luminescent data were normalized by: fixing the top of the tamoxifen curve to the two E2 spike controls (i.e. 100%) and fitting the tamoxifen curve to determine the bottom (0 %). When a compound reduced induction by 20%, antagonistic activity was established. In case a compound only has agonistic estrogenic activity, induction over 100% was expected (e.g. BPA) (figs. S29-S31 and 6D).

S 2.10. Brief methods for the assessment of toxicity (continued).

Inhibition of bacterial bioluminescence. The assay used the marine bacterium *Allivibrio fischeri* on 96-well plates and is based on Escher *et al*. (*85*), see also Vermeirssen *et al*. (*93*). In the assay, toxic compounds may reduce the naturally occurring luminescence in these bacteria. The assay used a dilution series of 3,5-dichlorophenol (DCP) as positive control.

DFX dissolved in water to a concentration of 1 $g.L^{-1}$ was tested in the screening using a two-fold dilution series of DFX over four steps and testing concentrations in unicate. As no toxicity was indicated at the tested concentrations (fig. 6E), no further analyses were performed.

Inhibition of algal growth. The three-day algae test using the unicellular freshwater alga to *Raphidocelis subcaptitata* was performed according to DIN 38412-59.(*86*) In this standardized assay, algal growth rate was monitored over 72 h where toxic compounds may inhibit growth. DCP was used as a positive control and assay medium as a negative control (indicating 0 % inhibition).

DFX was dissolved into assay medium and tested at four concentrations and no algal growth rate inhibition was observed at the tested concentrations (fig. 6C). The test was valid according to the following criteria: specific 3 day growth rate (μ) of controls > $1.2.d^{-1}$ (assay value was $1.67.d^{-1}$); CV of 3 day μ of controls < 7 % (assay value was 1.3 %); average CV of control replicate growth rate (μ) on each of three days < 35% (assay value was 25 %); inhibition by the positive control between 20 and 80 % (assay value was 30 %).

Estrogenic effects using the Lyticase-Yeast Estrogen Screen (L-YES). L-YES was performed on 96-well plates based on ISO 19040-1 (2018).(*81,82*) A dilution series of E2 served as a reference compound (positive control), in triplicate. Compounds were either dissolved in water (DFX) or DMSO (all other compounds) and tested, in duplicate, in two-fold dilution over eight dilution steps. Cytotoxicity to yeast cells, indicated by reduced optical density of the yeast in test wells, occurred at higher concentrations of H ester and G ester (figs. S26 and S32, tables S10 and S11).

Supplementary Figures

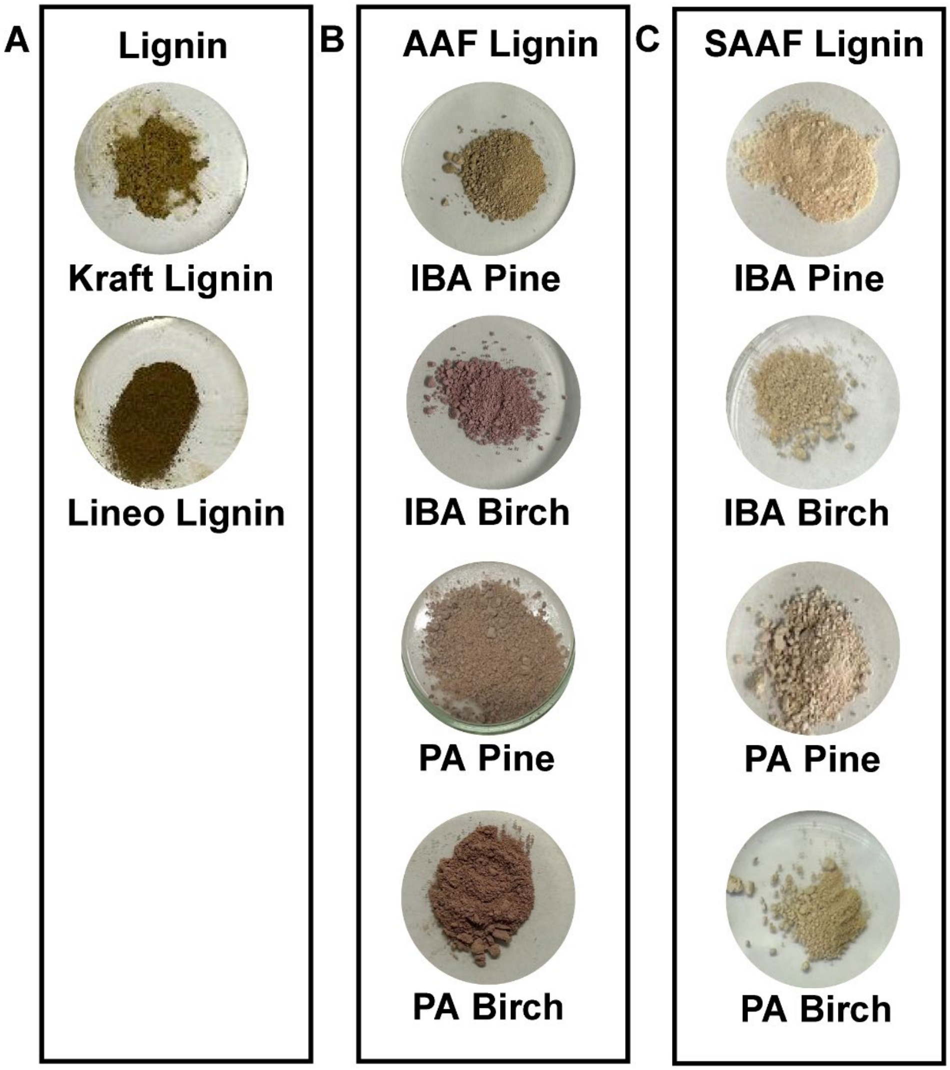

Fig. S1. Physical appearance of lignins. Photographs of (**A**) commercial lignins (Kraft, Lineo); (**B**) AAF lignins (isobutyraldehyde (IBA) Pine, IBA Birch, propionaldehyde (PA) Pine, PA Birch); (**C**) SAAF lignins (IBA Pine, IBA Birch, PA Pine, PA Birch).

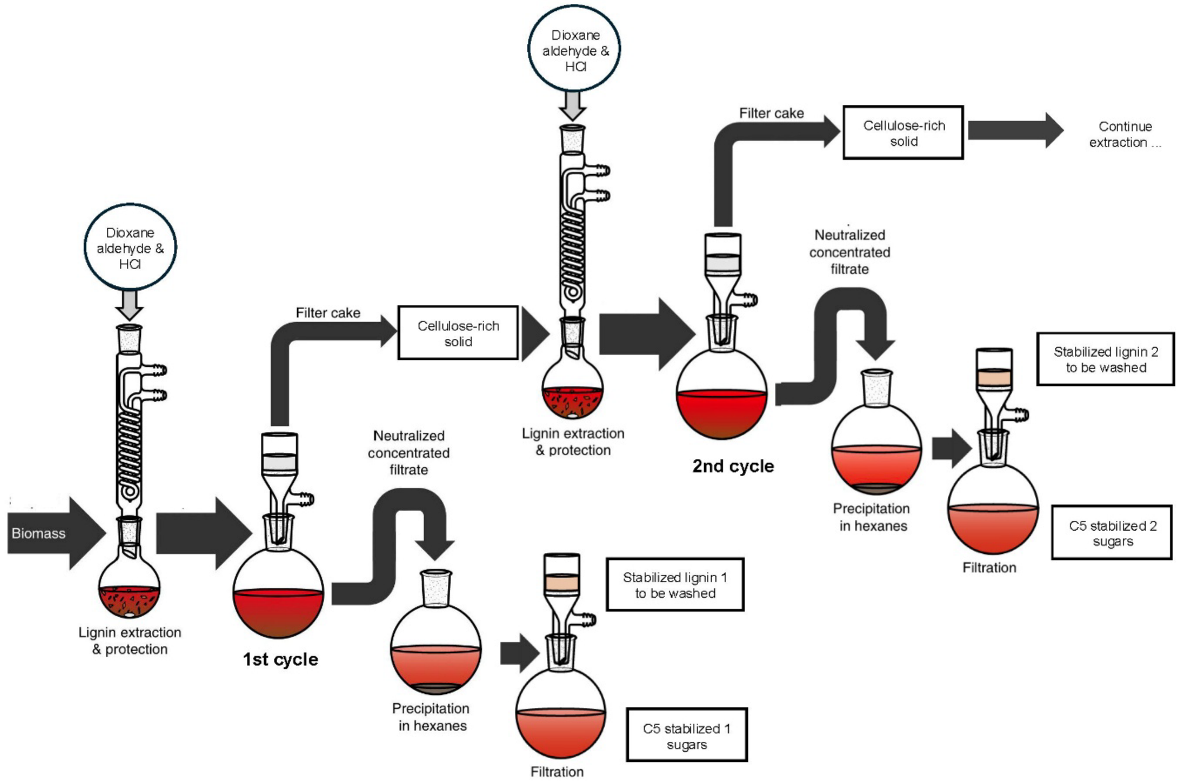

Fig. S2. SAAF procedure. An overview of the sequential extraction procedure, which yields several batches of lignin following increasing cycles of AAF extraction.

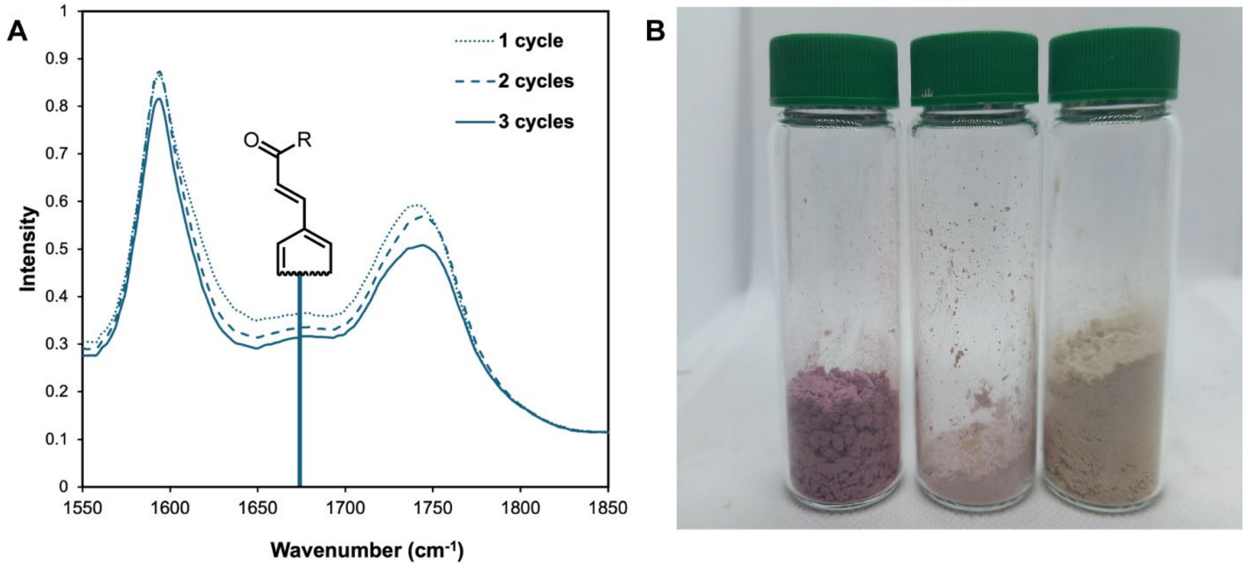

Fig. S3. SAAF Lignins. (**A**) Fourier transform infrared (FT-IR) spectra graph of SAAF lignins extracted using three cycles of 1, 2 and 3 h respectively. (**B**) Evolution of extracted lignin (Birch wood, PA aldehyde) color during the SAAF process. From left to right: 30 min, 1 h, 2 h of total extraction time from the cellulose-rich solids.

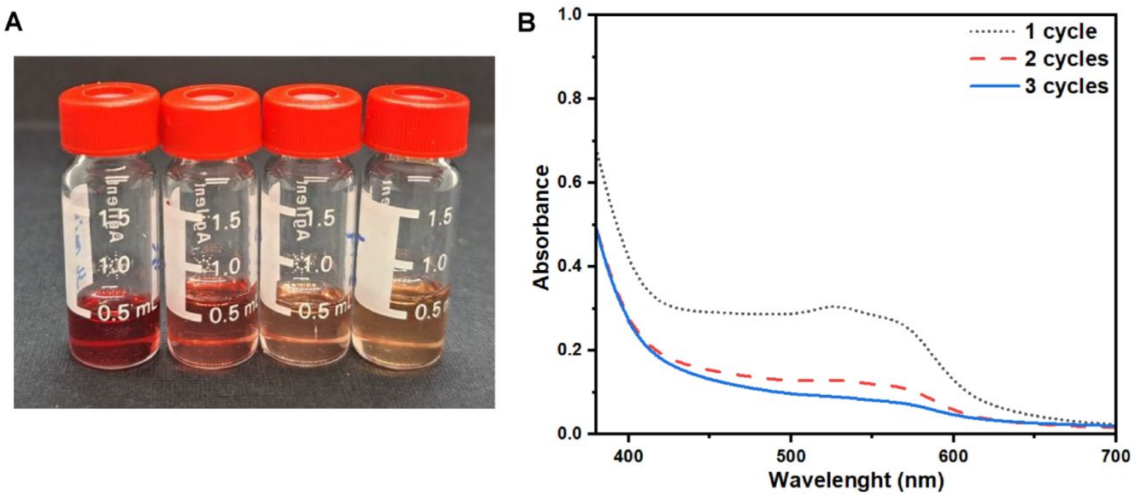

Fig. S4. Color evolution of SAAF lignins. (**A**) Evolution of extracted lignin color during the SAAF process after dilution in dioxane (10 mg/mL). From left to right: 1 cycle (30 min), 2 cycles (2 x 30 min), 3 cycles (3 x 30 min), 4 cycles (4 x 30 min) of total extraction time from the cellulose-rich solids; (**B**) UV-Vis spectra of SAAF lignin solution extracted 1 cycle (30 min), 2 cycles (2 x 30 min), 3 cycles (3 x 30 min) hours. The solutions were prepared with equal mass concentrations in dioxane as the solvent. The data are corrected for the dioxane background.

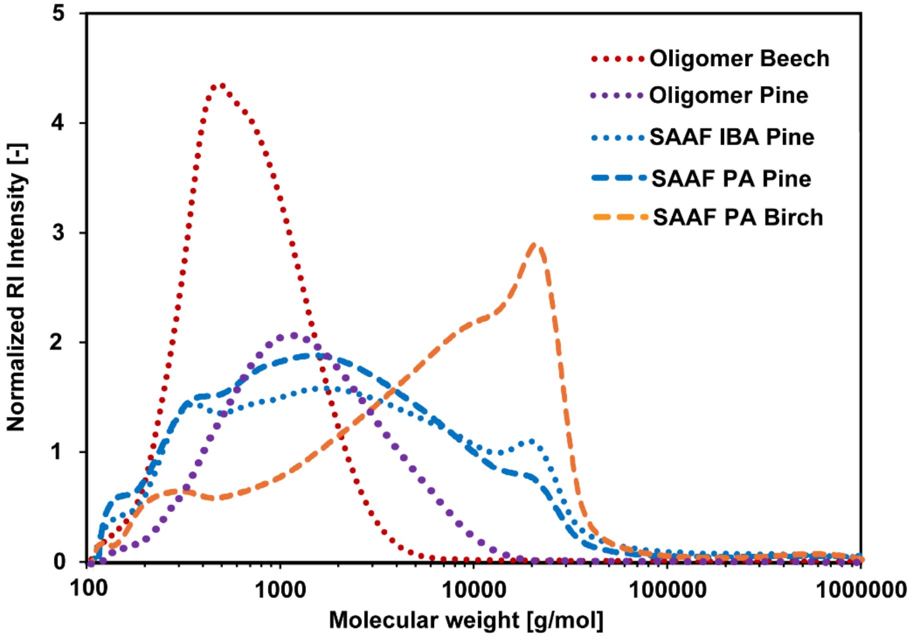

Fig. S5. Size Exclusion Chromatography (SEC). SEC traces of SAAF Lignin IBA Pine, SAAF Lignin PA Birch, SAAF Lignin PA Pine, Oligomer Birch and Oligomer Pine. The RI signal was normalized to peak area.

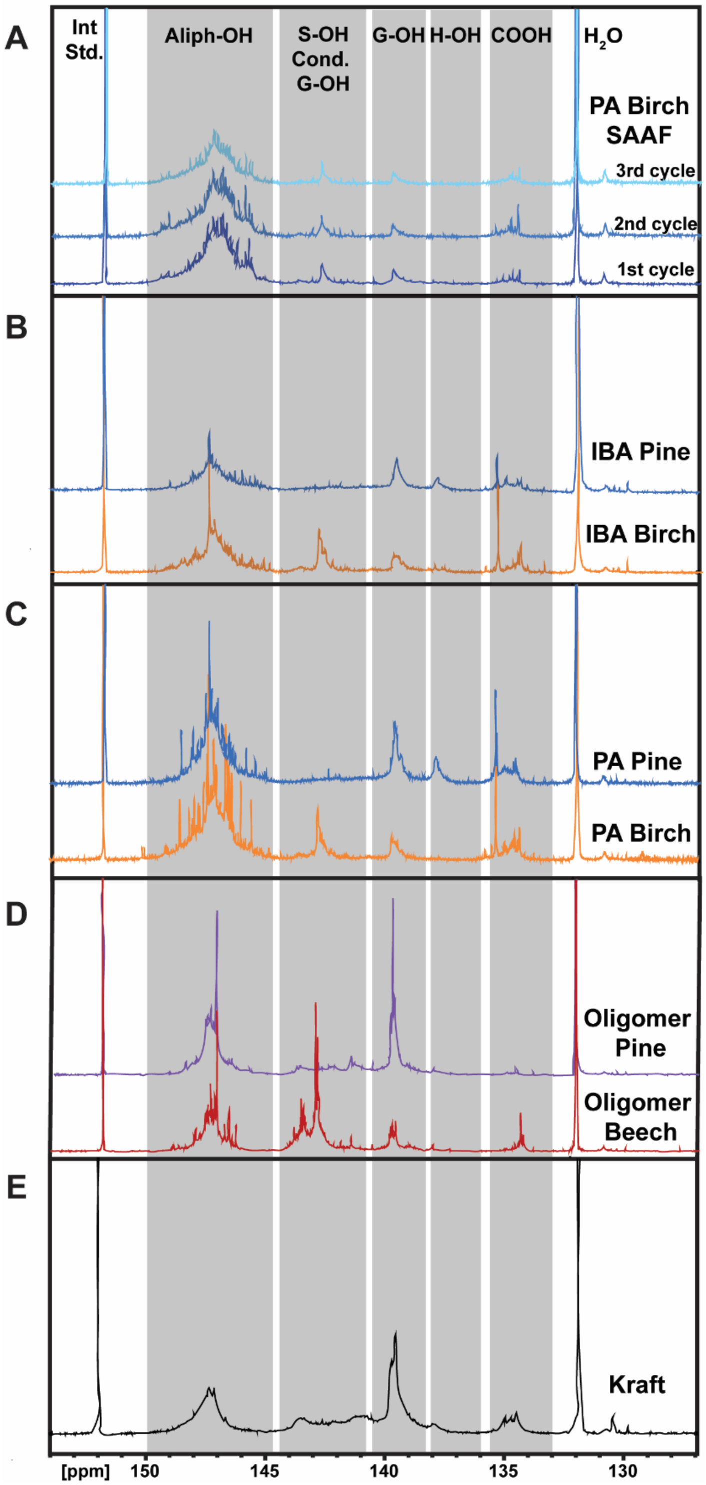

Fig. S6. Quantitative ^{31}P NMR spectra of lignins. Derivatized with TMDP using NHND as the internal standard. Conditions: $CDCl_3$, 600 MHz.(**A**) SAAF lignin after 1, 2 or 3 extraction cycles (**B**) selected IBA protected lignins, from Birch (orange) and Pine (blue) (**C**) selected PA protected lignins, from Birch (orange) and Pine (blue) (**D**) lignin oligomers from Beech (red) and Pine (purple) and (**E**) Kraft lignin from Pine.

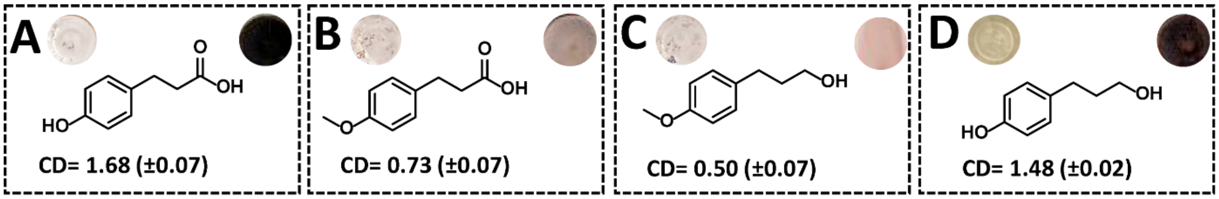

Fig. S7. Initial color activity test model compounds. (**A-D**) Photographs of color activity test before and after color development, molecular compound, and color density (C.D.) after heating, in the octadecanol matrix, while maintaining equimolar ratio between dye and model compound developer.

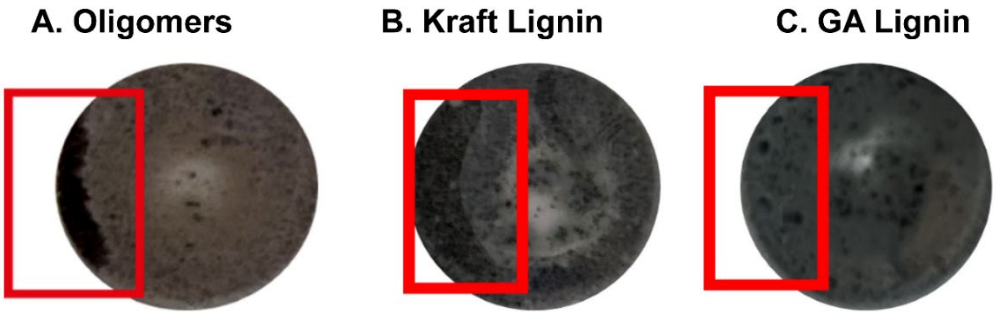

Fig. S8. Heterogeneity issues. Color activity test. Zoomed-in photograph of developer mixed with octadecanol (100 mg) after heating for 30 s at 100 °C and cooling down to room temperature. The formulation was selected as followed: OBD-2 : developer = 1 : 2 mass ratio constant and show phase separation domains of (**A**) Oligomers, (**B**) Kraft Lignin, (**C**) GA AAF lignin in the polar octadecanol matrix.

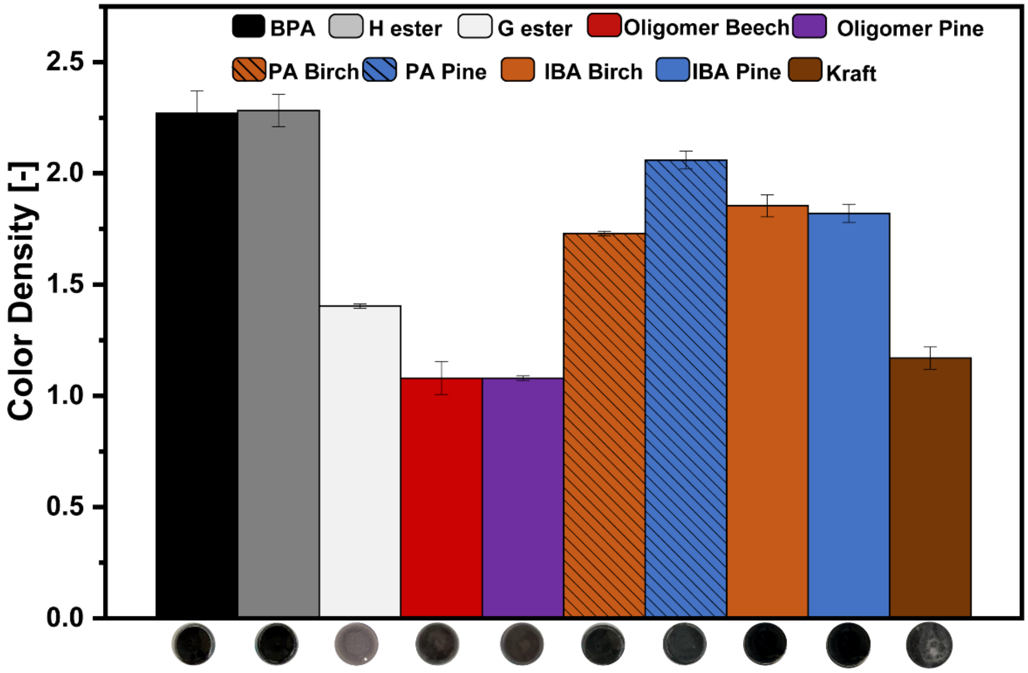

Fig. S9. Initial color activity test. C.D. evaluation, using CIE *Yxy* values, after heating a mixture of octadecanol (100 mg) and developer : OBD-2 (20 mg : 10 mg, 2 : 1 mass ratio) for 30 s at 100 °C and cooling it to room temperature.

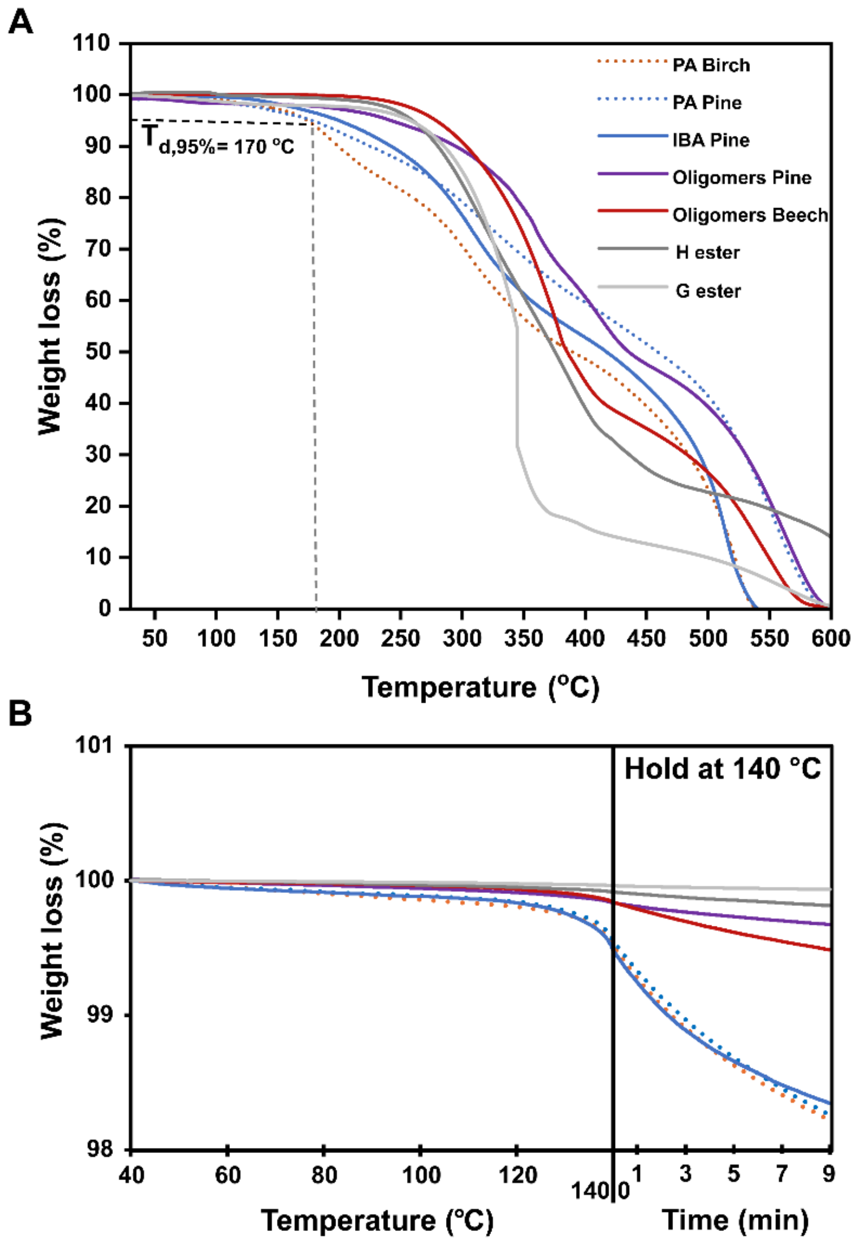

Fig. S10. Thermogravimetric analysis (TGA). (**A**) TGA performed under air to mimic printing conditions. (**B**) TGA with temperature hold at 140 °C .

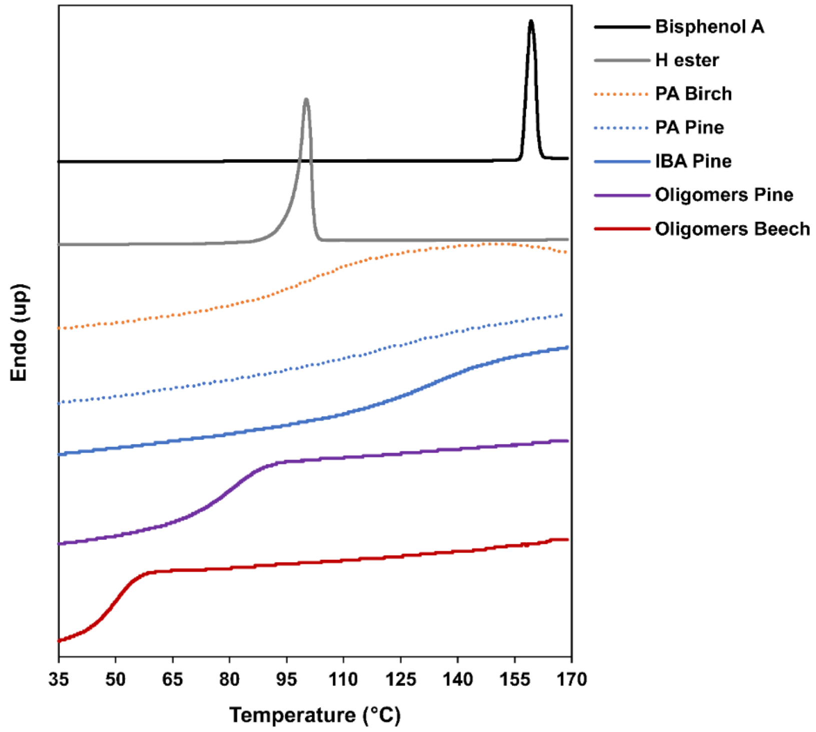

Fig. S11. DSC Analysis of color developers. DSC measurements for BPA and all solid lignin-based color developers. Apart from BPA and the H ester, no melting peaks were observed between 30 °C and 170 °C.

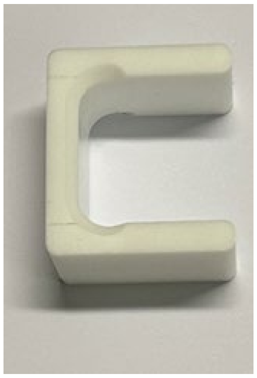

Fig. S12. Coater. Manual U-coater utilized for thermal paper coating.

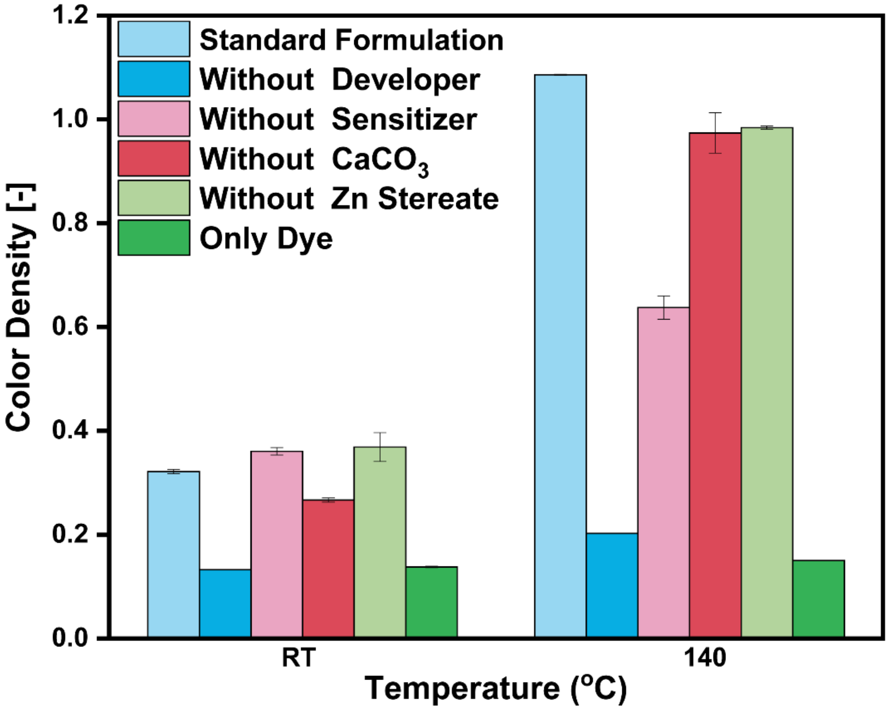

Fig. S13. Impact of individual ingredients on C.D. Impact of additives in C.D. reached at room temperature and 140 °C. No color development is measured without the dye or the developer. This confirms the role of lignin as a proton transfer agent and the absence of degradation reaction at 140 °C that could have led to color density variation. Reduced performance was observed without the sensitizer, indicating limited proton transfer between the developer and the dye. Calcium carbonate and zinc stearate do not impact the dye development, only the processability.

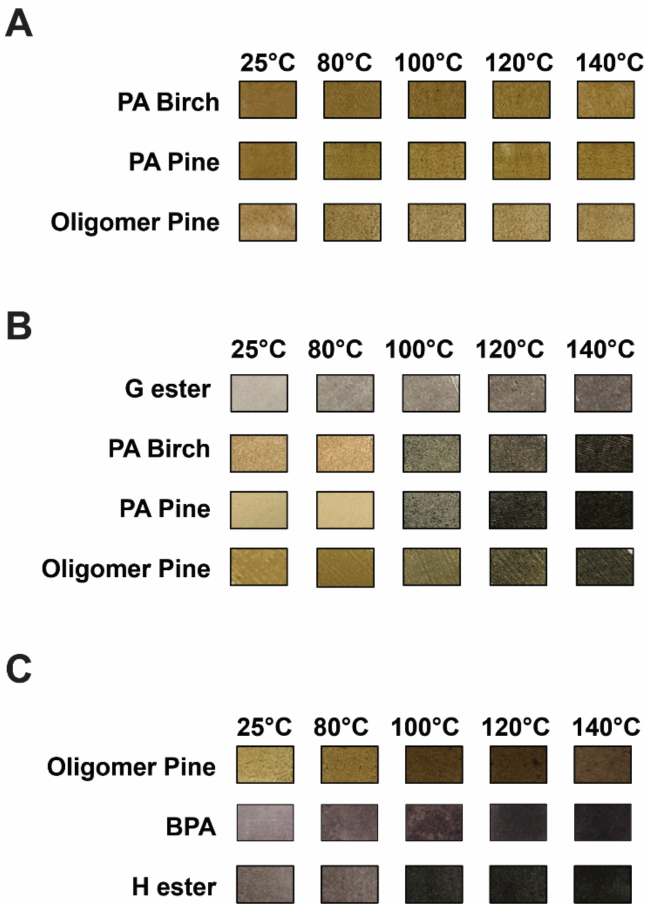

Fig. S14. Complementary static sensitivity pictures of thermal paper formulations. (**A**) without sensitizer, (**B**) with benzalphtalide as sensitizer, (**C**) with DFX as sensitizer, with various color developers at increasing heating temperatures (corresponding static sensitivity curves are shown in fig. 3).

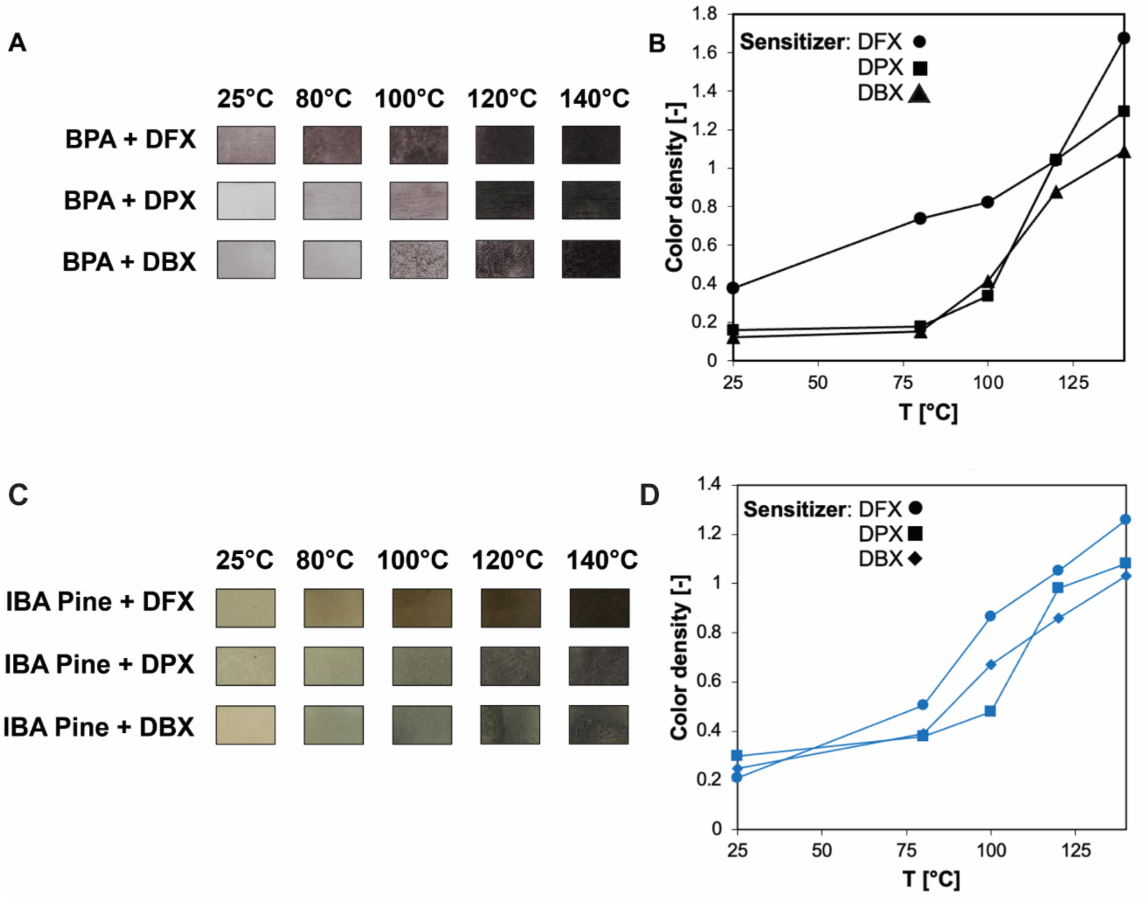

Fig. S15. Thermal paper formulation with varying sugar sensitizers. Thermal paper formulation with BPA (A,B) and IBA Pine (C,D) used as the developer and with different sugar-based sensitizers at increasing heating temperatures. (A,C) Static sensitivity pictures, (B,D) Static sensitivity curves of thermal paper formulations. DFX: Diformylxylose, DBX: Dibutylxylose; DPX: Dipropylxylose. The preparation and characterization data for DBX and DPX was previously reported by Komarova *et al*.(*94*).

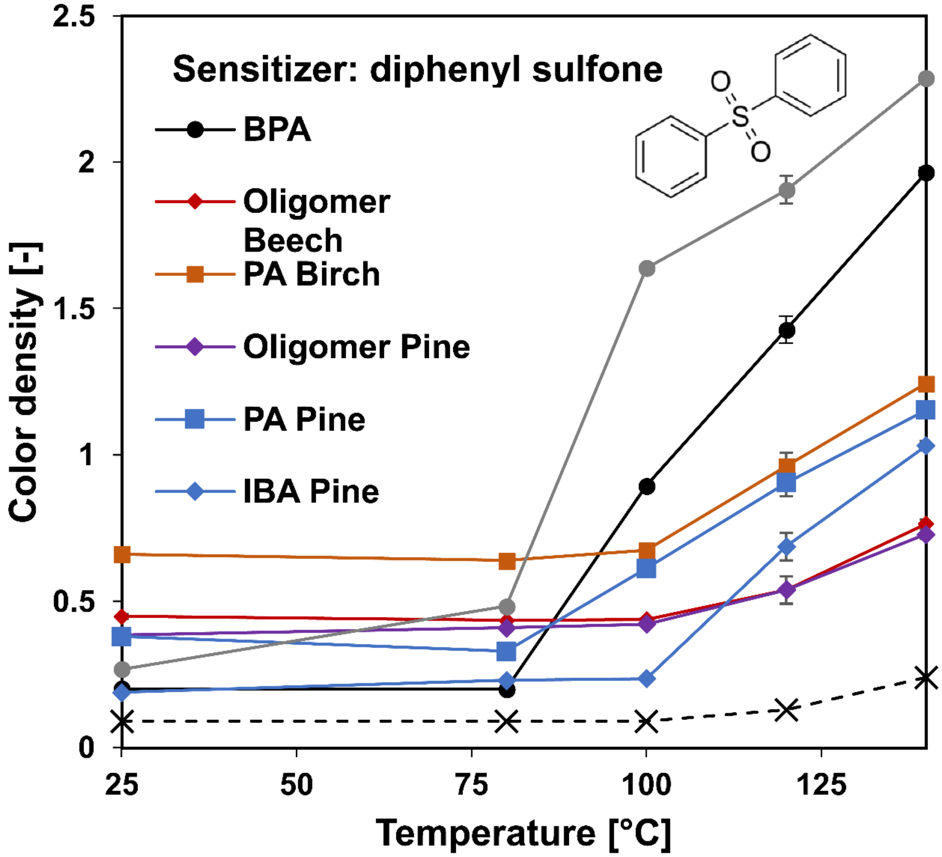

Fig. S16. Diphenylsulfone sensitizer. Static sensitivity curves of thermal papers coated with diphenylsulfone as sensitizer at various temperature (25 °C to 140 °C). Color density was evaluated using CIE *Yxy* values. Dashed lines represent control experiment with identical formulations but without any developer.

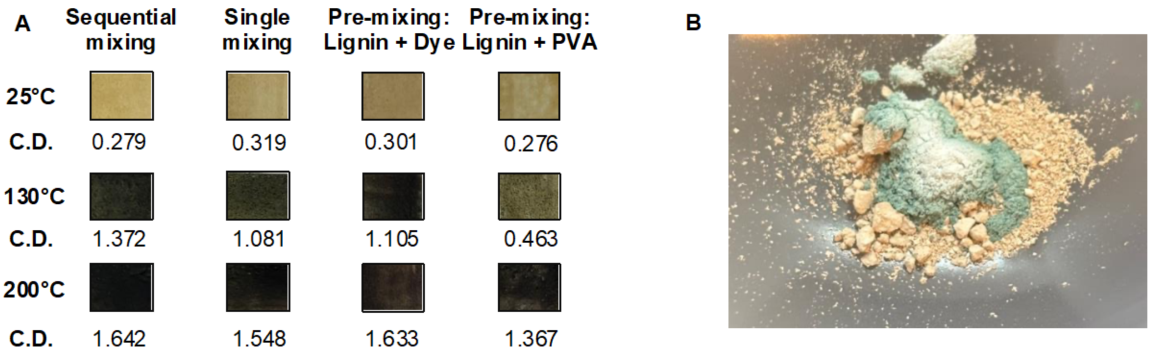

Fig. S17. Sequential mixing. (**A**) Evolution of coating static sensitivity depending on the mixing sequence. (**B**) Light color development is already visible at room temperature when readily putting in contact the lignin and the dye.

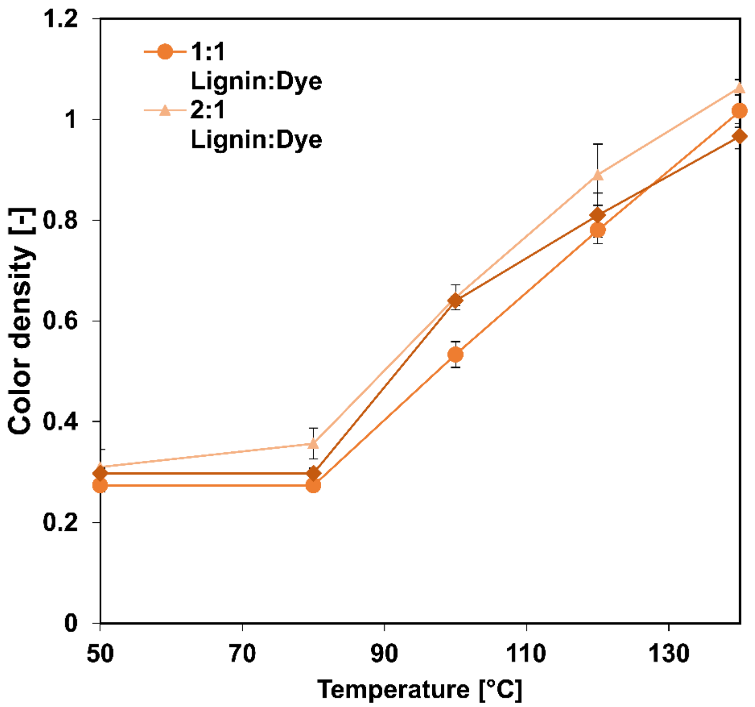

Fig. S18. Different developer : dye ratios for thermal coatings. Effect of temperature on color density for formulation with different lignin:dye molar ratios (1:1, 2:1, and 1:2). Despite differences in stoichiometry, all formulations exhibited similar color development profiles across the tested temperature range, suggesting that compatibility and diffusion within the matrix were the dominant factors influencing reactivity.

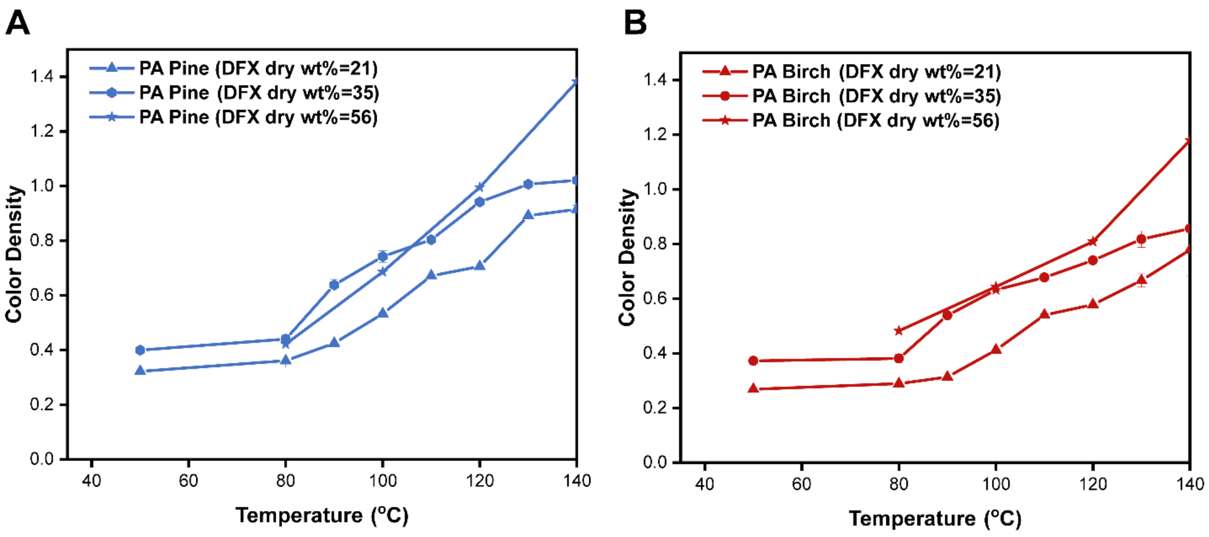

Fig. S19. DFX sensitizer content optimization. Static sensitivity curves of thermal papers coated with DFX (9, 15 and 30 wet wt.%, 21, 35 and 56 dry wt.%) as sensitizer for (**A**) SAAF PA Pine and (**B**) SAAF PA Birch wood lignin as developer.

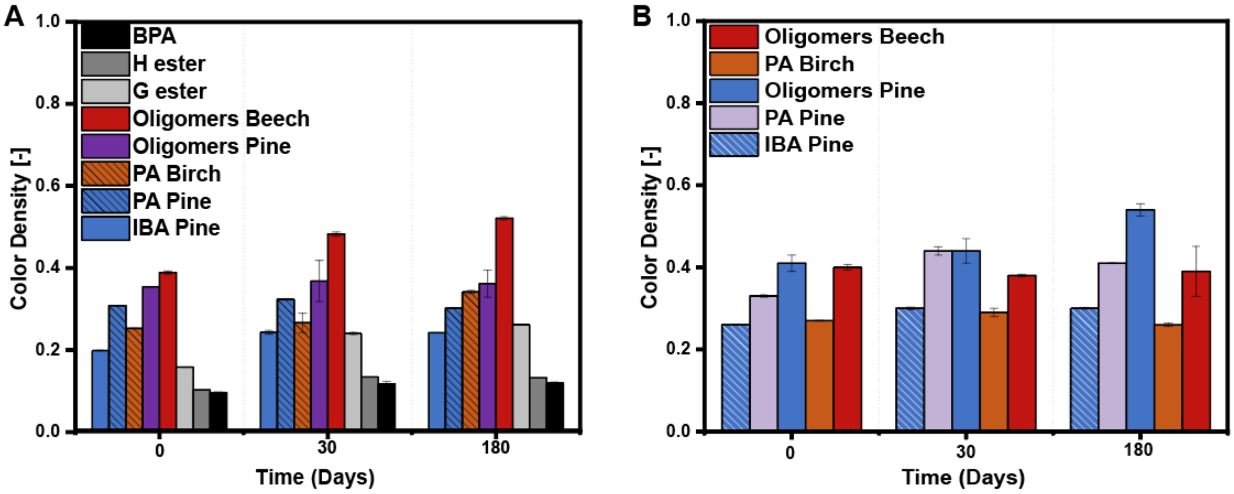

Fig. S20. Coating Stability. Color densities after different storage times by the window before heating (i.e. at room temperature) for coatings with the following sensitizers: **(A)** benzalphtalide and **(B)** DFX. Thermal paper samples were left on a bench next to a window to evaluate coating stability over time under ambient condition.

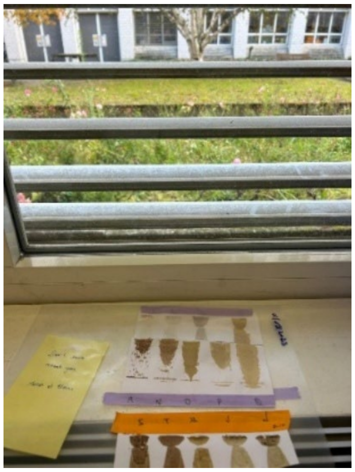

Fig. S21. **Thermal coating stability test.** Photography of the coating light exposure experiment, illustrating the setup used to assess the coating stability under ambient conditions.

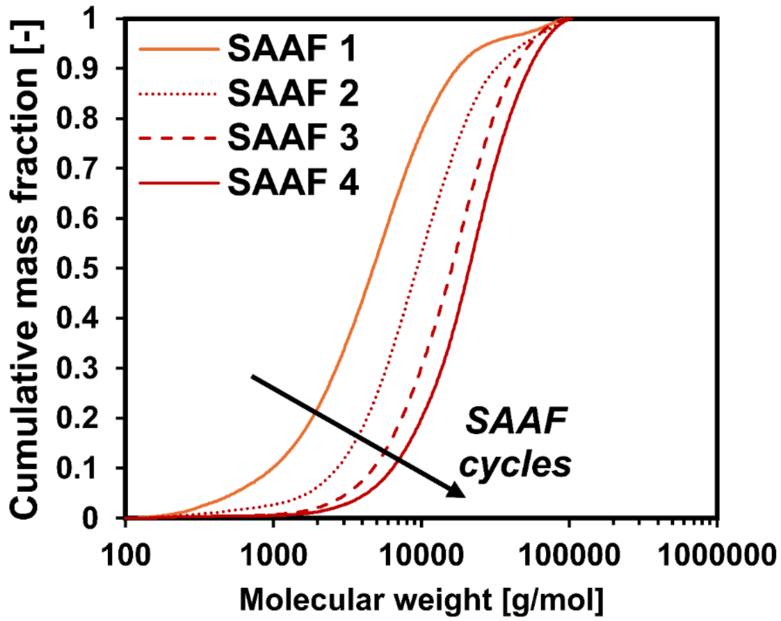

Fig. S22. SAAF cycles cumulative mass fractions. Comparison of cumulative mass fraction as a function of molecular weight (g/mol) for PA Birch during SAAF extraction depending on the number of extraction cycles performed.

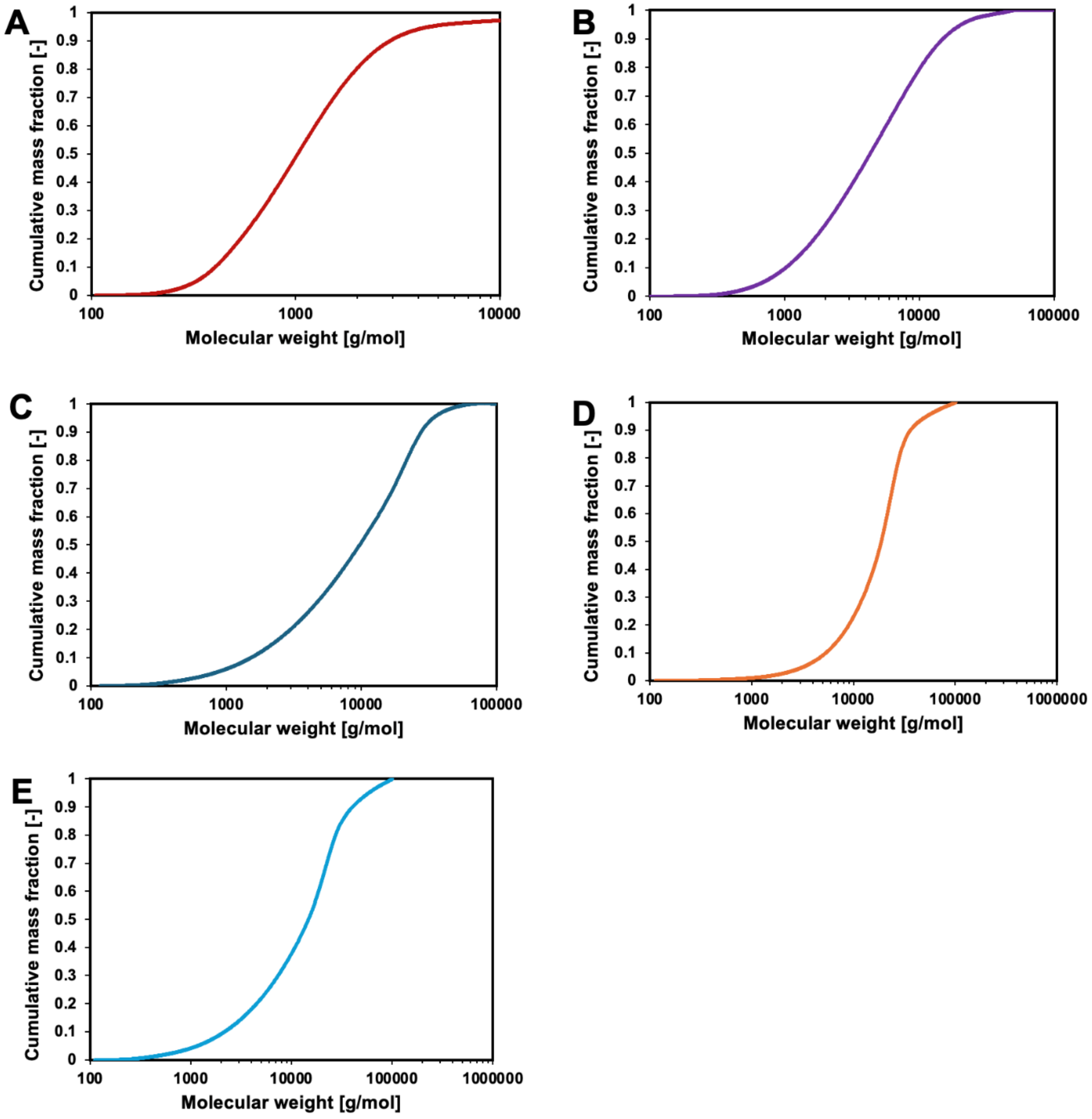

Fig. S23. Lignins cumulative mass fractions. Comparison of the cumulative mass fraction as a function of molecular weight (g/mol) for (**A**) Oligomer Beech (**B**) Oligomer Pine (**C**) PA Pine (**D**) PA Birch (**E**) IBA Pine.

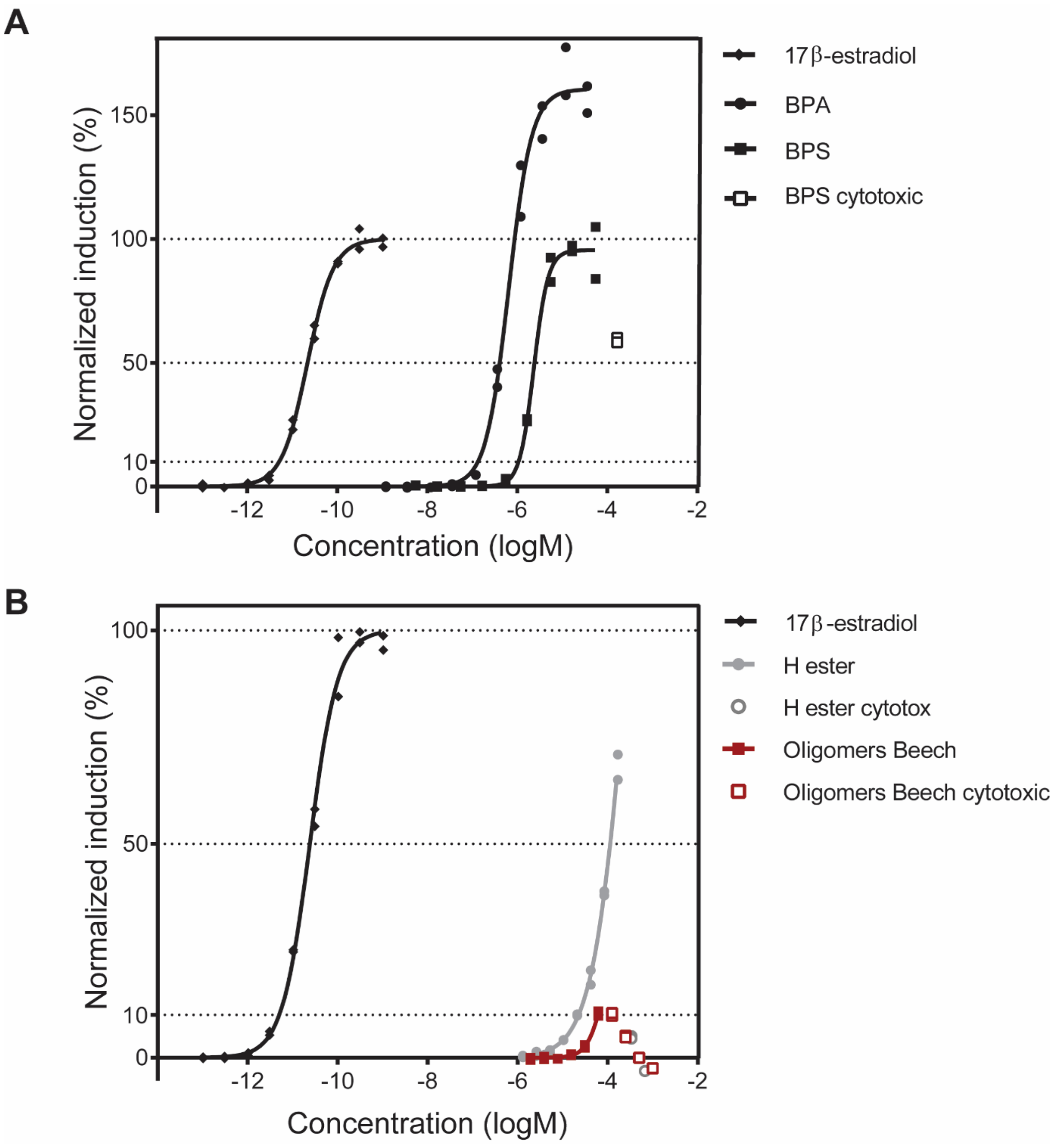

Fig. S24. ERα-CALUX: plots for agonistic estrogenic effects of lignin developers. Normalized agonistic ERα-CALUX estrogenic activity data of 17β-estradiol (reference) and (**A**) BPA and BPS; (**B**) H ester and Oligomers Beech. Concentrations with cytotoxic effects are indicated by open symbols**.** Crossing the x-axis below 0% induction is possible if cells are so damaged that their background signal reduces to below that of healthy unexposed control cells.

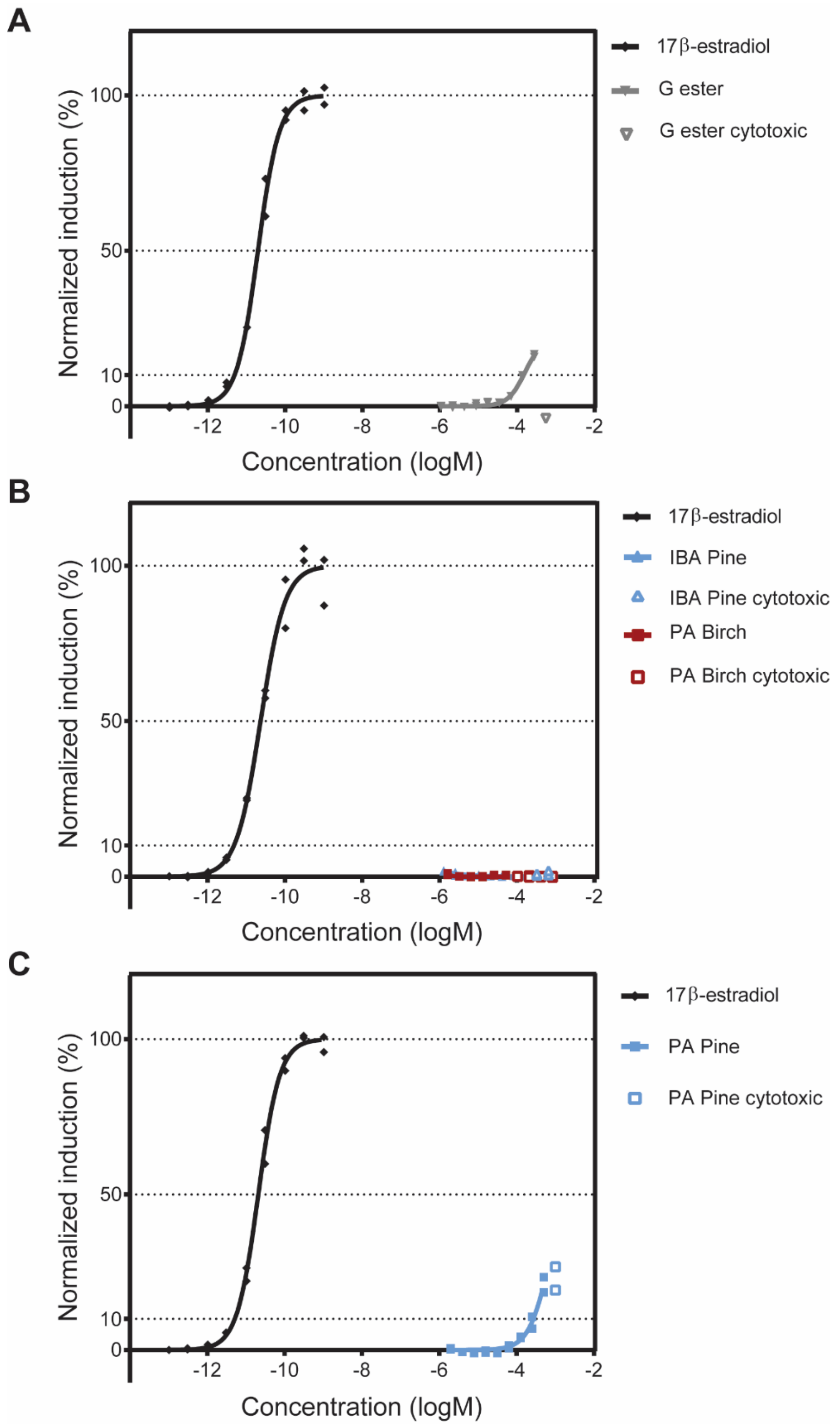

Fig. S25. ERα-CALUX: plots for agonistic estrogenic effects of lignin developers. Normalized agonistic ERα-CALUX estrogenic activity data of 17β-estradiol (reference) and (**A**) G ester (**B**) IBA Pine and PA Birch, (**C**) PA Pine. Concentrations with cytotoxic effects are indicated by open symbols Crossing the x-axis below 0% induction is possible if cells are so damaged that their background signal reduces to below that of healthy unexposed control cells.

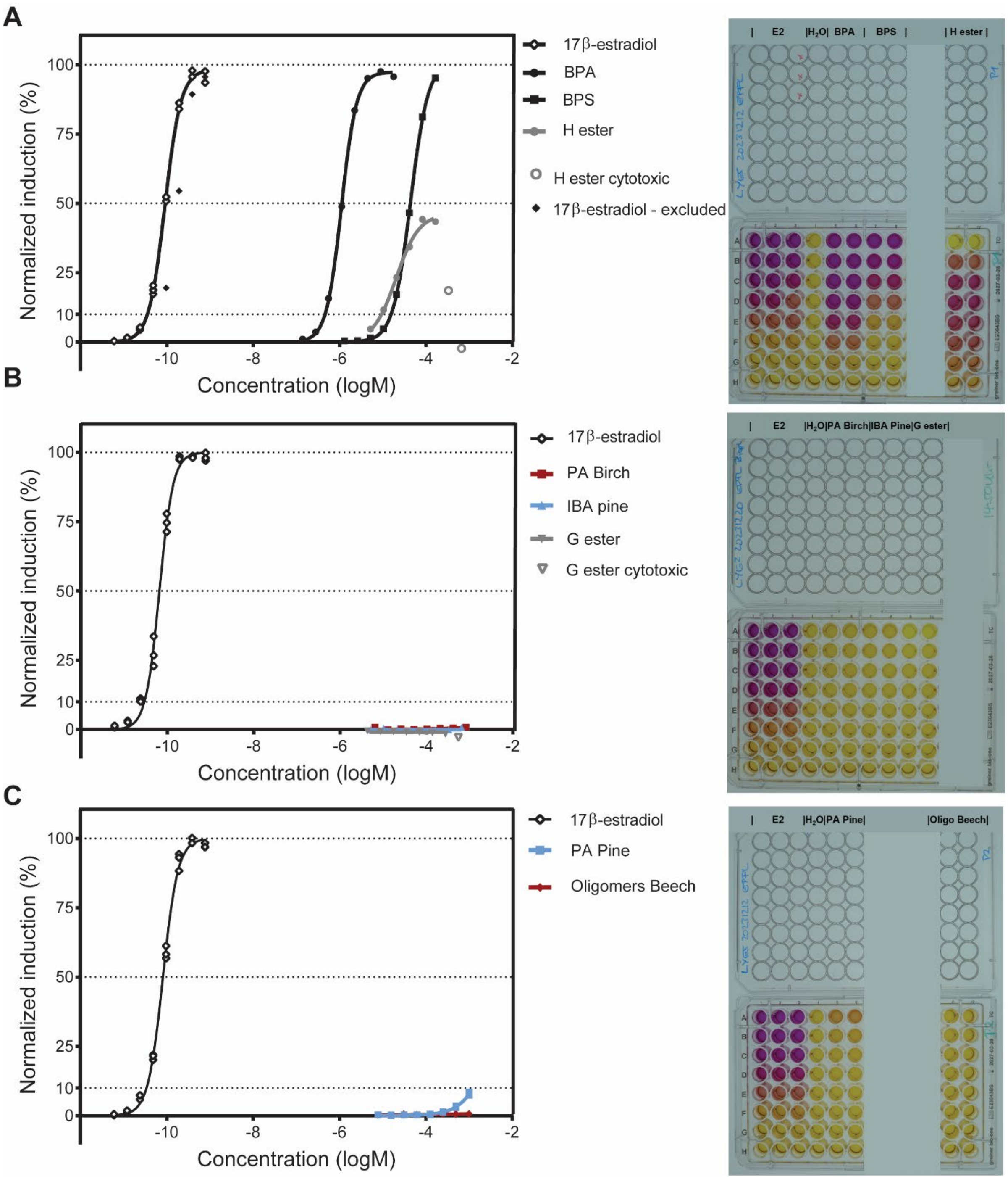

Fig. S26. LYES: plots for agonistic estrogenic effects of lignin developers. Normalized agonistic L-YES estrogenic activity data, as well as pictures from L-YES plates of (**A**) BPA, BPS, H ester; (**B**) PA Birch, IBA Pine, G ester (**C**) PA Pine, Oligomers Beech. Each assay plate has a 17β-estradiol (reference) curve. Concentrations with cytotoxic effects are indicated by open symbols. Parts of the 96-well plates are not shown, this concerns wells containing compounds not described in this study.

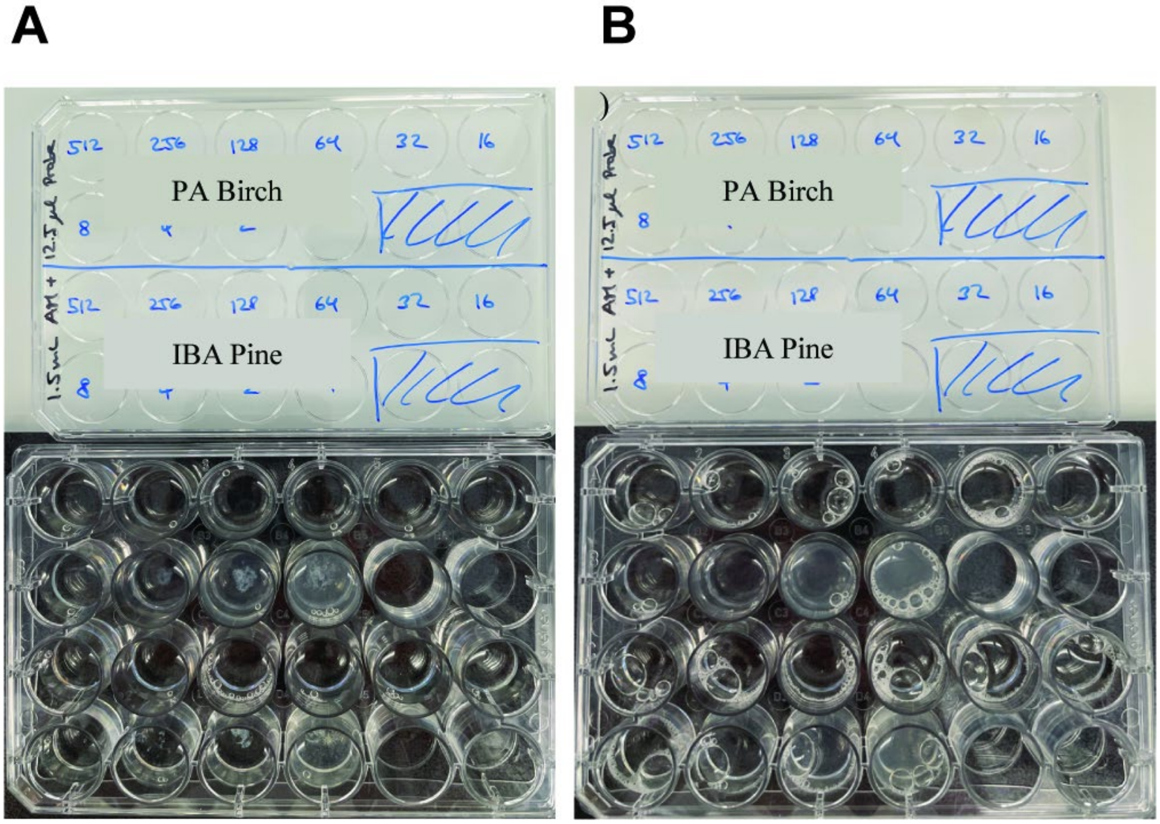

Fig. S27. (A) Concentration series of PA Birch and IBA Pine in ERα-CALUX assay medium on 24-well plates, precipitation is visible in the highest concentrations labelled 1-8 (**B**) Following mixing of the wells, suspensions were obtained.

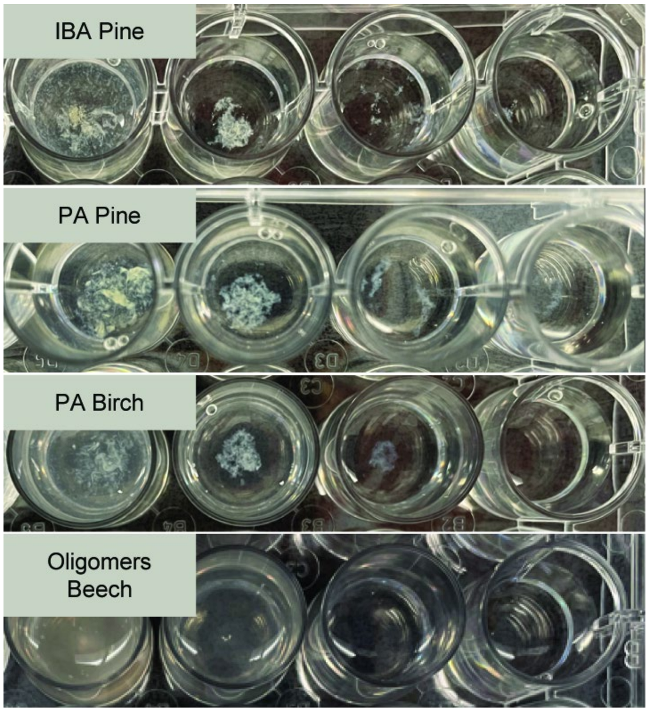

Fig. S28. Images of wells with ERα-CALUX assay medium containing the four highest tested concentrations of four tested compounds before mixing of the 24-well plates.

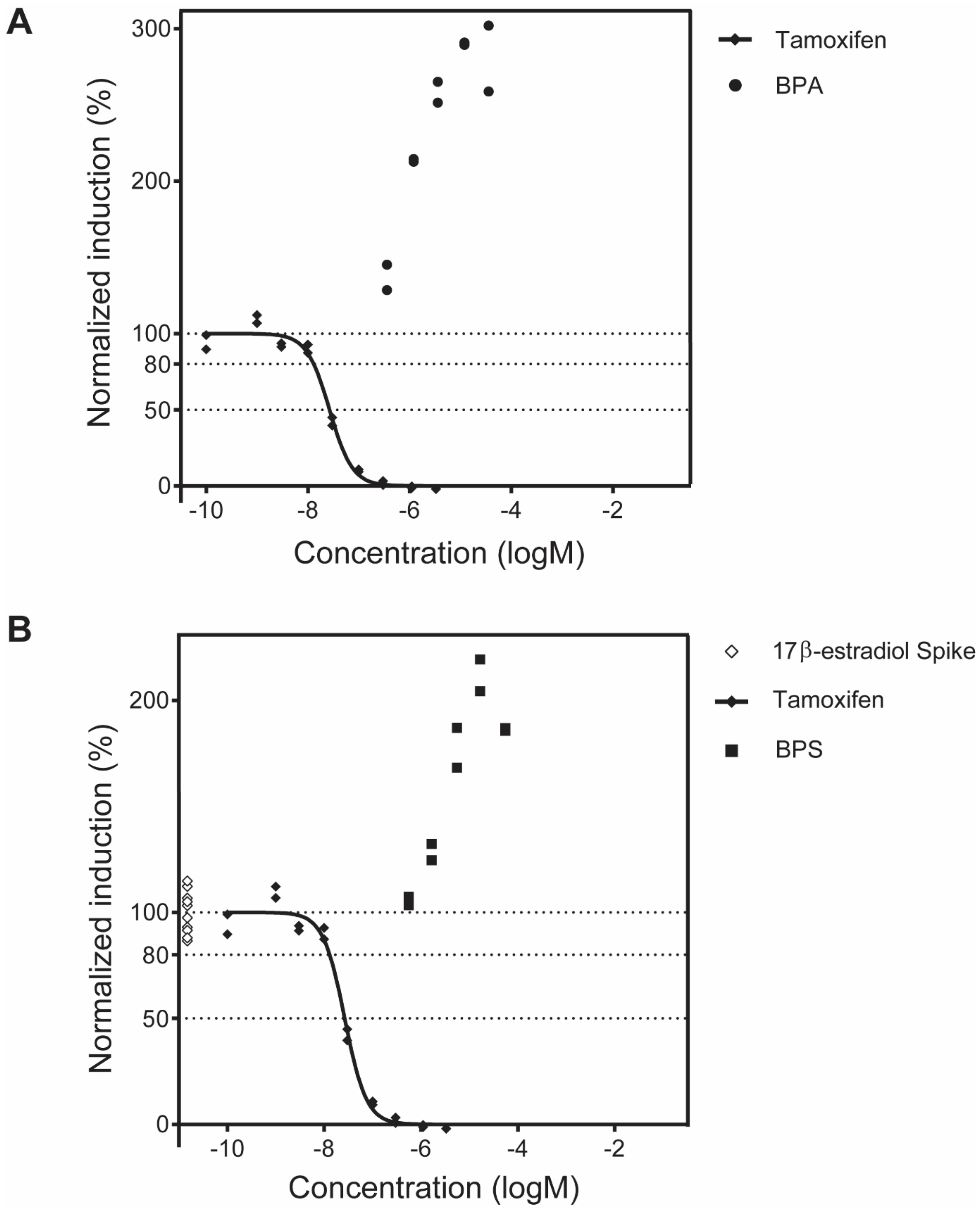

Fig. S29. ERα-CALUX: plots for antagonistic estrogenic effects of lignin developers. Normalized antagonistic estrogenic activity data of tamoxifen (reference) and: (**A**) BPA, only agonistic effects are observed; (**B**) BPS, only agonistic effects are observed (although induction at the highest BPS concentration of 5.5 10^{-5} M drops a bit, no cytotoxicity was observed)

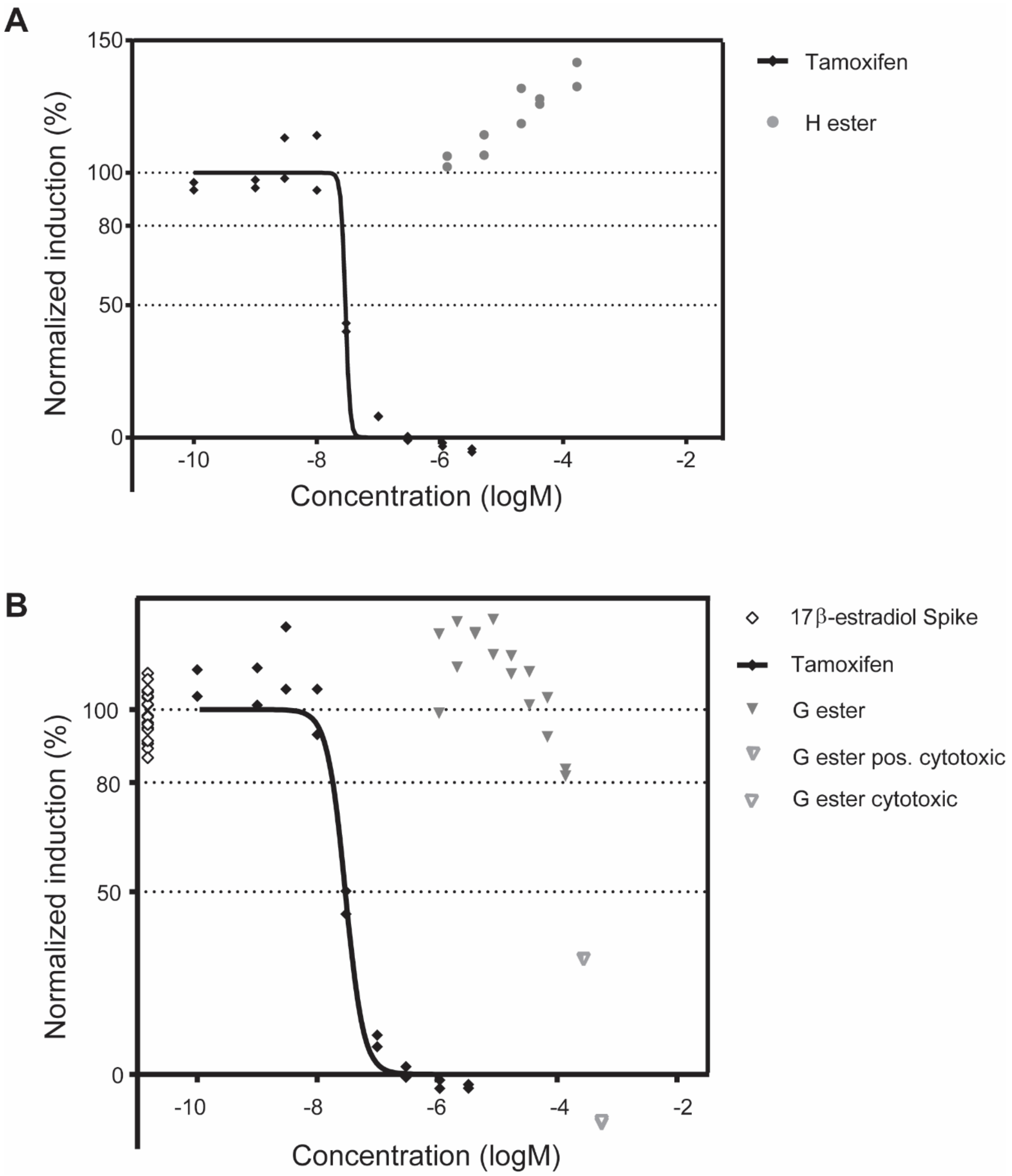

Fig. S30. ERα-CALUX: plots for antagonistic estrogenic effects of lignin developers. Normalized antagonistic estrogenic activity data of tamoxifen (ref.) and: (**A**) H ester; (**B**) G ester, the highest concentration was clearly cytotoxic, the second highest concentration (i.e. $2.8\ 10^{-4}$ M) was possibly cytotoxic. Twenty additional wells served as 17β-estradiol spike control.

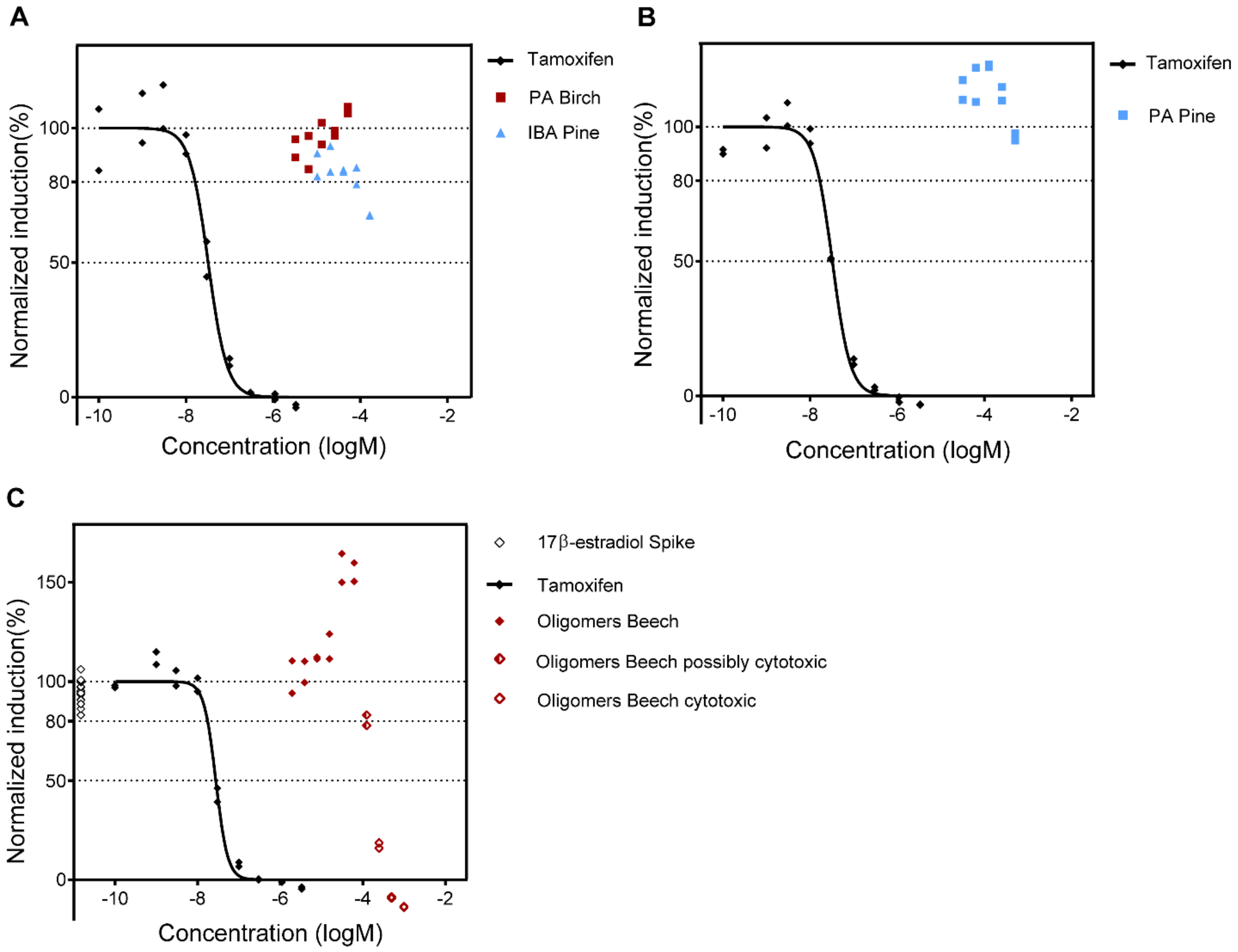

Fig. S31. ERα-CALUX: plots for antagonistic estrogenic effects of lignin developers. Normalized antagonistic estrogenic activity data of tamoxifen (reference) and: (**A**) IBA Pine and PA Birch, cytotoxicity for the two highest IBA Pine concentrations (2 and 1 10^{-5} M) could not be determined conclusively due to the presence of particles. It is thus unclear if IBA Pine shows slight antagonistic effects or if the induction below 80% is caused by cytotoxicity; (**B**) PA Pine; (**C**) Oligomers Beech: three of the highest concentrations were clearly cytotoxic, the fourth highest concentration (i.e. 1.2 10^{-4} M) was possibly cytotoxic. Eighteen additional wells served as 17β-estradiol spike control.

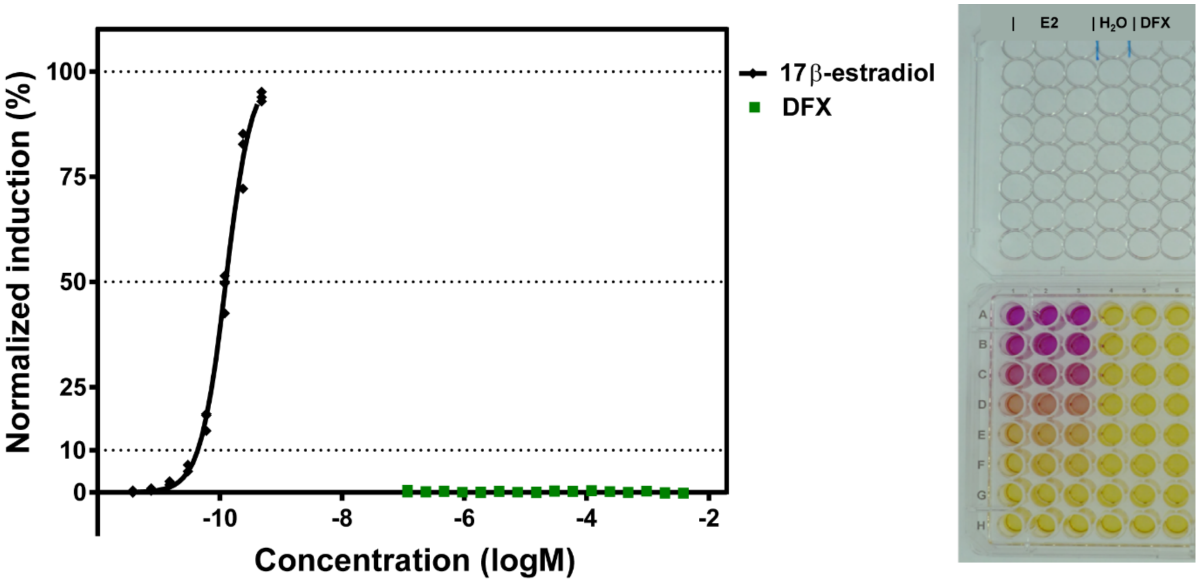

Fig. S32. LYES: agonistic estrogenic effects of DFX. Normalized agonistic L-YES estrogenic activity data of 17β-estradiol (reference) and DFX, as well as picture from L-YES plate.

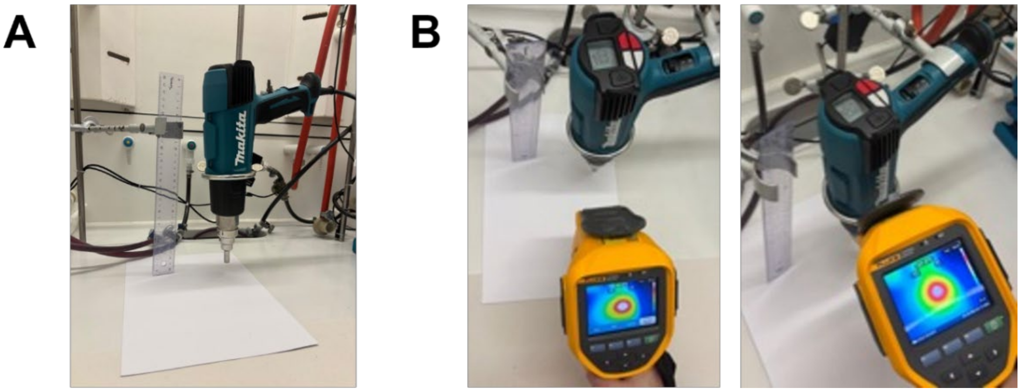

Fig. S33. Heat gun set-up. (**A**) Experimental setup used to study the static sensitivity of thermal paper formulations. A Makita heat gun (HG6531CK) is employed to apply heat in the range of 50 to 140 °C. The nozzle of the heat gun is positioned 1.5 cm above the surface of the paper during exposure. (**B**) Surface temperature measurement of thermal paper using a Fluke Ti480 thermal imager. The paper was exposed to heat from a Makita HG6531CK heat gun, and temperature readings were taken to assess thermal response.

Supplementary Tables

Table S1. Use of color developers in thermal papers. Evolution in time and all over the world. Sample size is the amount of collected thermal paper receipts. The most prominent non-bisphenol alternative is "Pergafast 201". Other examples are "Appvion Alpha Free" (ascorbic acid), "BPSIP" and "NKK-1304.(*22*)

			Fraction of samples containing developer (%)				
Location	Year	Number of samples	BPA	BPS	Pergafast	Others	ref
USA	2017	167	18.0	75.4	1.2	4.8	(*22*)
	2022	571	1.2	84.6	12.3	2.0	
Switzerland	2014	129	82.2	3.1	7.8	7.0	(*25*)
	2019	185	48.8	14.6	30.8	6.0	
	2021	194	10.8	19.1	60.3	9.8	
Europe	2019	238	67.6	15.1	12.6	4.6	(*24*)
Asia	2019	26	50.0	23.1	26.9	/	
Oceania	2019	19	84.2	5.3	/	10.5	
North America	2019	25	16.0	84.0	/	/	
Germany	2015	114	48.2	11.4	34.2	6.1	(*23*)
	2016	98	46.9	9.2	33.7	7.1	
	2017	99	52.5	6.1	40.4	1.0	
Bangladesh	2021	67	67.5	25.0	/	/	(*26*)

Table S2. ***CIE L*a*b** values of lignin powders.*** (**A**) commercial lignins (Kraft, Lineo); (**B**) AAF lignins (isobutyraldehyde (IBA) Pine wood lignin, IBA Birch, propionaldehyde (PA) Pine, PA Birch); (**C**) SAAF lignins (IBA Pine, IBA Birch, PA Pine, PA Birch).

	*L**	*a**	*b**	*Y*	*x*	*y*
White Calibrant (Control)	91.27	-13	01.03	79.08	0.3155	0.3331
Black Calibrant (Control)	6.28	-3.58	06.07	0.71	0.3402	0.4022
BPA	79.80	-4.76	6.76	56.34	0.3199	0.3494
Kraft Lignin	49.27	7.41	26.79	17.82	0.4101	0.4001
Lineo Lignin	43.60	7.09	20.31	13.57	0.3983	0.3871
SAAF Lignin IBA Pine	73.04	3.20	19.34	45.26	0.3616	0.3720
SAAF Lignin IBA Birch	69.54	0.48	16.04	40.10	0.3511	0.3688
SAAF Lignin PA Pine	68.16	0.79	19.84	38.19	0.3621	0.3774
SAAF Lignin PA Birch	70.37	0.37	15.69	41.29	0.3497	0.3677
AAF Lignin IBA Pine	62.99	3.13	18.98	31.58	0.3666	0.3762
AAF Lignin IBA Birch	58.89	14.36	5.89	26.91	0.3586	0.3319
AAF Lignin PA Pine	60.97	0.64	17.97	29.21	0.3559	0.3759
AAF Lignin PA Birch	59.60	14.49	06.01	27.69	0.3587	0.3320

Table S3. Characteristics of three Birch lignin SAAF batches. Extracted after 1 to 4 AAF cycles from the cellulose-rich solids respectively (with PA aldehyde): size-exclusion chromatography (SEC), FTIR, ^{31}P NMR. The alpha-beta-unsaturated ketone peak monitored by FTIR (1685-1666 cm^{-1}) can be assigned lignin chromophores.(*56-58*)

**Cycles – (extraction time)**	**1 - (30min)**	**2 - (1 h)**	**3 - (2 h)**	**4 – (3h)**
**M_n / M_w (Da)**	813 /2630	1080/5430	1500/8400	3136/10600
Mass fraction wt% below <500g/mol	1.90	0.13	0.06	0.04
**FTIR (unsaturated ketone C=O) peak intensity**	0.361	0.332	0.313	0.337
**Phenolic content (mmol/g lignin)**	0.681	0.559	0.4717	0.487
**Carboxylic acid content (mmol/g lignin)**	0.215	0.336	0.179	0.247

Table S4. Size exclusion chromatography (SEC) data. Molecular weights and dispersities of Oligomer Beech and Pine and SAAF lignin PA Birch and Pine and IBA Pine

Samples	Mn	Mw	PDI	Mass fraction wt% below <500g/mol
**Oligomers Beech**	543	800	1.488	17.32
**Oligomers Pine**	911	2460	2.608	2.48
**SAAF Lignin PA Pine**	1422	4400	3.094	2.30
**SAAF Lignin IBA Pine**	1690	5700	3.38	1.69
**SAAF Lignin PA Birch**	3136	10600	3.392	0.04

Table S5. ^{31}P NMR quantification of aliphatic, phenolic and carboxylic (COOH) hydroxyl groups. Lignins studied: extracted SAAF PA/IBA-lignins (3 h) and oligomers. Phenolic-OH includes phenolic groups on all aromatic rings regardless of substitution on position 5 and 2.

As the color change is triggered by the transfer of the phenolic proton to the dye, we first quantified the phenolic content of each lignin or oligomer sample using a phosphorus nuclear magnetic resonance (^{31}P NMR) protocol, developed by Meng *et al*. (*67*). This method allows to measure the hydroxyl contents of lignin materials, successfully identifying aliphatic, phenolic and carboxylic acid groups using phosphorus' large chemical shifts. As anticipated due to their shorter chains, oligomers exhibited a significantly higher phenolic content than extracted lignin: phenolic OH (oligomers) =1.446-1.306 mmol/g > phenolic OH (SAAF lignins) = 0.487-1.019 mmol/g. However, following hydrogenolysis lignin depolymerization, oligomers tend to retain lower concentrations of carboxylic acid (-COOH) functionality: COOH (oligomers) = 0.038-0.046 mmol/g < COOH (SAAF lignins) = 0.204-0.349 mmol/g.).

mmol/g lignin	Oligomer Pine	Oligomer Beech	SAAF Lignin				Kraft	AAF
			PA Pine	PA Birch	IBA Pine	IBA Birch		GA Birch
Phenolic OH	1.436	1.306	1.019	0.487	0.665	0.567	1.354	1.13
Aliphatic OH	2.391	1.937	2.020	2.130	0.998	1.185	0.729	2.83
COOH	0.038	0.046	0.349	0.247	0.211	0.204	0.184	0.82

Table S6. CIE L*a*b*, CIE Yxy, and color density (C.D.) values for color development after octadecanol color activity test. Phenolic, *equimolar quantities* of grinded developer and the dye OBD-2 were mixed in a vial in the presence of a binder (1-octadecanol, melting point = 80 °C) and heated to 100 °C for 30 second (dye OBD-2: developer = 1:1 mol phenol eq.). Std stands for standard deviation.

Phenolic content constant	*L**	*a**	*b**	*Y*	C. D.	Std
Octadecanol (100 mg)	70.60	-6.44	7.52	40.83	0.39	0.01
Octadecanol (100 mg) + OBD-2 (10 mg)	68.28	-5.64	8.93	57.85	0.41	0.00
BPA	16.01	9.48	4.32	2.25	1.65	0.01
H ester	17.78	5.89	5.39	2.50	1.60	0.00
G ester	66.23	-6.67	7.75	35.61	0.45	0.01
Oligomer Beech	36.47	1.30	9.26	8.13	1.09	0.08
Oligomer Pine	36.87	2.28	10.41	10.03	1.00	0.01
SAAF Lignin PA Birch	6.82	2.47	7.49	0.74	2.13	0.05
SAAF Lignin PA Pine	6.22	0.47	7.12	0.86	2.07	0.03
SAAF Lignin IBA Pine	4.15	0.68	5.22	0.31	2.34	0.08
SAAF Lignin IBA Birch	8.84	4.56	5.21	1.08	1.97	0.07
Kraft Lignin	30.44	0.11	10.89	6.4	0.90	0.03
GA Lignin	49.7	-1.61	10.86	19.74	0.70	0.02

Table S7. CIE L*a*b*, CIE Yxy, and color density (C.D.) values for color development. After octadecanol test performed at 100 °C *with a constant mass ratio* (dye OBD-2: developer = 1 (10 mg) : 2 (20 mg)) in 100 mg octadecanol matrix. Std stands for standard deviation.

Mass content	*L**	*a**	*b**	*Y*	C. D.	std
Octadecanol (100 mg)	70.6	-6.4	7.5	41.2	0.4	0.0
Octadecanol (100 mg) + OBD-2 (10 mg)	68.1	-5.6	8.9	38.6	0.4	0.0
BPA	3.6	11.6	7.1	0.5	2.3	0.1
H ester	4.8	1.8	2.1	0.5	2.3	0.1
G ester	23.4	3.7	4.5	3.9	1.4	0.0
Oligomers Beech	35.5	3.9	7.1	8.5	1.1	0.0
Oligomers Pine	36.9	2.2	10.7	8.2	1.1	0.0
SAAF Lignin PA Birch	14.5	-0.3	7.2	1.8	1.7	0.0
SAAF Lignin PA pine	8.1	1.3	7.5	0.9	2.1	0.0
SAAF Lignin IBA Pine	12.9	1.1	6.2	1.5	1.8	0.1
SAAF Lignin IBA Birch	12.1	6.5	4.4	2.1	1.7	0.2
Kraft Lignin	24.5	-1.7	10.1	6.6	1.2	0.05

Table S8. Phase separation domain sizes in a lignin (19 wt%)-PVA blend. Acquired using the software ImageJ on microscopy images from fig. 4.

	Particles Sizes (mm)				
	Kraft Lignin	Oligomers	IBA Lignin	PA Lignin	GA Lignin
	0.052	0.088	0.007	0.007	0.111
	0.023	0.016	0.011	0.008	0.076
	0.018	0.099	0.003	0.003	0.203
	0.007	0.012	0.007	0.011	0.084
	0.133	0.01	0.003	0.009	0.163
	0.053	0.069	0.005	0.005	0.156
Average size (mm)	0.048	0.049	0.006	0.007	0.132
Std deviation (mm)	0.046	0.041	0.003	0.003	0.050

Table S9. ERα-CALUX molar results table. EC50: 50% effect concentration; PC50: 50% effect of the E2 positive control; PC10: 10% effect of the E2 positive control.

**Compound**	**Highest induction***	**EC50**	**PC50**	**PC10**	**Cytotoxicity at†**
17β-estradiol (E2)‡	100 %	1.9E-11	1.9E-11	4.5E-12	1.0E-09
BPA	161 %	6.5E-07	4.1E-07	1.3E-07	3.6E-05
BPS	96 %	2.3E-06	2.4E-06	1.6E-06	1.7E-04
H ester	68 %		1.1E-04	2.3E-05	3.3E-04
Oligomer Beech	10 %			6.1E-05	1.2E-04
Kraft	34 %			1.5E-04	6.6E-04
G ester	16 %			1.5E-04	5.5E-04
PA Pine	21 %			2.7E-04	1.0E-03
PA Birch	< 1 %				1.0E-04
IBA Pine	< 2 %				3.3E-04

* Highest induction seen. Orange: Fitted top of the curve

† Green: Highest tested dose was not toxic.

‡ Average from three plates

Table S10. L-YES molar results table for color developers. EC50: 50% effect concentration; PC50: 50% effect of the E2 positive control; PC10: 10% effect of the E2 positive control.

**Compound**	**Highest induction***	**EC50**	**PC50**	**PC10**	**Cytotoxicity at†**
17β-estradiol (E2) ‡	100%	7.9E-11	7.9E-11	3.3E-11	7.7E-10
BPA	99%	1.1E-06	1.1E-06	4.6E-07	1.8E-05
BPS	103%	2.3E-06	4.3E-05	1.6E-05	1.7E-04
H ester	45%	2.1E-05		9.3E-06	3.3E-04
Oligomers Beech	<1%				9.9E-04
Kraft	<1%				1.3E-03
G ester	<1%				5.5E-04
PA Pine	8%			1.2E-03	1.0E-03
PA Birch	<1%				8.3E-04
IBA Pine	<1%				6.5E-04

* Highest induction seen. Orange: Fitted top of the curve
† Green: Highest tested dose was not toxic.
‡ Average from three plates

Table S11. L-YES molar results for the DFX sugar-based sensitizer. Test performed in pure water. EC50: 50% effect concentration; PC50: 50% effect of the E2 positive control; PC10: 10% effect of the E2 positive control.

Compound	Highest induction*	EC50	PC50	PC10	Cytotox at†
17β-estradiol (E2) ‡	100%	1.4E-10	1.4E-10	4.4E-11	4.8E-10
DFX	< 1 %				3.8E-03

* Highest induction seen. Orange: Fitted top of the curve
† Green: Highest tested dose was not toxic.
‡ Average from three plates

Table S12. ERα-CALUX molar results for the DFX sugar-based sensitizer. EC50: 50% effect concentration; PC50: 50% effect of the E2 positive control; PC10: 10% effect of the E2 positive control.

Compound	Highest induction*	EC50	PC50	PC10	Cytotox at†
17β-estradiol (E2) ‡	100%	1.9E-11	1.9E-11	4.5E-12	1.0E-09
DFX	<1%				2.4E-03

* Highest induction seen. Orange: Fitted top of the curve
† Green: Highest tested dose was not toxic.
‡ Average from eight plates

Table S13. Dynamic light scattering (p): diluted aqueous solution of PVA and oligomers hardwood previously grinded by hand or using a mortar (1 and 5 min). A smaller particle size results in an increase in the active surface area. The proton transfer from the developer to the leuco dye is likely enhanced as a result, providing superior developing performance.(*34*)

	**Z-average size**	**PDI**
**Raw powder**	> 1000 nm	0.7
**Hand mixing**	> 1000 nm	1
**Mortar mixing 1 min**	> 1000 nm	0.8
**Mortar mixing 5 min**	952 nm	0.8

Table S14. CIE L*a*b*, CIE *Yxy*, and color density (C.D.) values. Color development on paper without a sensitizer (S2.7. Formulation S1). Std stands for standard deviation.

	Lignin	*L**	*a**	*b**	*Y*	*x*	*y*	C. D.	std
25°C	BPA	89.54	0.33	3.19	75.31	0.319	0.335	0.123	0.008
	H ester	90.02	-0.20	2.91	76.34	0.318	0.335	0.117	0.003
	Oligomer Beech	71.86	33.63	41.51	43.45	0.464	0.378	0.362	0.022
	Oligomer Pine	74.85	4.10	18.34	48.03	0.359	0.366	0.319	0.035
	PA Pine	70.70	5.81	33.33	41.74	0.397	0.399	0.380	0.031
	PA Birch	52.02	9.63	8.56	20.1	0.359	0.343	0.697	0.042
	IBA Pine	75.38	5.68	27.97	48.89	0.382	0.385	0.311	0.026
	Kraft	67.32	10.03	36.38	37.06	0.415	0.402	0.436	0.081
80°C	BPA				74.98			0.1250	0.0004
	H ester				70.16			0.1539	0.0042
	Oligomer Beech				40.66			0.3909	0.0098
	Oligomer Pine				48.14			0.3187	0.0397
	PA Pine				33.30			0.4776	0.0054
	PA Birch				20.13			0.6963	0.0167
	IBA Pine				46.67			0.3310	0.0063
	Kraft				31.71			0.4988	0.0103
100°C	BPA				58.42			0.2335	0.0040
	H ester				16.94			0.7777	0.0945
	Oligomer Beech				38.28			0.4171	0.0085
	Oligomer Pine				40.88			0.3885	0.0012
	PA Pine				36.88			0.4332	0.0042
	PA Birch				19.07			0.7195	0.0069
	IBA Pine				45.97			0.3376	0.0080
	Kraft				30.46			0.5163	0.0086
120°C	BPA				18.91			0.7249	0.0465
	H ester				8.93			1.0498	0.0282
	Oligomer Beech				38.05			0.4196	0.0006
	Oligomer Pine				42.20			0.3747	0.0110
	PA Pine				34.45			0.4629	0.0149
	PA Birch				19.70			0.7054	0.0034
	IBA Pine				45.91			0.3381	0.0071
	Kraft				29.72			0.5277	0.0332
140°C	BPA				9.67			1.0146	0.0160
	H ester				5.98			1.2231	0.0194
	Oligomer Beech				37.7			0.4231	0.0153
	Oligomer Pine				40.21			0.3956	0.0024
	PA Pine				33.63			0.4736	0.0210
	PA Birch				20.71			0.6839	0.0124
	IBA Pine				46.60			0.3321	0.0248
	Kraft				30.36			0.5177	0.0040

Table S15. CIE L*a*b*, CIE Yxy, and color density (C.D.) values. Color development on paper with benzalphtalide as the sensitizer (S2.7. Formulation S2). Std stands for standard deviation.

	Lignin	*L**	*a**	*b**	*Y*	*x*	*y*	C. D.	std
25°C	BPA	91.32	0.284	3.229	79.20	0.319	0.335	0.101	0.010
	H ester	91.36	0.199	3.055	79.29	0.318	0.335	0.101	0.005
	G ester	86.90	1.641	3.090	69.81	0.321	0.3346	0.156	0.001
	Oligomer Beech	70.17	8.871	21.174	40.99	0.376	0.365	0.390	0.002
	Oligomer Pine	72.49	6.511	20.724	44.40	0.370	0.370	0.350	0.002
	PA Pine	75.71	4.637	26.599	49.43	0.377	0.383	0.306	0.031
	PA Birch	80.28	4.346	17.357	56.18	0.355	0.362	0.250	0.002
	IBA Pine	83.74	4.862	14.638	63.58	0.349	0.355	0.197	0.019
	Kraft	61.69	9.953	31.929	30.05	0.412	0.397	0.550	0.031
80°C	BPA				61.07			0.214	0.0069
	H ester				42.29			0.373	0.0011
	G ester				47.57			0.322	0.0012
	Oligomer Beech				40.99			0.387	0.0067
	Oligomer Pine				44.41			0.352	0.0065
	PA Pine				49.62			0.304	0.0004
	PA Birch				56.17			0.250	0.0062
	IBA Pine				63.58			0.197	0.0091
	Kraft				30.47			0.516	0.0068
100°C	BPA				5.02			1.299	0.0021
	H ester				10.00			1.000	0.0099
	G ester				25.65			0.591	0.0040
	Oligomer Beech				36.57			0.436	0.0044
	Oligomer Pine				33.84			0.471	0.0067
	PA Pine				19.24			0.716	0.0220
	PA Birch				25.71			0.589	0.0446
	IBA Pine				25.88			0.589	0.0137
	Kraft				28.54			0.545	0.0313
120°C	BPA				2.05			1.686	0.0628
	H ester				2.78			1.555	0.0140
	G ester				15.78			0.801	0.0701
	Oligomer Beech				14.77			0.831	0.0124
	Oligomer Pine				12.27			0.911	0.0140
	PA Pine				9.25			1.037	0.0191
	PA Birch				13.77			0.861	0.0039
	IBA Pine				8.51			1.070	0.0288
	Kraft				28.54			0.545	0.0198
140°C	BPA				1.83			1.737	0.0386
	H ester				1.80			1.743	0.0096
	G ester				12.42			0.905	0.0905
	Oligomer Beech				9.37			1.028	0.0204
	Oligomer Pine				7.03			1.153	0.0041
	PA Pine				6.73			1.172	0.0471
	PA Birch				7.78			1.109	0.0215
	IBA Pine				6.48			1.188	0.0590
	Kraft				19.07			0.702	0.0040

Table S16. CIE L*a*b*, CIE *Yxy*, and color density (C.D.) values. Color development on paper with diphenylsulfone as the sensitizer (S2.7. Formulation S3).

	Lignin	*L**	*a**	*b**	*Y*	*x*	*y*	C. D.	std
25°C	BPA	81.293	24.721	13.084	62.83	0.319	0.335	0.202	0.001
	H ester	78.432	-0.180	2.594	53.94	0.318	0.335	0.268	0.005
	Oligomer Beech	66.217	31.463	38.845	35.60	0.464	0.378	0.449	0.002
	Oligomer Pine	70.371	3.893	17.438	41.27	0.359	0.366	0.384	0.002
	PA Pine	70.697	5.806	33.333	41.74	0.397	0.399	0.380	0.031
	PA Birch	80.777	13.696	12.180	58.06	0.359	0.343	0.236	0.002
	IBA Pine	84.337	6.237	30.706	64.71	0.382	0.385	0.189	0.019
	Kraft	64.005	9.624	34.934	32.80	0.415	0.402	0.485	0.031
80°C	BPA				63.01			0.20061	0.00503
	H ester				32.85			0.48338	0.00106
	Oligomer Beech				36.73			0.43493	0.00668
	Oligomer Pine				38.87			0.41034	0.00648
	PA Pine				46.80			0.32972	0.00035
	PA Birch				56.95			0.24454	0.00618
	IBA Pine				58.88			0.23007	0.00912
	Kraft				33.45			0.4756	0.00675
100°C	BPA				12.75			0.89451	0.00500
	H ester				2.3			1.63835	0.00988
	Oligomer Beech				36.433			0.43852	0.00438
	Oligomer Pine				37.84			0.42208	0.00675
	PA Pine				24.44			0.61221	0.02202
	PA Birch				54.21			0.26736	0.04458
	IBA Pine				58.01			0.23659	0.01368
	Kraft				32.08			0.49451	0.03134
120°C	BPA				3.73			1.4279	0.00067
	H ester				1.24			1.90556	0.01399
	Oligomer Beech				28.96			0.53827	0.01242
	Oligomer Pine				28.88			0.53945	0.01399
	PA Pine				12.44			0.90522	0.01909
	PA Birch				36.75			0.43475	0.00387
	IBA Pine				20.55			0.68776	0.02877
	Kraft				33.33			0.47742	0.01977
140°C	BPA				1.08			1.96503	0.03864
	H ester				0.51			2.28686	0.00965
	Oligomer Beech				17.19			0.76504	0.02042
	Oligomer Pine				18.65			0.72933	0.00413
	PA Pine				7.05			1.15327	0.04709
	PA Birch				25.70			0.5903	0.02146
	IBA Pine				9.34			1.03215	0.05901
	Kraft				30.36			0.51766	0.00397

Table S17. CIE L*a*b*, CIE *Yxy*, and color density (C.D.) values. Color development on paper with DFX as the sensitizer (30 wet wt.%, S2.7. Formulation S4).

	Lignin	*L**	*a**	*b**	*Y*	*x*	*y*	C. D.	std
25°C	BPA	23.68	35.68	2.94	4.00	0.4620	0.2753	1.3976	0.0079
	H ester	53.52	16.80	1.10	21.53	0.3522	0.3146	0.6673	0.0219
	Oligomer Beech	72.97	4.89	13.25	45.13	0.3504	0.3549	0.3455	0.0019
	Oligomer Pine	74.11	6.91	19.79	46.87	0.3677	0.3669	0.3293	0.0176
	PA Pine	71.81	4.17	28.08	43.38	0.3821	0.3890	0.3628	0.0111
	PA Birch	72.34	3.00	21.89	44.17	0.3663	0.3764	0.3548	0.0024
	IBA Pine	74.85	4.50	25.08	48.05	0.3742	0.3804	0.3184	0.0117
	Kraft	54.41	21.35	30.09	22.36	0.4434	0.4016	0.6505	0.0047
80°C	BPA				5.20			1.28396	0.01758
	H ester				11.84			0.92642	0.00391
	Oligomer Beech				32.92			0.4825	0.00270
	Oligomer Pine				39.25			0.40613	0.00173
	PA Pine				37.92			0.42174	0.02825
	PA Birch				32.93			0.48232	0.00244
	IBA Pine				31.19			0.50599	0.00260
	Kraft				18.18			0.74025	0.00118
100°C	BPA				3.03			1.51765	0.00784
	H ester				2.91			1.53616	0.00793
	Oligomer Beech				24.15			0.61704	0.00974
	Oligomer Pine				14.88			0.82829	0.03736
	PA Pine				20.59			0.68628	0.00371
	PA Birch				22.71			0.64379	0.00314
	IBA Pine				13.67			0.86427	0.00665
	Kraft				12.77			0.8937	0.00187
120°C	BPA				0.7			2.1549	0.00000
	H ester				0.52			2.27848	0.00478
	Oligomer Beech				17.10			0.76695	0.00628
	Oligomer Pine				12.16			0.91588	0.03282
	PA Pine				10.11			0.99511	0.00285
	PA Birch				15.55			0.80869	0.02344
	IBA Pine				8.88			1.05306	0.04583
	Kraft				3.50			1.45687	0.04755
140°C	BPA				0.59			2.22428	0.00421
	H ester				0.57			2.24167	0.05900
	Oligomer Beech				12.24			0.91208	0.01120
	Oligomer Pine				10.57			0.97613	0.01645
	PA Pine				4.15			1.3816	0.00060
	PA Birch				6.47			1.18923	0.01350
	IBA Pine				5.53			1.25728	0.00079
	Kraft				3.15			1.50133	0.01130

Table S18. SAAF lignin extraction yields. With PA aldehyde and Birch wood.

**Birch - Cycles**	**1**	**2**	**3**	**4**	**5**
**Extracted lignin (g/g wood)**	0.120	0.045	0.021	0.017	0.021
**Extraction yield (wt% total lignin)**	53.6	20.1	9.3	7.6	9.4

Table S19: SAAF lignin extraction yields. With PA aldehyde and Pine wood.

**Pine - Cycles**	**1**	**2**	**3**
**Extracted lignin (g/g wood)**	0.205	0.073	0.030
**Extraction yield (wt% total lignin)**	66.5	23.7	9.8

Table S20: SAAF lignin extraction yield with isobutyraldehyde and pine wood.

Pine - Cycles	1	2	3
Extracted lignin (g/gwood)	0.170	0.092	0.039
Extraction yield (wt% total lignin)	0.566	0.305	0.129

List of abbreviations

AAF:	Aldehyde Assisted Fractionation
BPA:	Bisphenol A
BPS:	Bisphenol S
BPSIP:	4-hydroxy-4′-isopropoxydiphenylsulfone
CALUX:	Chemical Activated LUciferase gene eXpression
CD:	Color Density
CER:	Concentration-effect relationships
DBX:	Dibutylxylose
DCP:	3,5-Dichlorophenol
DFX:	Diformylxylose
DMSO:	Dimethylsulfoxide
DPX:	Dipropylxylose
DLS:	Dynamic Light Scattering
DSC:	Differential Scanning Calorimetry
E2:	17β-estradiol
FT-IR:	Fourier Transform Infrared spectroscopy
GA:	Glyoxylic Acid
GPC:	Gel Permeation Chromatography
IBA: I	sobutyraldehyde
LYES:	Lyticase-Yeast Estrogen Screen
NHND:	N-hydroxy-5-norbornene-2,3-dicarboximide
NMR:	Nuclear Magnetic Resonance
ODB-2:	2-Anilino-6-dibutylamino-3-methylfluoran
PA:	Propionaldehyde
PVA:	Polyvinyl alcohol
SAAF:	Sequential Aldehyde Assisted Fractionation
SEC:	Size Exclusion Chromatography
TGA:	Thermogravimetrical Analysis
THF:	Tetrahydrofuran
TMDP:	2-chloro-4,4,5,5-tetramethyl-1,3-2-dioxaphospholane

REFERENCES

1. H. Zollinger, *Color Chemistry: Synthesis, Properties and Applications of Organic Dyes and Pigments* (Wiley-VCH, 2003).

2. Z. Yoshida, T. Kitao, *Chemistry of Functional Dyes* (Mita Press, 1989).

3. A. S. Diamond, D. S. Weiss, *Handbook of Imaging Materials* (CRC Press, 2002).

4. "Thermal paper market (2025–2030): Size, share & trends analysis report by width (57mm, 80mm), by application (POS, tags & label, lottery & gaming, ticketing, medical), by technology (direct transfer, thermal transfer), by region, and segment forecasts" (ID: 978-1-68038-690-5, Grand View Research, 2025); www.grandviewresearch.com/industry-analysis/thermal-paper-market#.

5. Y. Takahashi, A. Shirai, T. Segawa, T. Takahashi, K. Sakakibara, Why does a color-developing phenomenon occur on thermal paper comprising of a fluoran dye and a color developer molecule? *Bull. Chem. Soc. Jpn.* **75**, 2225–2231 (2002).

6. Y. Yoshinari, M. Terasaki, Recycled toilet paper sensitizers, a novel source of contamination in rivers. *Environ. Chem. Lett.* **22**, 485–489 (2024).

7. M. Terasaki, K. Jozuka, M. Makino, Identification and accumulation of aromatic sensitizers in fish from paper recycling in Japan. *Environ. Toxicol. Chem.* **31**, 1202–1208 (2012).

8. M. K. Björnsdotter, J. de Boer, A. Ballesteros-Gómez, Bisphenol A and replacements in thermal paper: A review. *Chemosphere* **182**, 691–706 (2017).

9. S. Babu, S. N. Uppu, B. Martin, O. A. Agu, R. M. Uppu, Unusually high levels of bisphenol A (BPA) in thermal paper cash register receipts (CRs): Development and application of a robust LC-UV method to quantify BPA in CRs. *Toxicol. Mech. Methods* **25**, 410–416 (2015).

10. B. S. Rubin, Bisphenol A: An endocrine disruptor with widespread exposure and multiple effects. *J. Steroid Biochem. Mol. Biol.* **127**, 27–34 (2011).

11. K. L. Howdeshell, A. K. Hotchkiss, K. A. Thayer, J. G. Vandenbergh, F. S. vom Saal, Exposure to bisphenol A advances puberty. *Nature* **401**, 763–764 (1999).

12. R. R. Newbold, W. N. Jefferson, E. Padilla-Banks, Prenatal exposure to bisphenol A at environmentally relevant doses adversely affects the murine female reproductive tract later in life. *Environ. Health Perspect.* **117**, 879–885 (2009).

13. G. G. J. M. Kuiper, J. G. Lemmen, B. Carlsson, J. C. Corton, S. H. Safe, P. T. Van Der Saag, B. Van Der Burg, J.-Å. Gustafsson, Interaction of estrogenic chemicals and phytoestrogens with estrogen receptor β. *Endocrinology* **139**, 4252–4263 (1998).

14. L. N. Vandenberg, M. V. Maffini, C. Sonnenschein, B. S. Rubin, A. M. Soto, Bisphenol-A and the great divide: A review of controversies in the field of endocrine disruption. *Endocr. Rev.* **30**, 75–95 (2009).

15. European Commission, Commission regulation (EU) 2016/2235 amending annex XVII to regulation (EC) no. 1907/2006 of the European Parliament and of the Council concerning the Registration, Evaluation, Authorisation and Restriction of Chemicals (REACH) as regards bisphenol A. OJEU 337, 3–5 (2016).

16. Directorate-General for Health and Food Safety, "Commission adopts ban of bisphenol A in food contact materials" (European Commission, 2024); https://food.ec.europa.eu/food-safety-news-0/commission-adopts-ban-bisphenol-food-contact-materials-2024-12-19_en.

17. W. Sun, Y. Guo, X. Sun, Z. Liu, D. Luo, N. Huang, Z. Xu, J. Wu, Y. Wu, Alternatives exert higher health risks than bisphenol a on indo-pacific humpback dolphins. *Environ. Sci. Technol.* **58**, 63–74 (2023).

18. S. Eladak, T. Grisin, D. Moison, M. J. Guerquin, T. N'Tumba-Byn, S. Pozzi-Gaudin, A. Benachi, G. Livera, V. Rouiller-Fabre, R. Habert, A new chapter in the bisphenol A story: Bisphenol S and bisphenol F are not safe alternatives to this compound. *Fertil. Steril.* **103**, 11–21 (2015).

19. J. R. Rochester, A. L. Bolden, Bisphenol S and F: A systematic review and comparison of the hormonal activity of bisphenol A substitutes. *Environ. Health Perspect.* **123**, 643–650 (2015).

20. S. Zhang, Y. Zhou, J. Shen, Y. Wang, J. Xia, C. Li, W. Liu, K. Hayat, M. Qian, Early-life exposure to 4-hydroxy-4′-isopropoxydiphenylsulfone induces behavioral deficits associated with autism spectrum disorders in mice. *Environ. Sci. Technol.* **58**, 15984–15996 (2024).

21. J. B. Zimmerman, P. T. Anastas, Toward substitution with no regrets: Advances in chemical design are needed to create safe alternatives to harmful chemicals. *Science* **347**, 1198–1199 (2015).

22. G. Z. Miller, D. T. Pitzzu, M. C. Sargent, J. Gearhart, Bisphenols and alternative developers in thermal paper receipts from the U.S. market assessed by Fourier transform infrared spectroscopy. *Environ. Pollut.* **335**, 122232 (2023).

23. M. Eckardt, T. J. Simat, Bisphenol A and alternatives in thermal paper receipts - A German market analysis from 2015 to 2017. *Chemosphere* **186**, 1016–1025 (2017).

24. P. Vervliet, C. Gys, N. Caballero-Casero, A. Covaci, Current-use of developers in thermal paper from 14 countries using liquid chromatography coupled to quadrupole time-of-flight mass spectrometry. *Toxicology* **416**, 54–61 (2019).

25. A.-L. Demierre, H. Reinhard, S. Zeltner, S. Frey, Evaluating the efficiency of the 2020 ban of BPA and BPS in thermal papers in Switzerland. *Regul. Toxicol. Pharmacol.* **146**, 105526 (2024).

26. S. Khatun, H. Ferdous, S. Hossain, S. Sultana, I. Choi, Y.-S. Lee, Detection of endocrine disruptor bisphenol A and bisphenol S in Bangladeshi thermal paper receipts. *Environ. Sci. Proc.* **20**, 1 (2022).

27. A. M. Hormann, F. S. Vom Saal, S. C. Nagel, R. W. Stahlhut, C. L. Moyer, M. R. Ellersieck, W. V. Welshons, P. L. Toutain, J. A. Taylor, Holding thermal receipt paper and eating food after using hand sanitizer results in high serum bioactive and urine total levels of bisphenol A (BPA). *PLOS ONE* **9**, e110509 (2014).

28. G. Russo, F. Barbato, L. Grumetto, Monitoring of bisphenol A and bisphenol S in thermal paper receipts from the Italian market and estimated transdermal human intake: A pilot study. *Sci. Total Environ.* **599-600**, 68–75 (2017).

29. Z. Xu, L. Tian, L. Liu, C. G. Goodyer, B. F. Hales, S. Bayen, Food thermal labels are a source of dietary exposure to bisphenol S and other color developers. *Environ. Sci. Tech.* **57**, 4984–4991 (2023).

30. K. Pivnenko, D. Laner, T. F. Astrup, Dynamics of bisphenol A (BPA) and bisphenol S (BPS) in the European paper cycle: Need for concern? *Resour. Conserv. Recycl.* **133**, 278–287 (2018).

31. C. Liao, K. Kannan, Widespread occurrence of bisphenol A in paper and paper products: Implications for human exposure. *Environ. Sci. Technol.* **45**, 9372–9379 (2011).

32. K.-H. Choi, H.-J. Kwon, B.-K. An, Synthesis and developing properties of functional phenolic polymers for ecofriendly thermal papers. *Ind. Eng. Chem. Res.* **57**, 540–547 (2018).

33. J. Yun, H. S. Kang, B. K. An, Phenolic polymer-based color developers for thermal papers: Synthesis, characterization, and applications. *Ind. Eng. Chem. Res.* **60**, 9456–9464 (2021).

34. Y.-I. Jang, B.-K. An, Hyperbranched polyester copolymers for thermal printing papers: The effects of alkyl chain units in the polymer backbone on developing capability. *Polymer* **78**, 193–201 (2015).

35. J. D. Bos, M. M. H. M. Meinardi, The 500 Dalton rule for the skin penetration of chemical compounds and drugs. *Exp. Dermatol.* **9**, 165–169 (2000).

36. D. W. Connell, *Bioaccumulation of Xenobiotic Compounds* (CRC Press, 1990).

37. M. Ragnar, C. T. Lindgren, N.-O. Nilvebrant, pK_a-values of guaiacyl and syringyl phenols related to lignin. *J. Wood Chem. Technol.* **20**, 277–305 (2000).

38. L. Trullemans, S. F. Koelewijn, I. Scodeller, T. Hendrickx, P. Van Puyvelde, B. F. Sels, A guide towards safe, functional and renewable BPA alternatives by rational molecular design: Structure–property and structure–toxicity relationships. *Polym. Chem.* **12**, 5870–5901 (2021).

39. J. Ralph, C. Lapierre, W. Boerjan, Lignin structure and its engineering. *Curr. Opin. Biotechnol.* **56**, 240–249 (2019).

40. C. O. Tuck, E. Pérez, I. T. Horváth, R. A. Sheldon, M. Poliakoff, Valorization of biomass: Deriving more value from waste. *Science* **337**, 695–699 (2012).

41. W. Arts, I. Storms, J. Van Aelst, B. Lagrain, B. Verbist, J. Van Orshoven, P. J. Verkerk, W. Vermeiren, J. P. Lange, B. Muys, B. F. Sels, Feasibility of wood as a renewable carbon feedstock for the production of chemicals in Europe. *Biofuels Bioprod. Biorefin.* **18**, 365–377 (2024).

42. J. Wenger, V. Haas, T. Stern, Why can we make anything from lignin except money? Towards a broader economic perspective in lignin research. *Curr. For. Rep.* **6**, 294–308 (2020).

43. A. J. Ragauskas, G. T. Beckham, M. J. Biddy, R. Chandra, F. Chen, M. F. Davis, B. H. Davison, R. A. Dixon, P. Gilna, M. Keller, P. Langan, A. K. Naskar, J. N. Saddler, T. J. Tschaplinski, G. A. Tuskan, C. E. Wyman, Lignin valorization: Improving lignin processing in the biorefinery. *Science* **344**, 1246843 (2014).

44. J. Cheng, X. Zhou, C. Huang, C. G. Yoo, X. Meng, G. Fang, A. J. Ragauskas, C. Huang, Low-chromophore lignin isolation from natural biomass with polyol-based deep eutectic solvents. *Green Chem.* **26**, 8298–8314 (2024).

45. C. Crestini, H. Lange, M. Sette, D. S. Argyropoulos, On the structure of softwood kraft lignin. *Green Chem.* **19**, 4104–4121 (2017).

46. L. Shuai, M. T. Amiri, Y. M. Questell-Santiago, F. Héroguel, Y. Li, H. Kim, R. Meilan, C. Chapple, J. Ralph, J. S. Luterbacher, Formaldehyde stabilization facilitates lignin monomer production during biomass depolymerization. *Science* **354**, 329–333 (2016).

47. M. Talebi Amiri, G. R. Dick, Y. M. Questell-Santiago, J. S. Luterbacher, Fractionation of lignocellulosic biomass to produce uncondensed aldehyde-stabilized lignin. *Nat. Protoc.* **14**, 921–954 (2019).

48. T. Shibata, J. Semler, G. Gaesser, Sensitizer for heat sensitive paper coatings. US patent 5066633 (1991), p. 11.

49. K. Sato, T. Harada, Leuco dyes. US patent 4864024 (1989), p. 11.

50. A. O. Komarova, G. R. Dick, J. S. Luterbacher, Diformylxylose as a new polar aprotic solvent produced from renewable biomass. *Green Chem.* **23**, 4790–4799 (2021).

51. Y. M. Questell-Santiago, J. H. Yeap, M. Talebi Amiri, B. P. Le Monnier, J. S. Luterbacher, Catalyst evolution enhances production of xylitol from acetal-stabilized xylose. *ACS Sustainable Chem. Eng.* **8**, 1709–1714 (2020).

52. L. P. Manker, G. R. Dick, A. Demongeot, M. A. Hedou, C. Rayroud, T. Rambert, M. J. Jones, I. Sulaeva, M. Vieli, Y. Leterrier, A. Potthast, F. Maréchal, V. Michaud, H.-A. Klok, J. S. Luterbacher, Sustainable polyesters via direct functionalization of lignocellulosic sugars. *Nat. Chem.* **14**, 976–984 (2022).

53. S. Sun, G. De Angelis, S. Bertella, M. J. Jones, G. R. Dick, E. Amstad, J. S. Luterbacher, Integrated conversion of lignocellulosic biomass to bio-based amphiphiles using a functionalization-defunctionalization approach. *Angew. Chem. Int. Ed. Engl.* **63**, e202312823 (2024).

54. H. Zhang, X. Liu, S. Fu, Y. Chen, Fabrication of light-colored lignin microspheres for developing natural sunscreens with favorable UV absorbability and staining resistance. *Ind. Eng. Chem. Res.* **58**, 13858–13867 (2019).

55. H. Sadeghifar, A. Ragauskas, Lignin as a UV light blocker - A review. *Polymers* **12**, 1134 (2020).

56. H. Ji, P. Lv, Mechanistic insights into the lignin dissolution behaviors of a recyclable acid hydrotrope, deep eutectic solvent (DES), and ionic liquid (IL). *Green Chem.* **22**, 1378–1387 (2020).

57. H. Zhang, Y. Bai, B. Yu, X. Liu, F. Chen, A practicable process for lignin color reduction: Fractionation of lignin using methanol/water as a solvent. *Green Chem.* **19**, 5152–5162 (2017).

58. S. Y. Jeong, B. Koo, J. W. Lee, Structural changes in biomass (yellow poplar and empty fruit bunch) during hydrothermal and oxalic acid pretreatments and their effects on enzymatic hydrolysis efficiency. *Ind. Crop. Prod.* **178**, 114569 (2022).

59. X.-C. Cheng, Z. Qin, Q.-L. Yang, H.-M. Liu, X.-D. Wang, Y.-L. Liu, Sequential extraction of organosolv lignin from Chinese quince fruit: Structural features and antioxidant activities of the obtained fractions. *BioRes* **16**, 2714–2730 (2021).

60. N. Kumar, S. Vijayshankar, P. Pasupathi, S. Nirmal Kumar, P. Elangovan, M. Rajesh, K. Tamilarasan, Optimal extraction, sequential fractionation and structural characterization of soda lignin. *Res. Chem. Intermed.* **44**, 5403–5417 (2018).

61. B. Rietzler, M. Karlsson, I. Kwan, M. Lawoko, M. Ek, Fundamental insights on the physical and chemical properties of organosolv lignin from Norway spruce bark. *Biomacromolecules* **23**, 3349–3358 (2022).

62. A. O. Komarova, Z. J. Li, M. J. Jones, O. Erni, F. Neuenschwander, J. D. Medrano-García, G. Guillén-Gosálbez, F. Ois Maréchal, R. Marti, J. S. Luterbacher, Sustainable one-pot production and scale-up of the new platform chemical diformylxylose (DFX) from agricultural biomass. *ACS Sustainable Chem. Eng.* **12**, 12879–12889 (2024).

63. Imarc, "Bisphenol A prices, trend, chart, demand, market analysis, news, historical and forecast data report 2024 edition" (Report ID: SR112025A22449, Imarc, 2024); www.imarcgroup.com/bisphenol-a-pricing-report.

64. S. Dupoiron, M. L. Lameloise, M. Pommet, O. Bennaceur, R. Lewandowski, F. Allais, A. R. S. Teixeira, C. Rémond, H. Rakotoarivonina, A novel and integrative process: From enzymatic fractionation of wheat bran with a hemicellulasic cocktail to the recovery of ferulic acid by weak anion exchange resin. *Ind. Crop. Prod.* **105**, 148–155 (2017).

65. M. Chen, Y. Li, F. Lu, J. S. Luterbacher, J. Ralph, Lignin hydrogenolysis: Phenolic monomers from lignin and associated phenolates across plant clades. *ACS Sustainable Chem. Eng.* **11**, 10001–10017 (2023).

66. Y.-S. Fang, M.-H. Yang, L. Cai, J.-P. Wang, T.-P. Yin, J. Yu, Z.-T. Ding, New phenylpropanoids from *Bulbophyllum retusiusculum*. *Arch. Pharm. Res.* **41**, 1074–1081 (2018).

67. X. Meng, C. Crestini, H. Ben, N. Hao, Y. Pu, A. J. Ragauskas, D. S. Argyropoulos, Determination of hydroxyl groups in biorefinery resources via quantitative ^{31}P NMR spectroscopy. *Nat. Protoc.* **14**, 2627–2647 (2019).

68. R. Vendamme, J. Behaghel De Bueren, J. Gracia-Vitoria, F. Isnard, M. M. Mulunda, P. Ortiz, M. Wadekar, K. Vanbroekhoven, C. Wegmann, R. Buser, F. Héroguel, J. S. Luterbacher, W. Eevers, Aldehyde-assisted lignocellulose fractionation provides unique lignin oligomers for the design of tunable polyurethane bioresins. *Biomacromolecules* **21**, 4135–4148 (2020).

69. Y. Liao, S. F. Koelewijn, G. van den Bossche, J. van Aelst, S. van den Bosch, T. Renders, K. Navare, T. Nicolaï, K. van Aelst, M. Maesen, H. Matsushima, J. M. Thevelein, K. van Acker, B. Lagrain, D. Verboekend, B. F. Sels, A sustainable wood biorefinery for low-carbon footprint chemicals production. *Science* **367**, 1385–1390 (2020).

70. A. Ward-Askey, J. B. Cooper, M. E. Hobson, Improvements in thermal paper. EP patent 1677990A1 (2004), p. 22.

71. F. A. Houle, W. D. Hinsberg, M. Morrison, M. I. Sanchez, G. Wallraff, C. Larson, J. Hoffnagle, Determination of coupled acid catalysis-diffusion processes in a positive-tone chemically amplified photoresist. *J. Vac. Sci. Technol. B* **18**, 1874–1885 (2000).

72. Koehler Paper, "Conventional thermal papers for self-adhesive labels" (2024); www.koehlerpaper.com/en/products/Thermal-paper/TH_Labels.php.

73. N. Alwadani, P. Fatehi, Synthetic and lignin-based surfactants: Challenges and opportunities. *Carbon Resour. Convers.* **1**, 126–138 (2018).

74. S. Bertella, M. Bernardes Figueirêdo, G. De Angelis, M. Mourez, C. Bourmaud, E. Amstad, J. S. Luterbacher, Extraction and surfactant properties of glyoxylic acid-functionalized lignin. *ChemSusChem* **15**, e202200270 (2022).

75. S. Kubo, J. F. Kadla, The formation of strong intermolecular interactions in immiscible blends of poly(vinyl alcohol) (PVA) and lignin. *Biomacromolecules* **4**, 561–567 (2003).

76. A. O. Komarova, "Development of sustainable carbohydrate-based solvents by acetal functionalisation of biomass," thesis, Ecole Polytechnique Fédérale de Lausanne (EPFL), Lausanne (2023).

77. F. Ouyang, "Thermal paper technical specifications explained – The complete guide" (2025); https://pandapaperroll.com/thermal-paper-specifications/.

78. Koehler Paper, "Durability of Koehler thermal paper" (2025); www.koehlerpaper.com/media/docs/en/product-information/thermo/technische-infos/TH-Haltbarkeit-von-Koehler-Thermopapieren_GB.pdf.

79. M. W. Lewis, J. C. Rosenbaum, A. J. Herbert, P. Attri, Microencapsulated system for thermal paper. US patent 5741592 (1998), p. 11.

80. F. S. Chakar, M. R. Fisher, Thermally-responsive record material. US patent 2014263673A1 (2014), p. 19.

81. ISO, "ISO 19040-1 - Water quality - Determination of the estrogenic potential of water and waste water - Part 1: Yeast estrogen screen (*Saccharomyces cerevisiae*)" (ISO 19040-1:2018, 2018).

82. E. Simon, C. Riegraf, A. Schifferli, D. Olbrich, T. Bucher, E. L. M. Vermeirssen, Evaluation of three ISO estrogen receptor transactivation assays applied to 52 domestic effluent samples. *Environ. Toxicol. Chem.* **41**, 2512–2526 (2022).

83. OECD, "Test no. 455: Performance-based test guideline for stably transfected transactivation in vitro assays to detect estrogen receptor agonists and antagonists," in *OECD Guidelines for the Testing of Chemicals, Section 4* (OECD Publishing, 2021); https://doi.org/10.1787/9789264265295-en.

84. ISO, "ISO 23196 - Water quality - Calculation of biological equivalence (BEQ) concentrations" (ISO 23196:2022, 2022).

85. B. I. Escher, N. Bramaz, J. F. Mueller, P. Quayle, S. Rutishauser, E. L. M. Vermeirssen, Toxic equivalent concentrations (TEQs) for baseline toxicity and specific modes of action as a tool to improve interpretation of ecotoxicity testing of environmental samples. *J. Environ. Monit.* **10**, 612–621 (2008).

86. "German standard methods for the examination of water, waste water and sludge – Test methods using water organisms (group L) – Part 59: Algal growth inhibition test on microplate with unicellular green fresh water algae (L 59)" (DIN 38412-59, DIN Deutsches Institut für Normung, 2022).

87. L. Trullemans, S. F. Koelewijn, I. Boonen, E. Cooreman, T. Hendrickx, G. Preegel, J. Van Aelst, H. Witters, M. Elskens, P. Van Puyvelde, M. Dusselier, B. F. Sels, Renewable and safer bisphenol A substitutes enabled by selective zeolite alkylation. *Nat. Sustainability* **6**, 1693–1704 (2023).

88. A. M. Sotoca, T. F. H. Bovee, W. Brand, N. Velikova, S. Boeren, A. J. Murk, J. Vervoort, I. M. C. M. Rietjens, Superinduction of estrogen receptor mediated gene expression in luciferase based reporter gene assays is mediated by a post-transcriptional mechanism. *J. Steroid Biochem. Mol. Biol.* **122**, 204–211 (2010).

89. T. Smith, J. Guild, The C.I.E. colorimetric standards and their use. *Trans. Opt. Soc.* **33**, 73–134 (1932).

90. Konica Minolta, “Precise color communication – Color control from perception to instrumentation” (2007); www.konicaminolta.com/instruments/knowledge/color/pdf/color_communication.pdf.

91. M. Paulsson, J. Parkås, Review: Light-induced yellowing of lignocellulosic pulps – Mechanisms and preventive methods. *BioRes* **7**, 5995–6040 (2012).

92. ISO, “ISO 19040-3 - Water quality - Determination of the estrogenic potential of water and waste water - Part 3: In vitro human cell-based reporter gene assay” (ISO 19040-3: 2018, 2018).

93. E. L. M. Vermeirssen, C. Dietschweiler, I. Werner, M. Burkhardt, Corrosion protection products as a source of bisphenol A and toxicity to the aquatic environment. *Water Res.* **123**, 586–593 (2017).

94. A. O. Komarova, C. M. Warne, H. Pétremand, L. König-Mattern, J. Stöckelmaier, C. Oostenbrink, G. M. Guebitz, J. Luterbacher, A. Pellis, Xylose acetals - A new class of sustainable solvents and their application in enzymatic polycondensation. *ChemSusChem* **18**, e202401877 (2025).
